# African ancestry neurodegeneration risk variant disrupts an intronic branchpoint in *GBA1*

**DOI:** 10.1038/s41594-024-01423-2

**Published:** 2024-12-12

**Authors:** Pilar Álvarez Jerez, Peter Wild Crea, Daniel M. Ramos, Emil K. Gustavsson, Mandy Radefeldt, Andrey Damianov, Mary B. Makarious, Oluwadamilola O. Ojo, Kimberley J. Billingsley, Laksh Malik, Kensuke Daida, Sarah Bromberek, Fangle Hu, Zachary Schneider, Aditya L. Surapaneni, Julia Stadler, Mie Rizig, Huw R. Morris, Caroline B. Pantazis, Hampton L. Leonard, Laurel Screven, Yue A. Qi, Mike A. Nalls, Sara Bandres-Ciga, John Hardy, Henry Houlden, Celeste Eng, Esteban González Burchard, Linda Kachuri, Chia-Ho Lin, Douglas L. Black, Global Parkinson’s Genetics Program (GP2), Mary B. Makarious, Oluwadamilola O. Ojo, Mie Rizig, Caroline B. Pantazis, Hampton L. Leonard, Sara Bandres-Ciga, John Hardy, Henry Houlden, Mike A. Nalls, Andrew B. Singleton, Mina Ryten, Njideka U. Okubadejo, Cornelis Blauwendraat, Andrew B. Singleton, Steffen Fischer, Peter Bauer, Xylena Reed, Mina Ryten, Christian Beetz, Michael Ward, Njideka U. Okubadejo, Cornelis Blauwendraat

**Affiliations:** 1 Center for Alzheimer’s and Related Dementias, National Institute on Aging and National Institute of Neurological Disorders and Stroke, National Institutes of Health, Bethesda, MD USA; 2 Laboratory of Neurogenetics, National Institute on Aging, Bethesda, MD USA; 3 Department of Neurodegenerative Disease, UCL Queen Square Institute of Neurology, University College London, London, UK; 4 Genetics and Genomic Medicine, Great Ormond Street Institute of Child Health, University College London, London, UK; 5 Centogene, Rostock, Germany; 6 Department of Microbiology, Immunology and Molecular Genetics, The David Geffen School of Medicine, University of California, Los Angeles, Los Angeles, CA USA; 7 DataTecnica, Washington, DC USA; 8 College of Medicine, University of Lagos, Lagos, Nigeria; 9 Lagos University Teaching Hospital, Lagos, Nigeria; 10 Department of Medicine, New York University Langone School of Medicine, New York, NY USA; 11 Department of Neuromuscular Diseases, UCL Queen Square Institute of Neurology, University College London, London, UK; 12 UCL Movement Disorders Centre, University College London, London, UK; 13 Department of Biotherapeutic Sciences and Department of Medicine, University of California, San Francisco, San Francisco, CA USA; 14 Department of Epidemiology and Population Health, Stanford University School of Medicine, Stanford, CA USA; 15 Stanford Cancer Institute, Stanford University School of Medicine, Stanford, CA USA; 16 Aligning Science Across Parkinson’s (ASAP) Collaborative Research Network, Chevy Chase, MD USA; 17 UK Dementia Research Institute and Department of Clinical Neurosciences, University of Cambridge, Cambridge, UK; 18 Neurogenetics Branch, National Institute of Neurological Disorders and Stroke, Bethesda, MD USA

**Keywords:** RNA splicing, Genomics, Genome, Diseases

## Abstract

Recently, an African ancestry-specific Parkinson disease (PD) risk signal was identified at the gene encoding glucocerebrosidase (*GBA1*). This variant (rs3115534-G) is carried by ~50% of West African PD cases and imparts a dose-dependent increase in risk for disease. The risk variant has varied frequencies across African ancestry groups but is almost absent in European and Asian ancestry populations. *GBA1* is a gene of high clinical and therapeutic interest. Damaging biallelic protein-coding variants cause Gaucher disease and monoallelic variants confer risk for PD and dementia with Lewy bodies, likely by reducing the function of glucocerebrosidase. Interestingly, the African ancestry-specific *GBA1* risk variant is a noncoding variant, suggesting a different mechanism of action. Using full-length RNA transcript sequencing, we identified partial intron 8 expression in risk variant carriers (G) but not in nonvariant carriers (T). Antibodies targeting the N terminus of glucocerebrosidase showed that this intron-retained isoform is likely not protein coding and subsequent proteomics did not identify a shorter protein isoform, suggesting that the disease mechanism is RNA based. Clustered regularly interspaced short palindromic repeats editing of the reported index variant (rs3115534) revealed that this is the sequence alteration responsible for driving the production of these transcripts containing intron 8. Follow-up analysis of this variant showed that it is in a key intronic branchpoint sequence and, therefore, has important implications in splicing and disease. In addition, when measuring glucocerebrosidase activity, we identified a dose-dependent reduction in risk variant carriers. Overall, we report the functional effect of a *GBA1* noncoding risk variant, which acts by interfering with the splicing of functional *GBA1* transcripts, resulting in reduced protein levels and reduced glucocerebrosidase activity. This understanding reveals a potential therapeutic target in an underserved and underrepresented population.

## Abstract (web summary)

Here, the authors describe a noncoding genetic variant in *GBA1* specific to people of African ancestry that increases the risk of neurodegenerative diseases by interfering with the splicing of mRNA, resulting in lowered protein levels and activity.

## Main

Dementia with Lewy bodies (DLB) and Parkinson disease (PD) are believed to be caused by a combination of aging, environmental factors and genetics. Genetics has provided valuable insights into the underlying biology of disease. Damaging variants in multiple genes have been shown to cause disease and numerous variants have been associated with increased risk^1–3^. One particular gene of interest is *GBA1* (previously known as *GBA*), which encodes the lysosomal enzyme glucocerebrosidase (GCase). Damaging coding variants in *GBA1* increase the risk for PD and DLB across a wide spectrum of odds ratios (ORs)^4,5^. Interestingly, the phenotype of individuals with PD carrying *GBA1* variants is characterized by faster progression and a higher frequency of dementia compared to noncarriers^6,7^. It is most commonly hypothesized that *GBA1* mutations confer risk by reducing GCase activity. Furthermore, damaging *GBA1* variants are enriched in certain populations (for example, p.E365K in Northern Europeans and p.N409S in Ashkenazi Jews)^8^. Biallelic *GBA1* variants cause Gaucher disease, a lysosomal storage disorder that leads to a variety of clinical presentations.

In the human genome, *GBA1* is located on chromosome 1q22 and is adjacent to its pseudogene, known as *GBA1LP*. *GBA1LP* has very high sequence homology with *GBA1* (~96%). Consequently, accurately mapping short-read RNA and DNA sequencing in this region is a complex task^9,10^. Much is unknown about the potential function of *GBA1LP*; however, using long-read sequencing, which overcomes mapping issues in this genomic region, it is clear that *GBA1LP* is expressed at the RNA level and there is some evidence for protein expression^10^.

Recently, a PD *GBA1* risk signal was identified in the first African ancestry PD genome-wide association study (GWAS)^11^. The main index variant was remarkably common in West African populations with an estimated frequency of ~50% in West African PD cases and a reported OR of 1.58 (95% confidence interval (CI) = 1.37–1.80, *P* = 2.397 × 10^−14^) per allele. In addition, this variant was associated with an earlier age at onset of 2 years per allele (*β* = −2.004, s.e.m. = 0.57, *P* = 0.0005). Strikingly, the main index variant (rs3115534, NM_000157.4 (*GBA1*): c.1225-34C>A) is a noncoding variant reported to be the strongest expression (eQTL) and protein (pQTL) quantitative trait locus for *GBA1* in the African ancestry population^12,13^. We showed previously that there are no common coding variants or structural variants in linkage disequilibrium with rs3115543, implying a disease mechanism independent of protein-coding or genomic structural variants at this locus^11^.

Here, we elucidate the disease mechanism of an intronic *GBA1* PD risk variant seen as the first and major genetic risk factor in African ancestry populations. Additionally, we show that the index variant (rs3115534) causes abnormal splicing and processing of *GBA1* transcripts, only present in risk variant carriers.

## Results

### Functional dissection of the GBA1 African ancestry locus

**Fig. 1:**
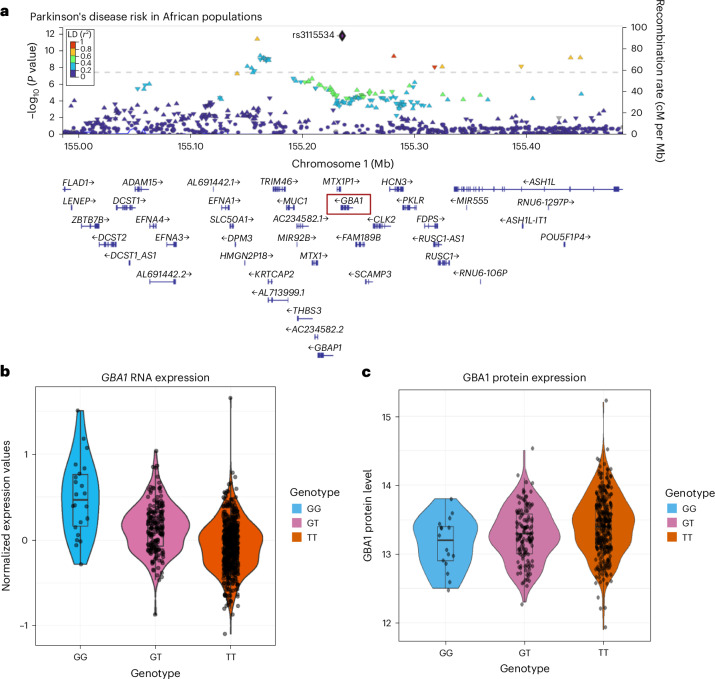
Overview of the African ancestry PD *GBA1* GWAS locus. **a**, LocusZoom plot showing rs3115534 as index variant (purple diamond) and located in intron 8 of *GBA1*. **b**, rs3115534 as eQTL for *GBA1* RNA expression from Kachuri et al. showing increased *GBA1* expression with G risk genotypes^12^. Genotype counts: GG, *n* = 22; GT, *n* = 185; TT, *n* = 537. **c**, rs3115534 as pQTL for GBA1 protein expression from Surapaneni et al. showing decreased protein levels with G risk genotypes^13^. Genotype counts: GG, *n* = 16; GT, *n* = 134; TT, *n* = 317. For all box plots, the center line represents the median, edges of the box represent the first and third quartiles and ends of bars represent the maximum and minimum (not including outliers).

Recently, a noncoding *GBA1* variant was reported to be associated with increased risk for PD in African ancestry individuals (Fig. 1a)^11^. This variant was also reported to be an eQTL and pQTL resulting in increased gene expression (Fig. 1b) and decreased protein expression (Fig. 1c)^12,13^. Using UK Biobank Olink data, we replicated the previously reported association between rs3115534 and GBA1 protein levels. After filtering for African ancestry, a total of 1147 samples remained with a GBA1 protein measure (43 GG, 351 GT and 753 TT). The G allele is significantly associated with lower GBA1 protein levels (*P* = 0.006, *β* = −0.074, s.e.m. = 0.027). (Supplementary Fig. 1). It is important to note that, in the reference genome used here (hg38), G is the reference allele for rs3115534, although G is the risk allele biologically. In contrast, the alternative allele in hg38 (rs3115534-T) functions as the nonrisk allele and the more common allele globally.

**Fig. 2:**
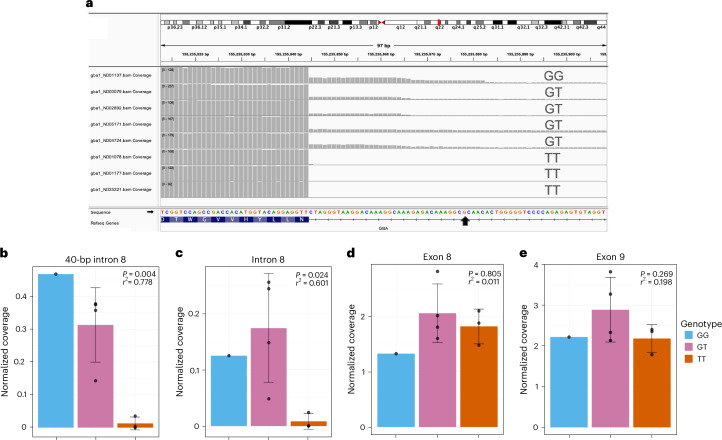
*GBA1* intron 8 expression is correlated with rs3115534 genotype. **a**, ONT long-read RNAseq of eight LCLs shows a consistent pattern where the rs3115534-G risk allele is associated with intron 8 expression and absent in homozygous T (nonrisk allele) individuals generated using IGV. **b**,**c**, Quantification of intron 8 expression is significantly associated with the G allele in both the 40-bp region before exon 9 (**b**) and the full intron 8 (**c**) (linear regression, *P* < 0.05). **d**,**e**, No significant differences were identified in the two neighboring exons 8 (**d**) and 9 (**e**). Coverage for all panels was normalized by dividing the mean depth by the total number of mapped reads per million (Methods). In **b**–**e**, a linear regression was run with GG + GT in one group versus TT. For all panels, genotype counts are as follows: GG, *n* = 1; GT, *n* = 4; TT, *n* = 3. Error bars represent the s.d. for all panels with the center at the mean. The unadjusted *r*^2^ is displayed.

Given the complexity of short-read DNA and RNA mapping in the *GBA1* region because of high sequence homology with *GBA1LP*, we generated Oxford Nanopore Technologies (ONT) long-read RNA sequencing (RNAseq) data to investigate this region in a more accurate and comprehensive manner. Long-read RNAseq data were generated from eight African ancestry lymphoblastoid cell lines (LCLs) from Coriell across risk genotypes (one homozygous risk, GG; four heterozygous risk, GT; three homozygous nonrisk, TT). Surprisingly, we identified that there was a clear enrichment of sequence reads in the intron 8 region proximal to exon 9 that was specific to carriers of the G risk allele (Fig. 2a and Supplementary Table 11).

Subsequently, de novo StringTie2 isoform calling suggested that there were multiple unannotated transcripts including one starting approximately 40 bp before exon 9 and two full-length *GBA1* isoforms including all of intron 8, hereafter collectively referred to as ‘intron 8 expression’ (Supplementary Fig. 2). Interestingly, the reported index variant rs3115534 is included in intron 8 expression (Fig. 2a, black arrow). We quantified the expression levels of this region and determined that expression levels of intron 8 were highly correlated with the presence of the G genotype (Fig. 2a–c and Supplementary Table 12). While the expression of the whole intron 8 (Fig. 2C) did not seem to follow a dose-dependent effect, this was perhaps because of only having a single homozygous G sample. In addition, no clear differences were observed for neighboring exons 8 and 9 (Fig. 2d,e) nor for total *GBA1* or *GBA1LP* expression (Supplementary Fig. 3). Importantly, every mapped sequence read exhibited a G allele, indicating their origin from the G risk haplotype. We manually changed the base in G reads to T to confirm that these reads mapped uniquely to *GBA1* and that the striking difference we observed was not an artifact of mismapping to *GBA1LP* driven by the rs3115534 variant. No differences in mapping were observed (Supplementary Fig. 4).

**Fig. 3:**
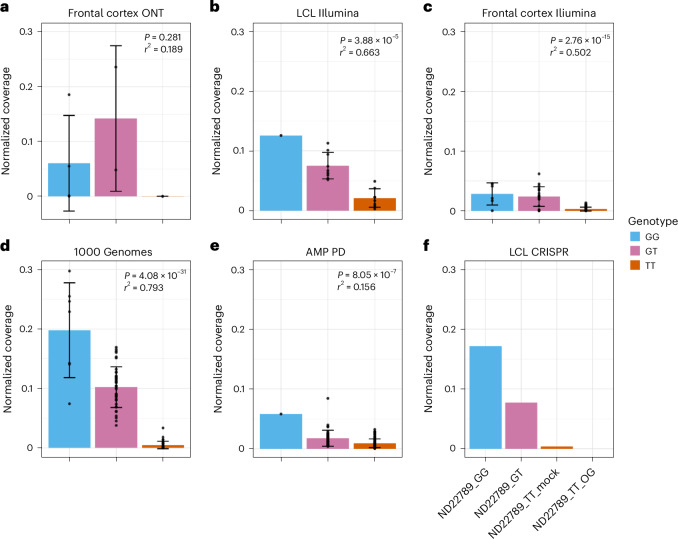
Increased intron 8 expression across datasets in G allele carriers. **a**, Intron 8 coverage from human frontal cortex sequenced with ONT (*n* = 8). Intron 8 expression is only present in G allele carriers but does not reach statistical significance (*P* = 0.281) likely because of a smaller sample size. **b**, Intron 8 coverage from LCLs (*n* = 18) sequenced with Illumina. Expression is significantly associated with the G allele (*P* = 3.88 × 10^−5^). **c**, Intron 8 expression from human frontal cortex sequenced with Illumina (*n* = 92). Expression is significantly associated with the G allele (*P* = 2.76 × 10^−^^15^). **d**, Intron 8 coverage from LCLs in the 1000 Genomes Project cohort (*n* = 88). Expression is significantly associated with the G allele (*P* = 4.08 × 10^−^^31^). **e**, Intron 8 coverage from blood in the AMP PD cohort (*n* = 148). Expression is significantly associated with the G allele (*P* = 8.05 × 10^−^^7^). **f**, CRISPR editing of LCLs showed that the rs3115534-G risk allele is significantly associated with intron 8 expression. Here, coverage is shown for the full allelic series of ND22789, a TT line originally CRISPR edited through to a GG line. Coverage for all panels normalized by dividing the mean regional depth by the total number of mapped reads per million (Methods). In **a**,**b**,**e**, a linear regression was run with GG + GT in one group versus TT. In **c**,**d**, a linear regression was run with GG, GT and TT in separate groups. Error bars represent the s.d. for all panels with the center at the mean. The unadjusted *r*^2^ is displayed.

Next, we examined whether intron 8 is also expressed in the human brain. Using ONT long-read RNAseq of eight African ancestry Human Brain Collection Core (HBCC) frontal cortex samples from different genotypes (four GG, two GT and three TT), we ran StringTie2 with the same methods as the LCLs. However, even when using our custom transcript models with our transcripts of interest, StringTie2 only identified the shorter intron 8 transcript in one GG carrier, likely because of lower expression of *GBA1* in the brain and lower RNA quality, as evidenced by lower RNA integrity number (RIN) values (Supplementary Tables 1 and 12). However, when looking at expression coverage plots, intron 8 expression was observed in all carriers of the G allele (GG and GT) but not in TT carriers (Fig. 3a, Supplementary Fig. 5 and Supplementary Table 11). Next, we generated Illumina short-read RNAseq for 18 LCLs (including the initial eight from above) and identified a similar albeit less pronounced increased read coverage across intron 8 (Fig. 3b and Supplementary Fig. 6). Likewise, similar enrichment in the intron 8 region correlated with the G allele of rs3115534 was seen in Illumina RNAseq data from the HBCC frontal cortex (Fig. 3c and Supplementary Fig. 7), 1000 Genomes Project LCL RNAseq (Fig. 3d) and Accelerating Medicine Partnership (AMP) PD blood-based RNAseq data (Fig. 3e). Importantly, with increased numbers, a likely allelic dosage effect becomes visible (that is, GG has significantly more intron 8 expression compared to GT carriers), although larger GG numbers in future datasets would be helpful to confirm this pattern (Supplementary Figs. 8 and 9 and Supplementary Table 11).

### GBA1 intron 8 expression unlikely encodes new protein product

Because of the comparatively lower *GBA1* expression in frontal cortex tissues and the greater accessibility of LCLs, the majority of subsequent experiments and analyses were conducted using LCLs. To validate the inclusion of *GBA1* intron 8, we designed primers specific to the most highly expressed region proximal to exon 9. Reverse transcription (RT)–PCR and subsequent Sanger sequencing validated the presence of this short transcript containing intron 8 in LCLs only in G risk variant carriers, confirming our initial findings (Supplementary Figs. 10 and 11). Next, we used cap analysis of gene expression and sequencing (CAGEseq), a technique often used to accurately pinpoint transcription start sites. The CAGEseq library preparation is dependent on the presence of an RNA cap, which is used to capture RNAs. We detected the canonical *GBA1* transcription start site; however, we did not identify a unique transcription start site within intron 8 (Supplementary Fig. 12). This indicates that the transcript containing intron 8 does not have a 5′ cap. However, the transcripts do have a poly(A) tail that can be detected with ONT long-read RNAseq.

The protein-coding capacity of this transcript was assessed using multiplexed and enhanced chemiluminescence (ECL) western blotting. Given that the highest intronic expression was observed close to exon 9, an C terminus antibody specific to amino acids 517–536 of the functional GCase protein was selected to ensure the capture of proteins that had a more downstream start site. Both multiplex and ECL methods consistently identified the native GCase protein but showed no other bands, indicating a lack of novel protein (Supplementary Fig. 13). Subsequent mass-spectrometry-based analysis of the gel region that was predicted to harbor the protein of the short transcript did not identify any known GBA1 peptides (Supplementary Table 13). These results suggest that the transcript is not protein coding and that the disease mechanism is likely to be RNA based.

### rs3115534 is the functional risk variant at the GBA1 locus

The close proximity of the index risk variant to the short transcript and the lack of other variants in linkage disequilibrium nearby made rs3115534 a strong candidate to be the functional effect variant for disease risk (Fig. 1a). To investigate this, we performed clustered regularly interspaced short palindromic repeats (CRISPR) editing on two LCLs: one in which the homozygous risk genotype (GG) was edited to be homozygous nonrisk (TT) and one in which the homozygous nonrisk genotype (TT) was edited to be homozygous risk (GG) (Supplementary Table 10). Genotyping qPCR was used to confirm the rs3115534 genotype in the edited lines. All lines contained the genotype as expected except one, in which full conversion from GG to TT was only partially successful and led to a heterozygous GT line. This edited line was excluded from downstream analysis.

Next, we performed ONT long-read DNA and RNAseq to confirm successful CRISPR editing and to assess the presence or absence of the intron 8 expression. ONT long-read DNA showed successful editing of line ND22789 from TT to GG and partial editing of line ND01137 from GG to GT, validating the qPCR results. ONT long-read RNAseq again showed the presence of intron 8 sequence reads but only in LCLs carrying a G allele. Quantification of the intronic reads for the full allelic series of the ND22789 edited line displayed an effect specific to the G allele (Fig. 3f), showing that the rs3115534-G allele is solely responsible for intron 8 transcription in LCLs. Quantification of both edited lines individually and collapsed by genotype is provided in Supplementary Fig. 14.

### Intron 8 is expressed across cell types in the human brain

Next, we assessed in which brain cell type this short transcript is expressed. Initial screening of frontal cortex brain single-nucleus RNAseq showed that overall *GBA1* expression is too low to accurately assess transcript expression and intron 8 expression was not observed (Supplementary Fig. 15). Therefore, we used an enrichment strategy with probes targeting the *GBA1* region in the single-nucleus complementary DNA (cDNA) library. After enrichment, we extracted *GBA1* transcripts and performed long-read ONT RNAseq on the single-nucleus libraries from one GG carrier and one TT carrier. Sequencing showed clear enrichment for *GBA1* and *GBA1LP* transcripts (Supplementary Table 14). *GBA1* and *GBA1LP* were ubiquitously expressed across major brain cell types (Supplementary Fig. 16a). Supporting our previous results, reads covering intron 8 were predominantly identified in the GG carrier as shown above (Supplementary Fig. 16a,b). When assessing the expression of the intron 8 region across cell types, the region was ubiquitously expressed across cell types, similar to the full *GBA1* transcript.

### rs3115534 is located in a key GBA1 intron 8 branchpoint

**Fig. 4:**
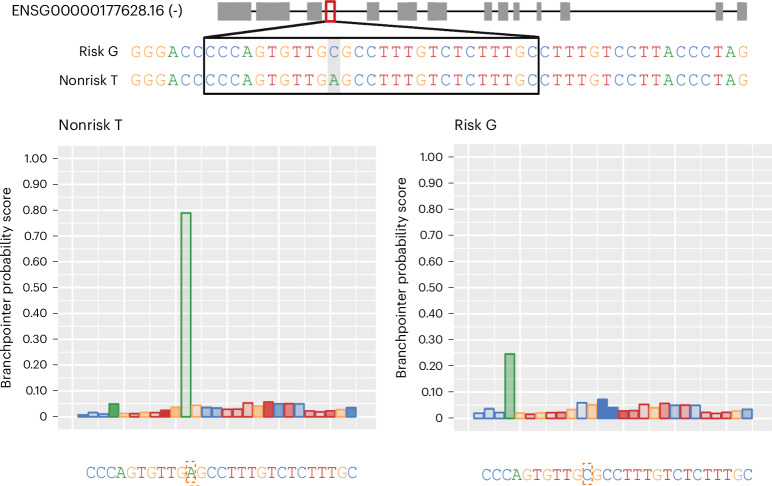
The *GBA1* intronic rs3115534 variant acts as a splicing branchpoint. The causal variant rs3115534 (highlighted in gray on the top and in red dashed box on the bottom) in intron 8 is located in the key splicing branchpoint according to Branchpointer. When rs3115534 is in a nonrisk state (T), on the antisense strand, the A allele functions as a branch site for the spliceosome, whereas, in the risk state (G), on the antisense strand, the C allele disrupts this branch site resulting in abnormal splicing.

To investigate potential functional downstream consequences of rs3115534, we explored several in silico algorithms. RegulomeDB analysis to assess the effect of rs3115534 on motifs and genome accessibility reported that this variant is an eQTL and is located in a region of open chromatin. However, no transcription factor motifs are affected by this variant. Sequence conservation analysis showed that the T allele (nonrisk) is highly conserved across vertebrates and humans are the only vertebrates harboring G as a reference allele (Supplementary Fig. 17). Interestingly, when analyzing the 5-methylcytosine DNA modifications in the ONT long-read DNA data of CRISPR-edited LCLs, we identified that the G allele is methylated. When rs3115534 is a T, the methylation is lost (Supplementary Fig. 18). This change in methylation status was confirmed when ONT sequencing was performed on the initial LCLs (Supplementary Fig. 19) and two additional frontal cortical brain samples (Supplementary Fig. 20). Lastly, given that rs3115534 was close to an exon (34 bp), we also assessed the variant’s potential to disrupt splicing. We used two complementary approaches: (1) SpliceAI, which is based on a deep neural network that accurately predicts splice junctions from pre-mRNA transcript sequences, and (2) Branchpointer and AGAIN, which are algorithms that are driven by an understanding of splicing biology. Using SpliceAI, no significant score of interest (<0.2) was identified. However, using Branchpointer and AGAIN, we identified that this variant is located within the key intronic branchpoint sequence of intron 8. The rs3115534-G allele (risk, C in the minus strand) is likely disrupting the splicing process by replacing the key A base (nonrisk, T in the positive strand) (Fig. 4).

This explains our initial finding from the ONT long-read RNAseq, which suggested that new transcripts (intron 8 containing) were transcribed only from the G risk haplotype (Supplementary Fig. 2). The ‘shorter’ transcripts, containing the part of intron 8 close to exon 9, are potentially spliced at the AG sequence (CT on sense strand) 6 nt upstream of rs3115534 and are sequenced as a short transcript given that they retain the poly(A) tail. The longer transcripts, containing the full nonspliced intron 8, are sequenced in full and also arise exclusively from the G risk haplotype. Here, intron 8 is partially retained, likely because of premature splicing of the intron occurring as a result of branchpoint disruption in the presence of the G (risk) allele in a highly conserved nucleotide position.

Next, we investigated the branch sites engaged with U2 small nuclear ribonucleoprotein (snRNP) in human 293Flp-in cells, which we determined to be homozygous for rs3115534-T (nonrisk). Using previously generated data^14^, we found further evidence that rs3115534-T is the main branchpoint nucleotide in intron 8 of *GBA1*. Different branch site datasets, obtained with constitutive (SF3A2) or associated (RBM5 and RBM10) U2 snRNP proteins, all predominantly used the adenine nucleotide at rs3115534-T as a branchpoint (Supplementary Fig. 21), confirming the bioinformatic branchpoint prediction algorithms.

### Assessing downstream effects of the branchpoint disruption

**Fig. 5:**
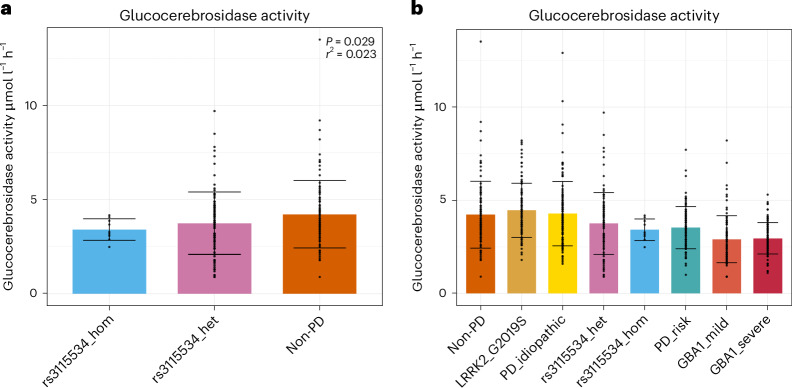
Measuring GCase activity across *GBA1* genotypes. **a**, GCase activity was measured across rs3115534*GBA1* genotypes showing a significant dose G-allele-dependent reduction across genotypes (*P* = 0.029). A linear regression was run with GG, GT and TT in separate groups. The unadjusted *r*^2^ is displayed. **b**, When measuring GCase activity across multiple heterozygous *GBA1* genotypes, it appears that rs3115534-GT and rs3115534-GG reduce GCase activity to similar levels to heterozygous PD risk variants (p.E365K and p.T408M) but remain higher than heterozygous *GBA1* Gaucher disease-causing variants (*GBA1*_mild, such as p.N409S, and *GBA1*_severe, such as p.L483P). Note that **a** was extracted from **b** and the same data are included in both. Group counts are as follows: non-PD, *n* = 97; LRRK2_G2019S, *n* = 95; PD_idiopathic, *n* = 122; rs3115534_het, *n* = 99; rs3115534_hom, *n* = 9; PD_risk, *n* = 99; *GBA1*_mild, *n* = 90; *GBA1*_severe, *n* = 99. Error bars represent the s.d. for all panels.

**Fig. 6:**
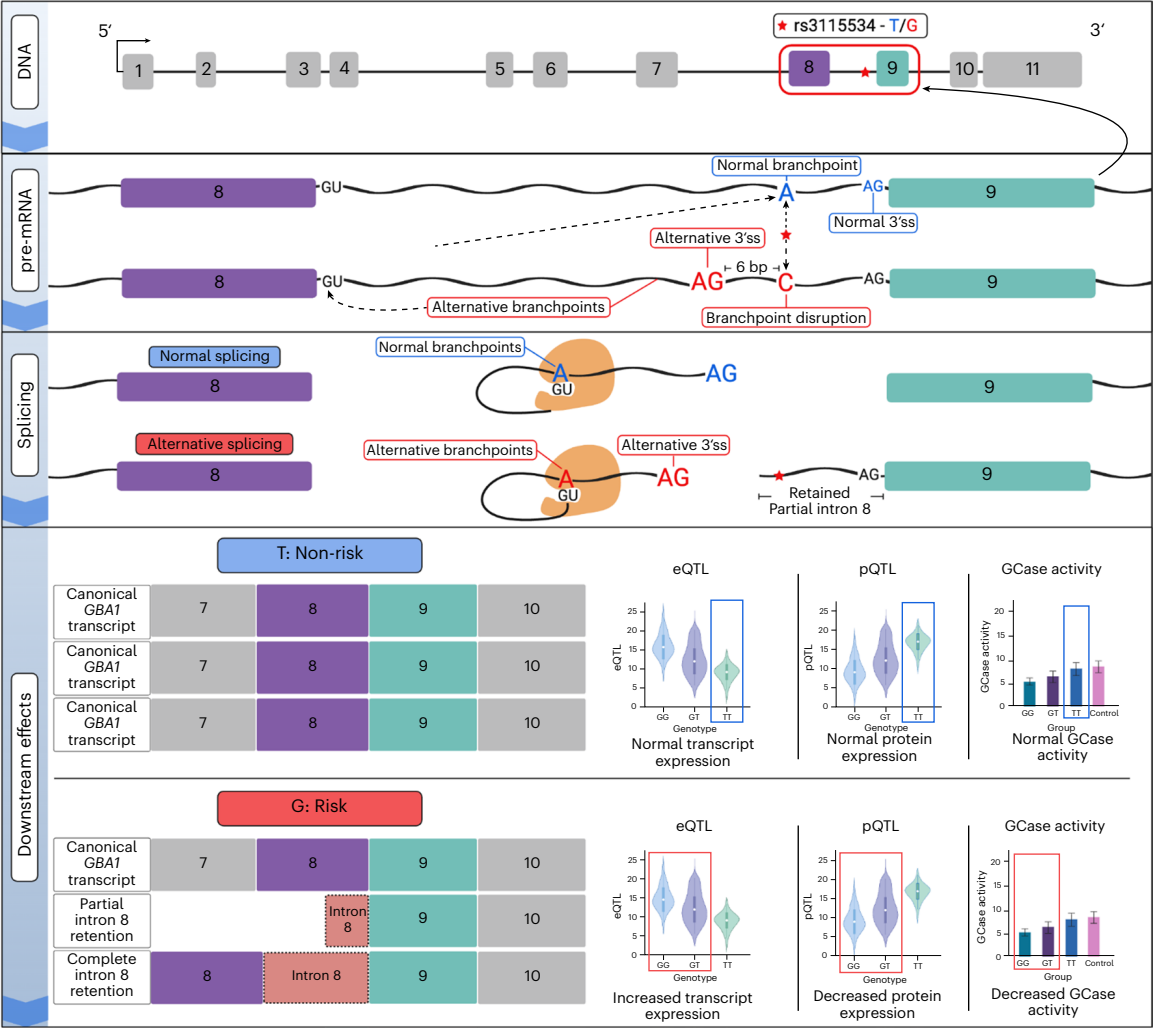
Suggested variant-to-function hypothesis of rs3115534. rs3115534-T is transcribed to pre-mRNA as A, a highly conserved branchpoint nucleotide. However, rs3115534-G, which confers elevated PD risk, is instead transcribed to C, causing the observed branchpoint disruption (rs3115534, denoted as red star). This single-nucleotide change in intron 8 impacts splicing by disrupting the normal binding from the adenosine branchpoint nucleotide and the 5′ splice site (GU). Subsequently, an alternative branchpoint is used, uncovering an alternative 3′ splice site upstream of the normal splice site immediately proximal to exon 9 and resulting in partial and complete intron retention and fewer functional *GBA1* mRNA transcripts. Downstream, abnormal splicing of *GBA1* leads to reduced GCase protein and subsequent lower GCase activity, which is a known pathomechanism of PD and DLB. ss, splice site. Generated with BioRender.com.

Given these results, we also wanted to assess potential consequences on GCase activity levels. Using Centogene’s CentoCards we compared GCase activity across individuals without PD, individuals with idiopathic PD, heterozygous *GBA1* carriers with PD-coding risk variants (p.E365K, p.T408M), heterozygous *GBA1* carriers with mild Gaucher disease-coding risk variants (p.N409S), heterozygous *GBA1* carriers with severe Gaucher disease-coding risk variants (including p.L483P) and rs3115534 variant carriers in heterozygous (GT) and homozygous state (GG). Using these data, a significant dose-dependent reduction in GCase activity correlated with the rs3115534-G risk genotype was observed when running a linear regression across all three groups (*P* = 0.029, *β* = −0.449, s.e.m. = 0.205) (Fig. 5a). Notably, the G-allele-associated reduction in GCase activity is similar to PD-coding risk variants that do not cause Gaucher disease (rs3115534_het versus PD_risk, *P* = 0.1333, *β* = −0.224, s.e.m. = 0.201; one-sided *P* value test) and higher than the Gaucher disease-causing variants (rs3115534_het versus GBA1_mild, *P* = 1.00 × 10^−4^, *β* = −0.846, s.e.m. = 0.215 and rs3115534_het versus GBA1_severe *P* = 1.52 × 10^−5^, *β* = −0.800, s.e.m. = 0.186; one-sided *P* value tests), confirming that the reduction in GCase is not enough to cause Gaucher disease (Fig. 5b). Supplementary Table 11 provides full details on statistical testing. Combined with our other results, these data suggest that, while no new protein product is made, the risk allele disrupts normal splicing and creates a new transcript that is likely nonfunctional, leading to a decrease in overall protein levels and, therefore, lower GCase activity (Fig. 6).

Lastly, we wanted to assess whether other variants in the *GBA1* region potentially affect branchpoint sequences. When exploring all *GBA1* variants from gnomAD (*n* = 1,625), three variants were identified to be of interest by the AGAIN algorithm, including the rs3115534 variant (Supplementary Table 15). The two other variants, rs140335079 and rs745734072, did not show a similar Branchpointer pattern (Supplementary Fig. 22). When exploring intron expression for rs140335079 in AMP PD, we did not identify any differences across genotypes (Supplementary Fig. 23). No RNAseq data were available for rs745734072, given that it is a rare variant and only identified in the South Asian population.

## Discussion

Most of the current genetic, genomic and functional knowledge in the neurodegeneration field is based on European ancestry findings. Recent efforts are showing great progress in making genomics more diverse, including GWAS reporting in East Asia, South Asia, Latin America and Africa^11,15–17^. In the past decade, GWAS has been the workhorse in genetics and has shown tremendous progress in the identification of genomic regions associated with traits and diseases^18^. However, one of the main limitations of GWAS is that it cannot pinpoint the exact mechanism, causal gene or variant in associated genomic regions. Therefore, follow-up methods such as QTL analysis and functional studies are used to fill this gap.

Recent progress in African ancestry PD genetics identified a noncoding *GBA1* risk variant associated with disease risk and an earlier age at onset^11^. Here, we show extensive follow-up of genomic and transcriptomic data from this region, pinpoint the functional variant and highlight the likely variant-associated mechanism. Using long-read RNAseq, we identified intron 8 retention, which was enriched in close proximity to the reported index variant rs3115534. Expression levels of intron 8 were correlated with G allele dosage and very low or absent in TT carriers. Given the high sequence overlap between *GBA1* and *GBA1LP*, it is unlikely that this expression is simply a sequence mapping artifact. The absence of a CAGEseq peak in this intronic region and the lack of a detectable additional protein isoform suggest that the risk mechanism is RNA based. CRISPR experiments showed that the reported index variant rs3115534 is the variant responsible for directly changing transcription. Importantly, given the notable in silico evidence for an intronic branchpoint disruption of rs3115534, it is clear that this is the cause of the intron 8 expression.

RNA splicing is the process where pre-mRNA is transformed into mRNA by removing introns. This complex process depends on the donor site (GT), the acceptor site (AG) and the branch site (often a sequence motif including an adenine base, known as the branchpoint). Disruption of any of these sequences causes missplicing, which typically results in reduced functional mRNA. Mutations in the branchpoint sequence motif are known to contribute to disease^19–22^. In the case of rs3115534, the A allele (nonrisk, T in the sense strand) is part of the key branch site sequence of intron 8; therefore, when rs3115534 is mutated to the C allele (risk, G in the sense strand) partial splicing disruption occurs (Fig. 6). This disruption results in partial intron 8 retention in some *GBA1* transcripts. This becomes particularly clear when investigating the long-read sequencing data showing enrichment of sequence reads close to exon 9 that start at the next intronic AG acceptor site 39 nt before exon 9, which is often seen in other branch site sequence disruptions.

Our finding that branchpoint disruption may confer disease risk highlights a potential mechanism for increased disease risk. All prior knowledge of *GBA1* disease mechanisms has attributed damaging coding variation to reduced GCase activity, leading to disease risk. However, there is some evidence that supports potential alternative mechanisms^23^. Here, we show that the branchpoint disruption likely causes splicing dysregulation, which results in increased risk by lowering the protein levels (pQTL) and, therefore, reducing the GCase activity correlated with genotype dosage. This is consistent with the hypothesis that reduction in GCase activity is the pathomechanism underlying PD and DLB and highlights a potential therapeutic target for African ancestry individuals.

Importantly, there are several inherent limitations of the study, many of which are driven by the lack of ancestrally diverse tissue, cellular and data resources. First, given the high sequence homology between *GBA1* and *GBA1LP* and the low expression levels of the potential short transcript isoforms, we cannot exclude its protein-coding potential. It could be possible that this isoform is too lowly expressed or is degraded too quickly for the antibody to detect the protein expression or to be seen on generalized mass spectrometry analysis. Second, although we measured GCase activity to assess potential downstream effects of the rs3115534 variant and several other *GBA1* genotypes, this assay remains very variable across samples and tissues. Here, we include a large collection of samples and observed expected effects across *GBA1* genotypes and, thus, believe that the results are robust, although access to additional biological samples in the future will allow us to repeat the experiment at higher power and greater resolution. In addition to larger sample collections, research across different cell types, especially including brain-related cell types, will allow us to measure the exact reduction in GCase levels, assess how the branchpoint disruption behaves across cell types and determine what the downstream consequences are of the reduction in GCase protein level. Ongoing recruitment efforts in the Global Parkinson’s Genetics Program (GP2; https://gp2.org/) aim to fill that gap^24^.

In summary, we report the rapid translation of a previously identified African ancestry GWAS locus and show the power of genetic diversity, which can result in valuable new biological insights into well-studied genes such as *GBA1*. We provide compelling evidence that rs3115534-G is the causal variant and show that the likely functional mechanism is a disruptive allelic change in the intronic branchpoint sequence that disrupts splicing, resulting in reduced protein levels. This shows how a common GWAS variant can be functionally explained by an intronic branchpoint sequence alteration, a potentially underexplored mechanism for GWAS. Overall, this implies that rs3115534-G is the most common damaging *GBA1* variant with a minor allele frequency of over 20% in certain populations and for which a substantial number of West African PD cases are heterozygous (40%) or homozygous (13%) risk carriers^11^. There is no evidence that this variant causes Gaucher disease, a condition that is remarkably infrequent in African ancestry groups, which is likely because of the magnitude of the biological effect of this variant, where the result is only a partial reduction of protein levels and GCase activity. Interestingly, this is an alternative mechanism for increased disease risk at *GBA1* and no other variants in *GBA1* were identified to act through a similar mechanism. The frequency of this risk variant, its mapping as the functional allele at this risk locus and the variant’s mode of action at an intronic branchpoint make this an attractive candidate for precision-based therapeutics in a remarkably underserved population. This work underscores the scientific and societal importance of working in groups underrepresented in research, as well as the need for the generation of biological and data resources in these groups, and marks the start of the realization of the mission of GP2.

## Methods

All our research complies with the relevant ethical regulations. The work was covered by local institutional review board approval at each site involved.

### Biosamples used for assessment of effects of *GBA1*rs3115534

To assess the potential molecular downstream effects of the noncoding *GBA1* variant rs3115534, we used data from GP2 data release 5 (https://gp2.org/, 10.5281/zenodo.7904832) and identified variant carriers of interest with matching LCLs at the Coriell Institute for Medical Research (https://www.coriell.org/). In addition, we accessed brain tissue samples from the HBCC with and without the noncoding *GBA1* variant (rs3115534). A full overview of included samples and their demographics is provided in Supplementary Table 1. Note that the genome reference allele (hg38) is G for rs3115534, which is also the risk allele, and T for the alternative nonrisk allele. Given that *GBA1* is transcribed from the antisense strand, C is risk and A is nonrisk on the RNA level.

### DNA extraction from cell pellets and brain tissue samples

DNA was extracted from LCLs (2 × 10^6^ cells) following the Pacific Biosciences (PacBio) high-molecular-weight (HMW) DNA extraction cultured cells protocol with the Nanobind tissue kit (PacBio, 102-203-100). The extraction occurred on a KingFisher Apex System (Thermo Fisher Scientific, 5400920). For brain tissue, 40 mg of frontal cortex tissue was cut and manually homogenized with a TissueRuptor (Qiagen, 9002755) in buffer CT (Pacbio, 102-280-300). Then, the DNA was extracted following the PacBio Apex Nanobind Tissue Big DNA protocol with the Nanobind tissue kit (PacBio, 102-203-100) using the KingFisher Apex System (Thermo Fisher Scientific, 5400920). The DNA from LCLs and brain tissue was quantified using the Qubit double-stranded DNA (dsDNA) BR assay (Invitrogen, Q32850) and sized with a Femto Pulse System (Agilent, M5330AA). The samples then underwent a size selection to remove fragments under 15 kb using the short-read eliminator kit (Pacbio, 102-208-300). After size selection, the DNA was sheared to a target size of 30 kb using the Megaruptor 3 (Diagenode, B060100003) with Fluid+ needles (Diagenode, E07020001) at speed 45 for two cycles. DNA was then quantified and resized with the Qubit dsDNA BR assay and the Femto Pulse system. Samples needed to have at least 4.5 μg of DNA and be 20–40 kb in size to take forward into library preparation, as previously described^25,26^.

### ONT DNA library preparation and sequencing

Sequencing libraries were prepared with the ONT SQK-LSK110 kit and 400 ng of prepared library per sample was loaded onto R9.4.1 flow cells on ONT’s PromethION device with Minknow 22.10.7 software. Samples were sequenced over 72 h with 1–2 additional library loads per flow cell. Sequencing data resulted in an average coverage of 30× and an N50 of around 30 kb per sample.

### RNA extraction from cell pellets and brain tissue samples

RNA was extracted from LCLs (5 × 10^6^ cells) and brain tissue (40 mg) using the RNA Direct-zol miniprep kit and its corresponding protocol (Zymo Research, R2050, https://files.zymoresearch.com/protocols/_r2050_r2051_r2052_r2053_direct-zol_rna_miniprep.pdf). In short, samples were resuspended in 600 μl of TRI reagent and an equal volume of 100% ethanol. Brain tissue needed additional homogenization with a Dounce homogenizer during the resuspension steps. Each mixture was then transferred into a Zymo-Spin IICR Column, centrifuged and transferred to a new collection tube. DNase I treatment was performed using the recommended guidelines. Washing steps were performed with Zymo Research’s Direct-zol RNA prewash and RNA wash buffer according to the protocol. Finally, RNA was eluted in RNase-free water and quality control was performed on Agilent’s TapeStation 4200. All cell lines had an RIN > 9, while brain RNA had RINs between 5.1 and 8.6 (Supplementary Table 1).

### ONT cDNA library preparation and sequencing

First, 200 ng of total RNA from the LCLs and brain tissue was prepared for sequencing using ONT’s cDNA-PCR SQK-PCS111 library preparation kit and protocol with modifications. This library preparation relies on an oligo(dT)-based poly(A) selection. The protocol modifications included an additional bead cleanup after RT with 11.25 μl of RNAse-free XP beads (Beckman Coulter, A63987), short fragment buffer washes (ONT, PCS111 kit) and elution into 22.5 μl of elution buffer. PCR settings during amplification were adjusted to set the annealing steps to 12 cycles. After library preparation, total RNA was quantified using the Qubit dsDNA high-sensitivity (HS) assay it (Invitrogen, Q32851). Then, 22 fmol of prepared library was loaded onto R9.4.1 PromethION flow cells and sequenced for 72 h using the Minknow 22.10.7 software.

### Single-nucleus cDNA library preparation

Nuclei from frozen brain tissue were prepared using a modified homogenization protocol^27,28^. Briefly, 30–50 mg of tissue was homogenized in a Dounce homogenizer with 20 strokes of the loose pestle and 20 strokes of the tight pestle with 1× lysis buffer (10 mM Tris-HCl (Sigma-Aldrich, T2194-1L), 10 mM NaCl (Sigma-Aldrich, 5922C-500ML), 3 mM MgCl_2_ (Sigma-Aldrich, M1028-100ML), 0.1% Nonidet P40 substitute/IGEPAL CA-630 (Sigma-Aldrich, I8896-50ML), 1 mM DTT (Sigma-Aldrich, 646563-10X.5 ML) and 1 U per μl RNase inhibitors (Sigma-Aldrich, 03335402001)). Nuclei were filtered and collected by centrifugation through a sucrose cushion gradient (Sigma-Aldrich, NUC201-1KT). Myelin and debris were removed and nuclei were washed (10 mM Tris-HCl, 10 mM NaCl, 3 mM MgCl_2_, 1% BSA (Miltenyi Biotec, 130-091-376), 0.1% Tween-20 (Bio-Rad, 1610781) and 1 mM DTT), pelleted and permeabilized in 0.1× lysis buffer 2 (10 mM Tris-HCl, 10 mM NaCl, 3 mM MgCl_2_, 1% BSA, 0.1% Nonidet P40 substitute/IGEPAL CA-630 and 0.01% digitonin (Invitrogen, BN20061)), washed and counted. The resulting nuclei were then prepared using the 10x Genomics single-cell Multiome ATAC + gene expression kit (10x Genomics, 1000283) and loaded on single-cell Chip J (10x Genomics, 1000234) for the recovery of 10,000 nuclei. After Tn5 transposition, single-cell isolation, barcoding and preamplification, cDNA was generated as directed in the user guide with poly(dT) primers for RT. The resulting cDNA libraries were quantified with Qubit dsDNA HS reagents (Invitrogen, Q33231) and the average fragment size was determined using high-sensitivity DNA D5000 screentape analysis (Agilent Technologies, 5067-5593 and 5067-5592).

### Targeted transcript capture for long-read RNAseq

Next, to identify the cell type(s) expressing the short intron-containing *GBA1* transcript, we performed long-read sequencing on the single-cell cDNA libraries generated from the 10x Genomics single-cell Multiome ATAC + gene expression kit. We first targeted *GBA1* in the single-cell cDNA libraries using Pacbio’s customer collaboration Iso-Seq express capture using Integrated DNA Technologies (IDT) xGEN lockdown probes protocol (https://ostr.ccr.cancer.gov/wp-content/uploads/2023/06/PacBio_TargetedIso-Seq.pdf) with primer modifications to tailor it to ONT sequencing^10^. In short, 10 ng of each single-cell cDNA library was amplified with ONT cDNA 10x primers taken from the literature^29^ and the NEBNext single-cell, low-input cDNA synthesis and amplification module (New England Biolabs, E6421S). After amplification, the cDNA was cleaned with ProNex beads (Promega, NG2001). A total of 500 ng of cDNA was then hybridized using custom *GBA1* xGen lockdown probes (IDT) (Supplementary Table 2) and washed with the xGen lockdown hybridization and wash kit (IDT, 1080577). The captured cDNA was then amplified using the same primers as above. Full details of reagents and PCR conditions are provided in Supplementary Table 3. After the *GBA1* capture, 10 ng of cDNA was taken into library preparation using an optimized version of the ONT PCS111 kit. The cDNA first underwent a biotin tagging reaction with custom oligos and PCR amplification, after which it was cleaned using AMPure XP beads (Beckman Coulter, A63881). Next, the cDNA was bound to M280 streptavidin beads (Invitrogen, 11205D) and amplified with the ONT cDNA primer. The cDNA underwent AMPure XP bead cleaning once more and was quantified using the Qubit dsDNA HS reagents. Then, 35 fmol was taken forward, the RAP T adaptor was added and the sample underwent standard loading on an R.9.4.1 flow cell on a PromethION with Minknow 22.10.7 software.

### Whole-genome DNA long-read sequencing analysis

All DNA sequencing runs were basecalled on the National Institutes of Health (NIH) high-performance computing (HPC) cluster (Biowulf) using Guppy (version 6.1.2) in super-accuracy mode with the ‘dna_r9.4.1_450bps_modbases_5mc_cg_sup_prom.cfg’ configuration file. Basecalled files were mapped to hg38 using Minimap2 (version 2.24/2.26)^30^ preserving methylation tags (‘samtools fastq -TMm,Ml ${FASTQ_PATH}/${BAM_FILE} | minimap2 -y -x map-ont -t 20 -a --eqx -k 17 -K 10 g’). SNVs were called using Clair3 (version 1.0.4)^31^ and structural variants were called using Sniffles (version 2.2)^32^.

### Untargeted long-read RNAseq analysis

All RNAseq runs were basecalled on the NIH HPC cluster (Biowulf) using Guppy (version 6.1.2) in super-accuracy mode with the ‘dna_r9.4.1_450bps_sup_prom.cfg’ configuration file for cDNA.

Pychopper version 2.7.1 (https://github.com/epi2me-labs/pychopper) was run on the cDNA FASTQ files with a minimum mean read quality of 7.0 and a minimum segment length of 50 for kit SQK-PCS111. Reads passing quality control were mapped to hg38 using Minimap2 (version 2.26)^30^ with splice-aware parameters (‘-t 10 -ax splice -k14 -uf’). Only reads with a minimum mapping quality of 40 and flagged as primary alignment were kept. For transcript calling and quantification, we used StringTie2 (version 2.2.1)^33^. StringTie2 was first run in long-read reference free mode (‘stringtie -L -R -m 50’) to capture our unannotated intronic region. Then, we generated a reference GTF file with the canonical *GBA1* transcript (ENST00000368373.8) and the transcript containing part of intron 8 and reran our samples against this reference (‘stringtie --rf -G ${reference_annotation} -L -v -p 10’) for quantification of the intron-containing transcript and canonical transcript. Each mapped BAM and StringTie2 annotation was then manually inspected on Integrative Genomics Viewer (IGV; version 2.16.0)^34^.

Additionally, we calculated regional sequencing depth for different *GBA1* regions. For this, we used SAMtools (version 1.17)^35^ to subset the *GBA1* regions from each hg38-mapped BAM and used ‘samtools coverage -q5 -Q20 --ff UNMAP,SECONDARY,QCFAIL,DUP -r $chr:$start-$end ${IN}’ to calculate the mean depth at each region of interest. These regions of interest included (1) exons 8 and 9; (2) intron 8; (3) the ~40-bp short transcript region in intron 8; (4) intron 8 minus the ~40-bp short intron 8 transcript region; and (5) *GBA1* and *GBA1LP*. These coordinates were based on ENST00000368373.8 for *GBA1* and the exact BED file can be found in Supplementary Table 4. To normalize coverage metrics across samples, we extracted the number of uniquely mapped reads per sample over the whole genome using ‘samtools view -c -F 260 ${IN}’ and divided these reads by a million. We then divided our mean depth metric by the number of reads for a normalized coverage per million metric. Normalized coverage counts were averaged across rs3115534 genotypes and plotted using ggplot2 (ref. ^36^) in R (version 4.3.0).

Lastly, to check that these reads mapped uniquely to *GBA1* and not to *GBA1LP*, we manually edited the rs3115534 variant in the FASTQ data and looked for differences in mapping. To do this, we subsetted the *GBA1* and *GBA1LP* regions for one GG and one TT from the hg38-aligned BAM files and converted the subsets back to a FASTQ using SAMtools (version1.17) bam2fq. Then, using Virtual Studio Code (https://github.com/microsoft/vscode), we manually changed the rs3115534 variant from a G to a T and vice versa and remapped the edited FASTQ. Mapped BAM files were then inspected on IGV 2.16.0.

### Transcript capture long-read RNAseq analysis

Long-read single-nucleus RNAseq data were basecalled using Guppy (version 6.1.2) with the ‘dna_r9.4.1_450bps_sup_prom.cfg’ configuration file. The run FASTQ was then mapped using Minimap2 (version 2.26) with splice-aware parameters to hg38, subset for *GBA1* ± 1 Mb and then the subset region reverted to a FASTQ using SAMtools (version 1.17) bam2fq. This subset FASTQ was then split into a FASTQ per cell type using corresponding Illumina unique cell type barcodes. Each barcode was matched against the FASTQ allowing for one mismatch and the resulting FASTQs were sorted to only keep unique read ids. Each cell FASTQ was quality-controlled using Pychopper (version 2.7.1) with standard parameters and mapped using Minimap2 (version 2.26) with splice-aware parameters. Only reads with a minimum mapping quality of 40 and flagged as primary alignment were kept. Then, we performed the same depth calculations as above using SAMtools (version 1.17) and used the number of cells in the original 10x library as the normalization factor for our depth per region across cell types. Regional *GBA1* and transcript coverage was plotted using ggplot2. As an additional quality control, we checked barcode sequence diversity in each of our cell types by calculating the ratio of barcodes in our data divided by total Illumina barcodes. Density plots of barcode usage were generated with ggplot2.

### Short-read sequencing data generation and processing

Illumina short-read data were generated for the same LCLs to complement the ONT data. RNA was extracted using the same method described above and library preparation and sequencing were completed by Psomagen (https://www.psomagen.com/). The ribosomal RNA removed total was fragmented and primed for cDNA synthesis using TruSeq stranded total RNA library prep kit reagents (96 samples; Illumina, 20020597) before incubating for 8 min at 95 °C (C1000 Touch Thermal Cycler). The cleaved and primed RNA was reverse-transcribed into first-strand cDNA using SuperScript II reverse transcriptase (Thermo Fisher Scientific, 18064-014). Actinomycin D and first-strand synthesis act D mix were added to enhance strand specificity. The second strand was synthesized using the second-strand master mix from the same TruSeq stranded total RNA kit (16 °C incubation for 1 h). To enable adaptor ligation, the dscDNA was adenylated at the 3′ end and RNA adaptors were subsequently ligated to the dA-tailed dscDNA. Finally, additional amplification steps were carried out to enrich the library material. The final library was validated (D1000 ScreenTape System) and quantified (Quant-iT PicoGreen dsDNA assay kit).

The sequencing library was then loaded onto a flow cell containing surface-bound oligos complementary to the adaptors in the library and amplified into distinct clusters. Following cluster generation, the Illumina sequencing by synthesis (SBS) technology (Illumina Novaseq 1.5 5000/6000 S4 reagent kit, 300 cycles, 20028312; Illumina Novaseq 1.5 Xp 4-lane kit, 20043131) was used to accurately sequence each base pair. Real-time analysis software (RTA version 3) was used to basecall data from raw images generated by the Illumina SBS technology. The binary BCL/cBCL files were then converted to FASTQ files using bcl2fastq (bcl2fastq version 2.20.0.422), a package provided by Illumina. Illumina FASTQ data were aligned to hg38 using STAR (version 2.7.10)^37^. We then calculated regional depth following the same steps as for the bulk RNA ONT long-read data.

### Accessing publicly available whole-genome sequencing (WGS) and RNAseq

We additionally looked into Illumina transcriptomic data from the AMP PD (https://www.amp-pd.org/) and 1000 Genomes Project (https://www.internationalgenome.org/) datasets for homozygous reference, heterozygous and alternative allele carriers of the rs3115534 variant. For AMP PD, we extracted data for 1 homozygous G carrier, 47 heterozygous G carriers and 98 non-European controls. The ancestry of extracted data was divided as follows: 12 African admixed (7 GT and 5 TT), 15 African (1 GG, 5 GT and 9 TT), 118 European (35 GT and 83 TT) and 1 Asian (1 TT). Details on AMP PD samples are provided in Supplementary Table 5. Full details on data generation and processing of WGS and RNAseq data were provided in previous studies^38,39^. Full details on ancestry predictions were provided in a previous study^11^. Using these files, we calculated regional depth following the same steps as for the bulk RNA ONT long-read data.

The 1000 Genomes Project WGS and RNAseq data were downloaded and samples were demultiplexed to individual sample FASTQ files and aligned to hg38 using STAR version (version 2.6.1)^37^. We extracted data for 7 homozygous G carriers, 40 heterozygous G carriers and 41 homozygous T carriers, all of African ancestry. Details on 1000 Genomes Project samples are provided in Supplementary Table 6. We calculated regional depth following the same steps as for the bulk RNA ONT long-read data. Additionally, we accessed African American ancestry Illumina RNAseq data from the HBCC (*n* = 92). Within the 92 samples, there were 6 GG, 20 GT and 66 TT samples. These data were accessed through the National Institute of Mental Health (NIMH) Data Archive (https://nda.nih.gov/). FASTQ files were aligned to hg38 using STAR version (version 2.6.1)^37^. Details on Illumina HBCC samples are provided in Supplementary Table 7. The tissue used in this research was obtained from the HBCC Intramural Research Program (IRP; http://www.nimh.nih.gov/hbcc).

To calculate the importance of the rs3115534-G allele with respect to the depth per region, we ran linear regressions in R (version 4.3.0) with genotypes as the predictor and normalized depth as the outcome for each dataset and region. Because of the low numbers of the GG groups, we combined the GG and GT genotypes for the regression in each dataset (Illumina Coriell, ONT Coriell, ONT HBCC, ONT CRISPR and AMP PD) except for the 1000 Genomes Project and Illumina HBCC datasets, where there were >5 samples with a GG genotype. For the 1000 Genomes Project and Illumina HBCC datasets, we ran the regression with GG, GT and TT split into three groups.

### Validation of the intronic expression *GBA1* transcript

To validate the presence of the potential *GBA1* intron-containing transcript we generated custom primers that bind to the most highly expressed part of the transcript. The forward (GBA1_X11_F9, 5′-GCGACGCCACAGGTAG-3′) and reverse (GBA1_X11_R9, 5′-CTTTGTCCTTACCCTAGAACCTC-3′) primers specifically designed to start before exon 9 and end at exon 11 of *GBA1* were used at a final concentration of 0.8 µM (IDT); an additional reverse primer specific to the 3′ untranslated region of *GBA1* (GBA1_UTR_R4, 5′-CCTTTGTCCTTACCCTAGAACC-3′) was also used in conjunction with GBA1_X11_F9 (Supplementary Table 8). As input, we used RNA from LCLs with and without the rs3115534-G allele. RNA was quantified using Qubit RNA HS assay (Invitrogen, Q32852) and reverse-transcribed to cDNA. cDNA was synthesized from RNA using a high-capacity cDNA RT kit (Thermo Fisher Scientific, 4368814) following the manufacturer’s recommendations. The cDNA then underwent PCR using REDTaq ReadyMix (Millipore Sigma, R2523-20RXN). PCR products were mixed with 6× loading dye (New England Biolabs, B7024S), loaded onto a 1% agarose gel containing SYBR safe DNA gel stain (Thermo Fisher Scientific, S33102), sized with a 1 kb plus DNA ladder (New England Biolabs, N3200L) and imaged on a ChemiDoc Imaging System (Bio-Rad, 12003153). PCR bands were excised from 1% agarose gel and DNA was purified using the NucleoSpin gel and PCR cleanup kit (Takara Bio, 740609) according to the manufacturer’s instructions. Sanger sequencing was performed by Psomagen using conventional protocols (Supplementary Table 9).

In addition, we aimed to assess whether the short *GBA1* transcript is capped. To check the presence of a 5′ cap, we selected three Coriell lines (ND01137-GG, ND02892-GT and ND22789-TT) for CAGE library prep and sequencing performed by DNAFORM (https://www.dnaform.jp/en/). In brief, RNA quality was assessed with a Bioanalyzer (Agilent) and all samples had an RIN above 8.3. First-strand cDNAs were transcribed to the 5′ ends of capped RNAs and attached to CAGE ‘barcode’ tags. The samples were then sequenced on an Illumina NextSeq 500 and the sequenced CAGE tags were mapped to the human hg38 genome using BWA software (version 0.5.9). Mapped BAM files were inspected for transcription start site clusters using IGV (version 2.16.0).

### CRISPR editing of rs3115534

To determine whether rs3115534 is the functional variant in this GWAS locus, CRISPR editing was performed by Synthego (https://www.synthego.com/). CRISPR editing was performed using two LCLs (ND01137-GG and ND22789-TT) with the aim to edit both LCLs to the opposite genotype (Supplementary Table 10). In brief, cell pools were created using high-quality chemically modified synthetic single guide RNA (sgRNA) and SpCas9 transfected as RNPs to ensure high editing efficiencies without the use of any selection markers that could negatively affect cell biology. Knock-ins were generated using either single-stranded DNA or plasmid, depending on the insert size. The parental cells were electroporated with SpCas9 and target-specific sgRNA to generate the edited cell pool. Similarly, mock-transfected cell pools were made by electroporating the parental cells with SpCas9 only and confirmed to be unedited at target locus. After editing, cells (mock-transfected pools, intermediate pools and final fully edited LCLs) were processed for subsequent assays. A predesigned genotyping assay specific to rs3115534 (Thermo Fisher Scientific, C__57592022_20) was used to confirm the CRISPR-edited genotypes using an allelic discrimination qPCR assay (QuantStudio 6 Pro, Applied Biosystems, A43159). Additionally, the CRISPR lines were manually inspected after long-read sequencing on IGV (v2.16.0) to confirm no accidental editing of *GBA1LP*.

### Bioinformatic annotation of rs3115534

To assess the potential functional effect of rs3115534, we investigated several annotation resources. Summary statistics from the largest African PD GWAS were used to generate a LocusZoom plot using African linkage disequilibrium patterns^11,40^. RegulomeDB (version 2.2) was explored for rs3115534 to assess its effect on motifs and genome accessibility^41^. The UCSC Genome Browser was accessed to investigate the conservation of this allele across vertebrates. To evaluate whether rs3115534 was involved in splicing, we assessed the following algorithms: AGAIN, SpliceAI and Branchpointer^42–44^. Branchpointer was used in R (version 4.3.0) to evaluate the impact of rs3115534 on branchpoint architecture. We ran rs3115534 as the query file and calculated branchpoint predictions using the ‘queryType = SNP’ option. Gencode’s hg38 version 44 release was used as our reference file. Branchpoint predictions were plotted through Branchpointer’s plotBranchpointWindow script. In addition, we assessed all coding and noncoding *GBA1* variants (*n* = 1,625) present in gnomAD (version 4) for their potential to disrupt intronic branchpoint sequences using AGAIN. To identify functional evidence for branchpoint usage, 293Flp-in cells (Thermo Fisher Scientific, R78007) were genotyped by aligning 100-nt paired-end RNAseq reads from chromatin-associated RNA (Damianov et al., unpublished) to *GBA1* exon 9 and the flanking upstream region. The alignment confirmed that these cells are homozygous for rs3115534-T. Similar sequence analysis indicated the presence of rs2990223-G variation in all reads mapping to the highly homologous *GBA1LP* and these reads were kept separate. Branch site reads from 293Flp-in cells, obtained by RBM5 and SF3A3 RNPseq or RBM10 and SF3A3 IPseq, were then aligned to *GBA1* and *GBA1LP*. Reads that could originate from either of these two genes were kept and analyzed separately. Branchpoint prediction was performed as described previously^14^.

### Assessing the protein-coding ability of short transcript

#### Protein extraction

LCLs from Coriell Biorepository were maintained in suspension with RPMI-1640 medium (Thermo Fisher Scientific, 11875093) containing 2 mmol L^−1^ GlutaMAX (Thermo Fisher Scientific, 35050061) and 15% FBS (Thermo Fisher Scientific, A5256701) at 37 °C in 5% carbon dioxide. Protein was extracted from LCLs (5 × 10^6^ cells) using a Tris-HCl cell lysis buffer (Cell Signaling Technology, 9803) containing a protease and phosphatase inhibitor cocktail (Cell Signaling Technology, 5872) on ice.

#### Multiplex western blotting

Protein was normalized to 30 μg and loaded into a 4–20% precast polyacrylamide gel (Bio-Rad, 4561094) before being transferred to a nitrocellulose membrane (Bio-Rad, 1704270). The membrane was blocked for 1 h (LiCor Biosciences, 927-60001) and incubated (4 °C) with primary anti-GCase (1 μg ml^−1^ working concentration, 1:1,000 dilution; Sigma-Aldrich, polyclonal clone G4171, RRID: AB_1078958) and anti-β-actin (1 μg ml^−1^ working concentration, 1:1,000 dilution; Abcam, monoclonal clone mAbcam 8224, RRID: AB_449644) antibodies on a shaker overnight. Finally, the membrane was incubated with donkey anti-rabbit (LiCor Biosciences, lot D30328-05, 926-68073, RRID: AB_10954442) and donkey anti-mouse (LiCor Biosciences, lot D30124-05, 926-32212, RRID: AB_621847) secondary antibodies (1:20,000 dilution for both secondary antibodies) at room temperature for 1 h before imaging (Odyssey DLx, LiCor Biosciences). The entire procedure was repeated with identical results.

#### ECL western blotting

Protein was normalized to 30 μg and loaded into a 4–20% precast polyacrylamide gel (Bio-Rad, 4561094) before being transferred to a nitrocellulose membrane (Bio-Rad, 1704270). The membrane was blocked for 1 h with 5% blotting-grade blocker (Bio-Rad, 1706404) and incubated (4 °C) with primary anti-GCase (1 μg ml^−1^ working concentration, 1:1,000 dilution; Sigma-Aldrich, polyclonal clone G4171, RRID: AB_1078958) antibody on a shaker overnight in 5% blotting-grade blocker. After washing with TBS-T (0.1% Tween-20), the membrane was incubated with goat anti-rabbit IgG (H + L) cross-adsorbed secondary antibody conjugated to horseradish peroxidase (HRP; 1:1,000 dilution; Invitrogen, 31462, RRID: AB_228338) for 1 h. Following washing with TBS-T, the membrane was incubated with anti-β-actin antibody (1 μg ml^−1^ working concentration, 1:1,000 dilution; Abcam, monoclonal clone mAbcam 8224, RRID: AB_449644) for 1 h and then probed with goat anti-mouse IgG (H + L) cross-adsorbed secondary antibody conjugated to HRP (1:1,000 dilution; Invitrogen, 31432, RRID: AB_228302). The membrane was then probed with Clarity Max Western ECL substrate for 1 h (Bio-Rad, 1705062). The blot was imaged on the ChemiDoc MP Imaging System (Bio-Rad, 12003153).

#### Mass spectrometry analysis of western blot

Protein extraction, normalization and gel electrophoresis were performed as detailed above. The bands were visualized with Coomassie blue stain (Bio-Rad, 1610786) and manually excised. Gel bands between 2 and 15 kDa were excised, reduced with 5 mM TCEP (Sigma-Aldrich, 580560) and alkylated with 5 mM *N*-ethylmaleimide (Sigma-Aldrich, 04259). Samples were digested with trypsin (Promega, V5280) at a 1:20 (w/w) ratio of trypsin to sample at 37 °C for 18 h. Peptides were extracted then desalted using Oasis HLB plate (Waters, WAT058951). Liquid chromatography–tandem mass spectrometry data acquisition was performed on an Orbitrap Lumos mass spectrometer (Thermo Fisher Scientific) coupled to an Ultimate 3000 high-performance liquid chromatograph. Peptides were separated on a ES902 Easy-Spray column (75-μm inner diameter, 25-cm length, 3-μm C18 beads; Thermo Fisher Scientific). Mobile phase B was increased from 3% to 20% in 39 min. Lumos was operated in data-dependent mode. Peptides were fragmented with a higher-energy collisional dissociation method at a fixed collision energy of 35. The Proteome Discoverer 2.4 software was used for database search using the Mascot search engine. Data were searched against the SWISS-PROT human database.

#### Assessing GBA1 protein expression in UK Biobank

To replicate previous pQTL results of rs3115534, we accessed the UK Biobank 500K whole-genome sequencing data through the UK Biobank Research Analysis Platform (https://ukbiobank.dnanexus.com/). We used the population-level variant data produced using Illumina DRAGEN version 3.7.8. Genotypes for rs3114435 were extracted using Plink (version 2.0; https://www.cog-genomics.org/plink/2.0/) and only those individuals with African or African admixed ancestry were kept. We then merged the genotype information with available Olink proteomic measures to get the GBA1 protein level per individual. Any unavailable data from the protein information were dropped. We then generated a violin plot of GBA1 protein expression per genotype using ggplot2 (ref. ^26^) in R (version 4.3.0). We ran a linear regression in R (version4.3.0) with genotypes as the predictor and GBA1 protein levels as the outcome to test for significance.

### Assessing GCase activity across *GBA1* genotypes

GCase activity was assessed across *GBA1* genotypes in 710 samples containing 97 non-PD (used as controls), 122 idiopathic PD without a known *GBA1* mutation, 95 *LRRK2* p.G2019S carriers, 99 rs3115534-GT, 9 rs3115534-GG and 99 PD risk variant carriers (p.E365K or p.T408M), 90 *GBA1* p.N409S (mild) and 99 *GBA1* severe mutations (for example, *GBA1* p.L483P). Exonic *GBA1* mutations are written on the basis of current recommendations, which include the 39-aa signal peptide at the start of the protein (for example, p.E365K = p.E326K; p.T408M = p.TM; p.N409S = p.N370S; p.L483P = p.L444P). The GCase enzyme was extracted from one dried blood spot punch (Ø 3.2 mm) per sample and measured by incubating for 1 h at 37 °C under agitation with an aqueous buffer containing citrate, phosphate, taurocholic acid sodium salt, NaN_3_ and Triton X-100 (pH 5.2). Next, an aqueous solution with the synthetic substrate 4-methylumbelliferyl β-d-glucopyranoside and NaN_3_ was added followed by a second incubation step for 16 h at 37 °C under agitation. The enzymatic reaction was quenched by addition of stop buffer (aqueous glycine solution, pH 10.5 adjusted by NaOH). The enzymatic product, 4-methylumbelliferone was quantified by fluorimetry on a microplate reader (Victor X2, PerkinElmer). The instrument was calibrated using an external calibration curve. The enzymatic activity is specified in units of μmol L^−1^ h^−1^ (amount of product per blood volume per incubation time). Each sample was measured in duplicate and a third replicate was used for background correction. The background of the chemical blank was determined by the addition of stop buffer before the substrate. As quality parameters for the assay, standard blood samples were added to each batch to ensure the accuracy of the determination. To analyze the data, we ran a linear regression in R (version 4.3.0) with genotypes as the predictor and GCase activity levels as the outcome to test for significance. We also compared each group to each other with a one-sided *P* value test in R.

### Reporting summary

Further information on research design is available in the Nature Portfolio Reporting Summary linked to this article.

## Online content

Any methods, additional references, Nature Portfolio reporting summaries, source data, extended data, supplementary information, acknowledgements, peer review information; details of author contributions and competing interests; and statements of data and code availability are available at 10.1038/s41594-024-01423-2.

## Supplementary information

Supplementary Information Supplementary Figs. 1–23, Tables 1–15 and source data for Supplementary Figs. 10, 11 and 13.

Reporting Summary

Supplementary Table 1 Supplementary Table 13: Mass spectrometry analysis of excised 4–20% agarose gel region predicted to contain truncated GBA1 protein (too large to be part of the joint Supplementary Information file).

Supplementary Table 2 Full list of the GP2 consortium members.

## Data Availability

Unedited Coriell LCL lines are available online (https://www.coriell.org/). CRISPR-edited Coriell LCL lines are available upon request and the establishment of a material transfer agreement (MTA) with Coriell and NIH and CARD abiding by the Coriell NINDS Human Genetics Repository MTA for biospecimens. All generated LCL Coriell ONT DNAseq, CAGEseq and RNAseq data (Illumina and ONT) are available online (https://www.amp-pd.org/) through GP2 tier 2 access, which is obtainable by filling in the form (https://www.amp-pd.org/researchers/data-use-agreement). It is part of GP2 release 7 (10.5281/zenodo.10962119; https://console.cloud.google.com/storage/browser/gp2tier2/release7_30042024/gp2_omics/Alvarez_Jerez_et_al_2024; data path: gp2tier2/release7_30042024/gp2_omics/Alvarez_Jerez_et_al_2024). AMP PD Illumina blood-based RNAseq data are available online (https://www.amp-pd.org/) after signing the data use agreement. The 1000 Genomes Project data are publicly available online (https://www.internationalgenome.org/). Brain tissue bulk RNAseq data (https://www.ncbi.nlm.nih.gov/projects/gap/cgi-bin/study.cgi?study_id=phs000979.v3.p2) and frontal cortex data (https://nda.nih.gov/edit_collection.html?id=3151) are available online. Summary statistics for *cis*-eQTLs and a catalog of ancestry-specific eQTLs were obtained from Kachuri et al.^12^ (10.5281/zenodo.7735723).

## Code availability

All scripts and code for this project, including all the identifiers and version numbers for software used, can be found on GitHub (https://github.com/GP2code/GBA1-rs3115534-branchpoint) and Zenodo (10.5281/zenodo.10484208) (ref. ^45^).
