# Supplementary material for: African ancestry neurodegeneration risk variant disrupts an intronic branchpoint in GBA1
Source: Nat Struct Mol Biol. 2024 Dec 12;31(12):1955–63. doi: 10.1038/s41594-024-01423-2 (PMC11638064; doi:10.1038/s41594-024-01423-2)
Supplement: Supplementary file 1 — Supplementary Figs. 1–23, Tables 1–15 and source data for Supplementary Figs. 10, 11 and 13. [file 41594_2024_1423_MOESM1_ESM.pdf]

nature structural & molecular biology

Supplementary information https://doi.org/10.1038/s41594-024-01423-2

African ancestry neurodegeneration risk variant disrupts an intronic branchpoint in *GBA1*

In the format provided by the authors and unedited

Table of Contents

Figures

Supplementary Figure 1: rs3115534 lowers total *GBA1* protein expression in the UK Biobank dataset (n=1147).

Supplementary Figure 2: Stringtie2 isoforms from GG sample.

Supplementary Figure 3: Additional coverage plots for lymphoblastoid cell lines sequenced with Oxford Nanopore Technologies long-read RNA sequencing (n=8).

Supplementary Figure 4: Integrative Genome Browser inspection of Oxford Nanopore Technologies long-read RNA sequencing mapped reads in lymphoblastoid cell line ND01137 rs3115534-GG bam and *in silico* edited to ND01137 rs3115534-TT bam.

Supplementary Figure 5: Regional coverage plots from human frontal cortex Oxford Nanopore Technologies long-read RNA sequencing showing enrichment of intron 8 expression in rs3115534-G carriers (n=8).

Supplementary Figure 6: Regional coverage plots from lymphoblastoid cell lines sequenced with Illumina RNA sequencing showing enrichment of intron 8 expression in rs3115534-G carriers (n=18).

Supplementary Figure 7: Regional coverage plots from human frontal cortex Illumina short-read RNA sequencing showing enrichment of intron 8 expression in rs3115534-G carriers (n=92).

Supplementary Figure 8: Regional coverage plots from 1000 Genomes dataset showing enrichment of intron 8 expression in rs3115534-G carriers (n=88).

Supplementary Figure 9: Regional coverage plots from AMP-PD blood-based RNA sequencing showing enrichment of intron 8 expression in rs3115534-G carriers (n=146).

Supplementary Figure 10: Reverse transcription PCR of RNA from lymphoblastoid cell lines of various genotypes (GG, GT, or TT).

Supplementary Figure 11: Reverse transcription PCR products used for Sanger sequencin**g.**

Supplementary Figure 12: CAGEseq sequencing rs3115534-GG, GT, TT to assess transcription start sites across genotypes.

Supplementary Figure 13: Western blot of various lymphoblastoid cell lines rs3115534 genotypes to assess protein coding ability.

Supplementary Figure 14: Additional coverage plots for CRISPR-edited lymphoblastoid cell lines sequenced with Oxford Nanopore Technologies long read RNA sequencing (n=7).

Supplementary Figure 15: Low *GBA1* gene expression across major brain cell types using single nuclei RNA sequencing.

Supplementary Figure 16: Enriching of *GBA1* in single nuclei RNA sequencing shows *GBA1* expression across major brain cell types.

Supplementary Figure 17: Assessment of conservation across species for rs3115534.

Supplementary Figure 18: Methylation differences in rs3115534 across CRISPR-edited lymphoblastoid cell lines.

Supplementary Figure 19: Methylation differences in rs3115534 across lymphoblastoid cell lines.

Supplementary Figure 20: Methylation differences in rs3115534 across frontal cortex samples.

Supplementary Figure 21: rs3115534-T in human 293Flp-in cells is engaged as a branchpoint of *GBA1* intron 8.

Supplementary Figure 22: Branchpoint investigation of rs140335079 and rs745734072.

Supplementary Figure 23: No expression differences identified due to rs140335079.

Tables

Supplementary Table 1: Biosamples used for assessment of effects of *GBA1* rs3115534.

Supplementary Table 2: Probes used to capture *GBA1* transcripts.

Supplementary Table 3: 10x ONT capture methods.

Supplementary Table 4: Bed file for coverage plots of *GBA1*.

Supplementary Table 5: AMP-PD samples used for coverage information.

Supplementary Table 6: 1000 Genomes samples used for coverage information.

Supplementary Table 7: HBCC Illumina RNAseq samples used for coverage information.

Supplementary Table 8: Reverse transcription PCR Primers and Conditions.

Supplementary Table 9: Sanger sequencing of excised PCR bands.

Supplementary Table 10: CRISPR edited LCLs for rs3115534.

Supplementary Table 11: Overview of statistical tests performed for coverage.

Supplementary Table 12: Transcript quantifications using Stringtie2.

Supplementary Table 13: Mass spectrometry analysis of excised 4-20% agarose gel region predicted to contain truncated *GBA1* protein.

Supplementary Table 14: Reads mapping to *GBA1* after 10x capture enrichment.

Supplementary Table 15: *GBA1* variants affecting branchpoint sequences according to AGAIN algorithm.

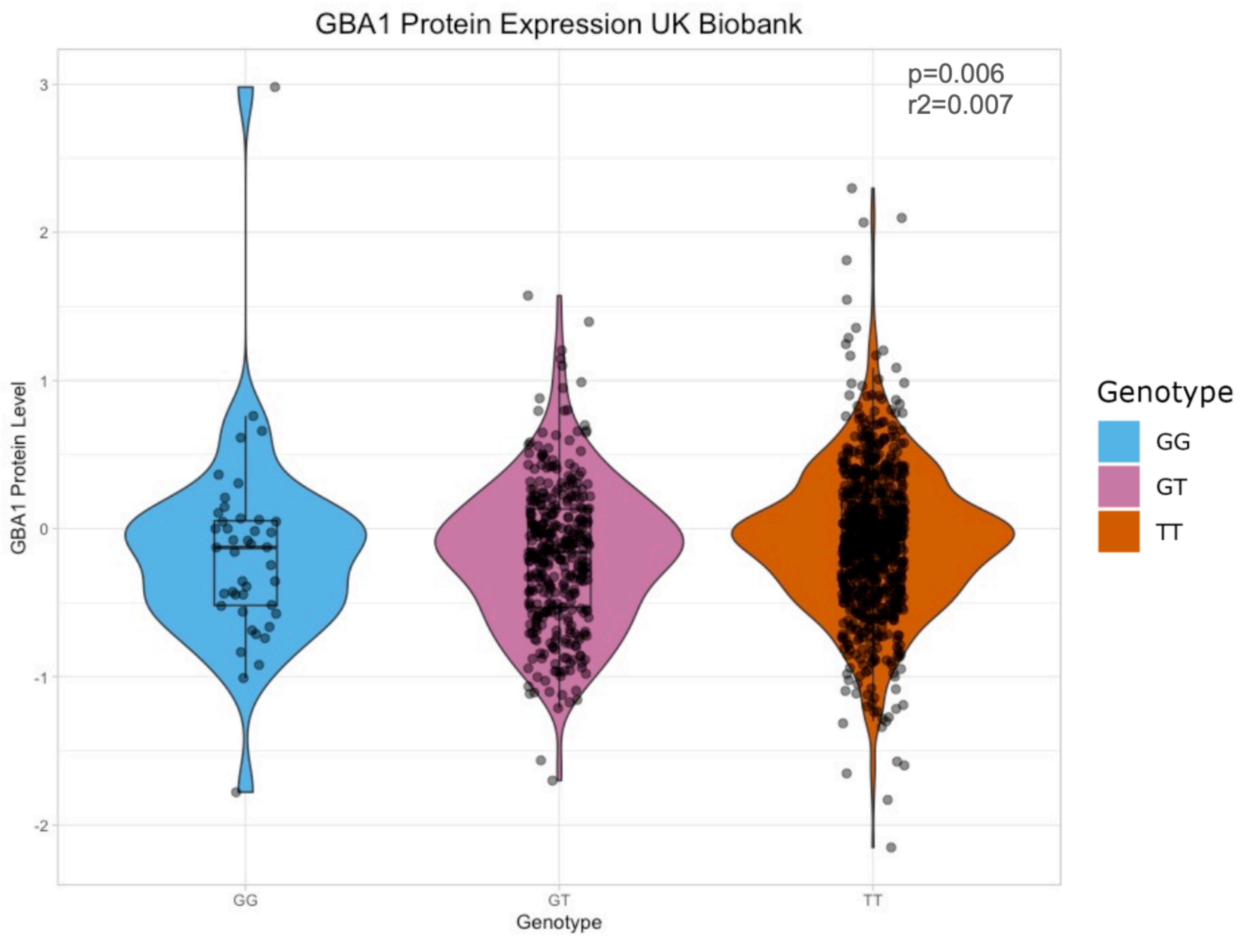

Supplementary Figure 1: rs3115534 lowers total *GBA1* protein expression in the UK Biobank dataset (n=1147). rs3115534-G significantly lowers total *GBA1* protein expression ($p < 0.05$) in the UK Biobank dataset. Only African and African-Admixed individuals were kept for this pQTL analysis. This replicates the previously reported pQTL of rs3115534 in the African American population (see Figure 1). The observed decrease in protein levels is further supported by reduced GCase activity levels. A linear regression was run with GG/GT/TT in separate groups. For all boxplots, the center line represents the median, edges of box represent Q1 and Q3, and ends of bars represent the maximum and minimum not including outliers.

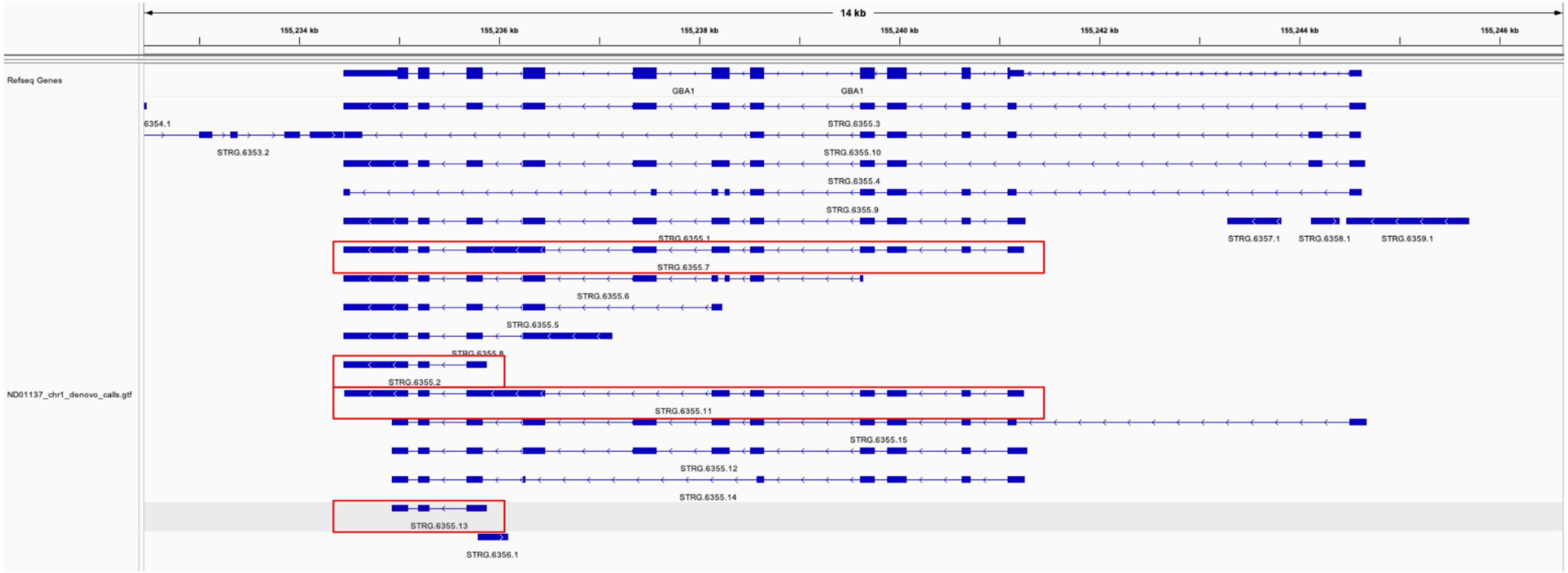

Supplementary Figure 2: Stringtie2 isoforms from GG sample. Stringtie2 was run *de novo* in long-read mode for ND01137, a rs3115534-GG lymphoblastoid cell line sequenced with long-read ONT RNA sequencing. STRG.6355.2, STRG.6355.7, STRG.6355.11, and STRG.6355.13 in the red boxes show abnormal intron 8 splicing.

a)

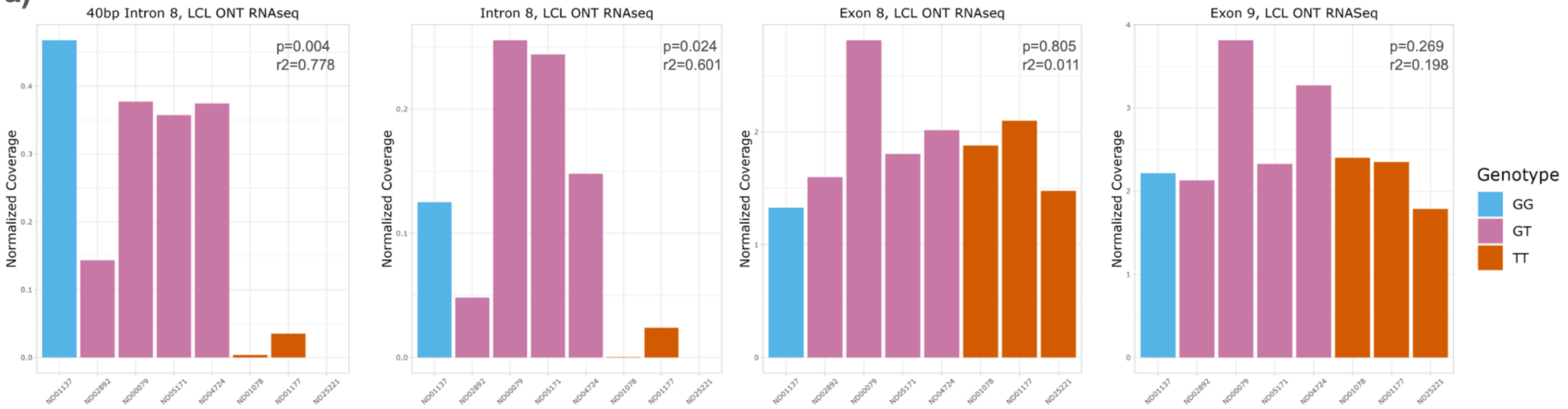
40bp Intron 8, LCL ONT RNAseq
p=0.004
r2=0.778
Intron 8, LCL ONT RNAseq
p=0.024
r2=0.601
Exon 8, LCL ONT RNAseq
p=0.805
r2=0.011
Exon 9, LCL ONT RNASeq
p=0.269
r2=0.198
Normalized Coverage
Genotype
GG
GT
TT

b)

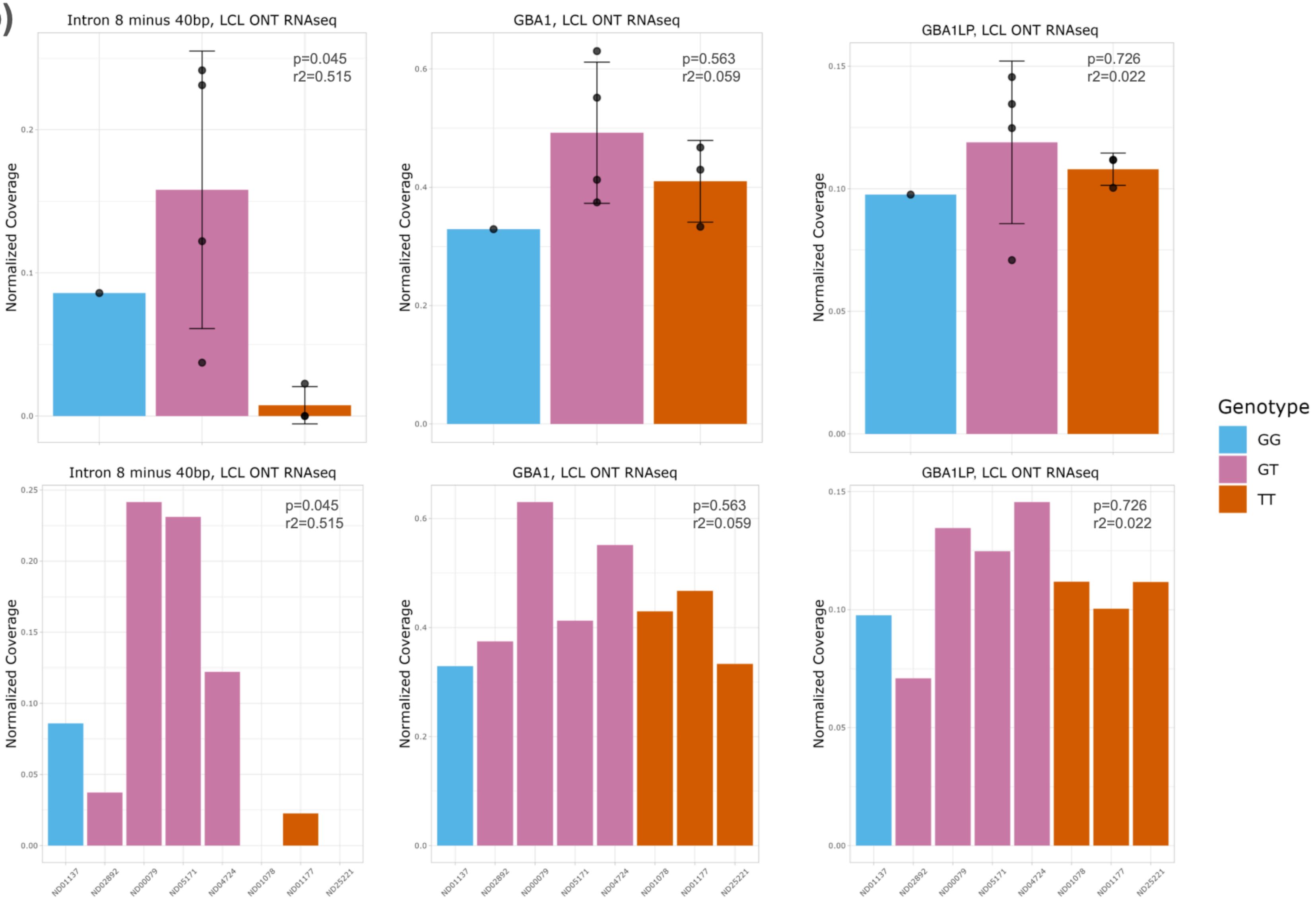

Intron 8 minus 40bp, LCL ONT RNAseq
p=0.045
r2=0.515
GBA1, LCL ONT RNAseq
p=0.563
r2=0.059
GBA1LP, LCL ONT RNAseq
p=0.726
r2=0.022
Normalized Coverage
Genotype
GG
GT
TT
ND01137
ND02892
ND00079
ND05171
ND04724
ND01078
ND01177
ND25221

Supplementary Figure 3: Additional coverage plots for lymphoblastoid cell lines sequenced with Oxford Nanopore Technologies long-read RNA sequencing (n=8). a) Per sample quantification of intron 8 expression matching Figure 2 collapsed per genotype plots including 40bp intron 8 region prior to exon 9, full intron 8, exon8 only and exon 9 only. Coverage for all panels normalized by dividing mean depth by total number of mapped reads per million as detailed in methods. b) Additional coverage plots from LCLs for intron 8 minus 40bp transcript region, *GBA1*, and *GBA1LP*. Plot are shown both per sample and collapsed by genotype. No significant differences shown in G allele carriers *GBA1* and *GBA1LP* gene expression. For all panels, a linear regression was run with GG + GT in one group versus TT. Error bars represent standard deviation for all panels with the center at the mean.

a)

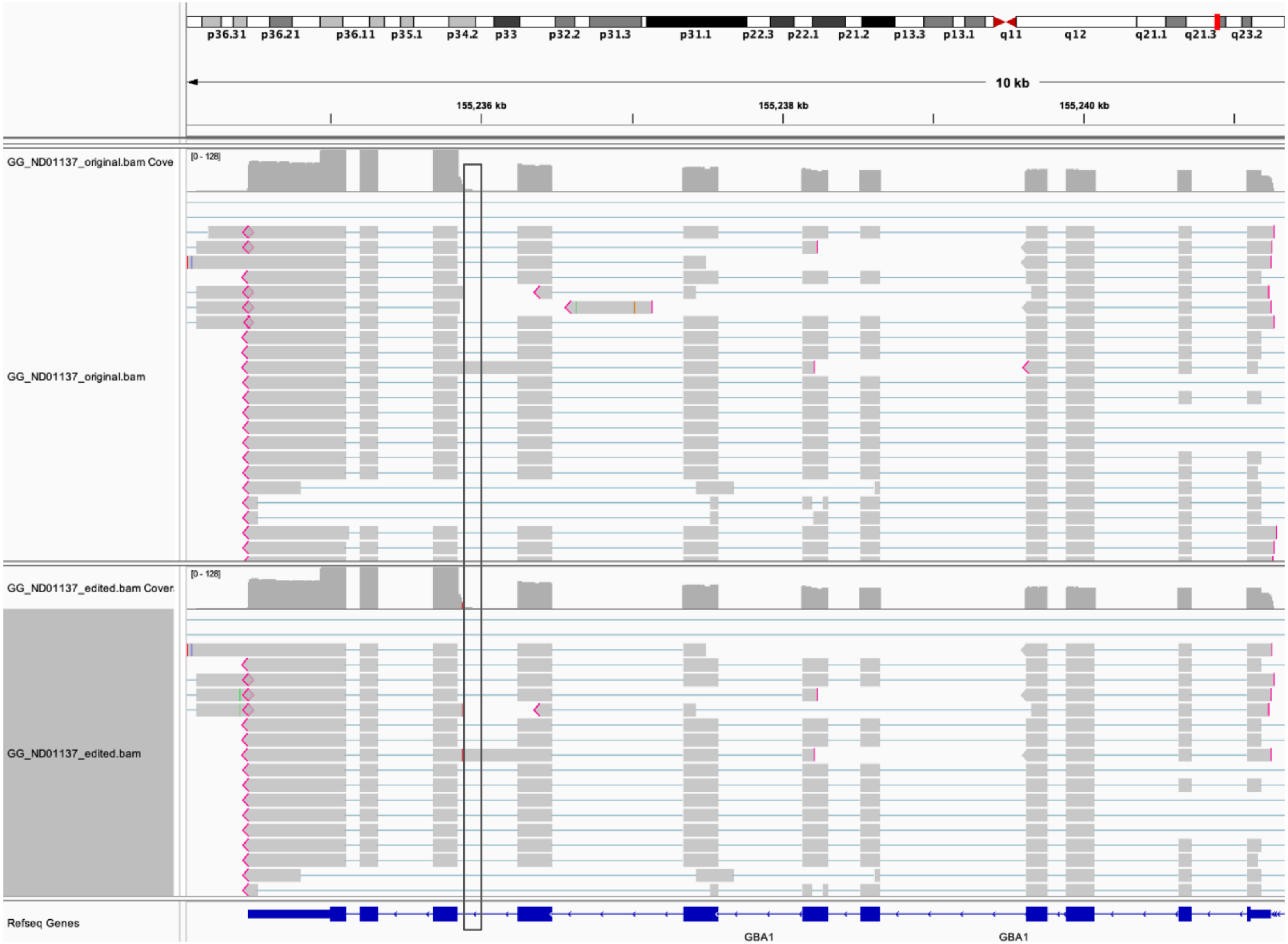
p36.31
p36.21
p36.11
p35.1
p34.2
p33
p32.2
p31.3
p31.1
p22.3
p22.1
p21.2
p13.3
p13.1
q11
q12
q21.1
q21.3
q23.2
10 kb
155,236 kb
155,238 kb
155,240 kb
GG_ND01137_original.bam Cove
[0 - 128]
GG_ND01137_original.bam
GG_ND01137_edited.bam Cover
[0 - 128]
GG_ND01137_edited.bam
Refseq Genes
GBA1
GBA1

b)

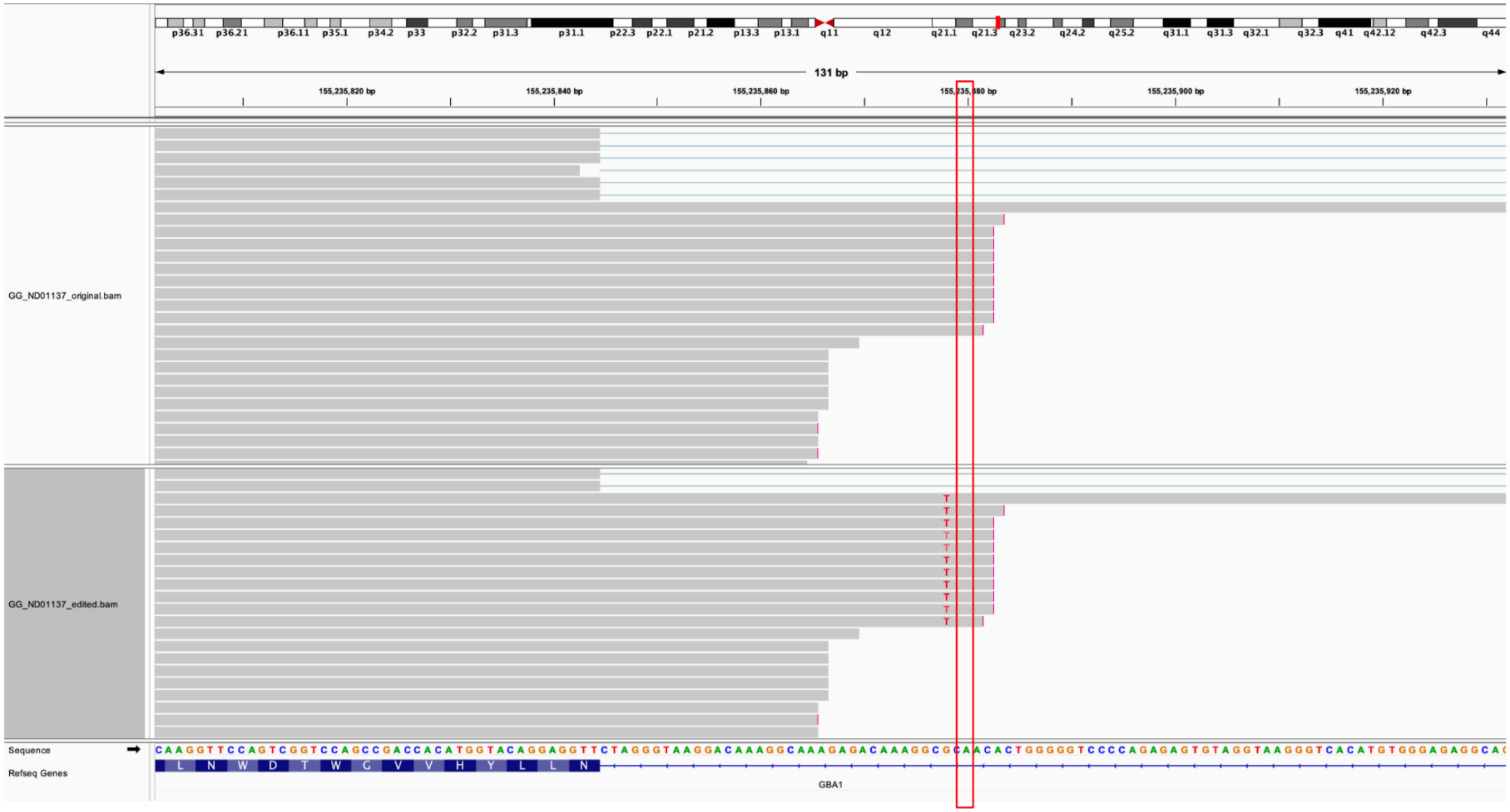

Supplementary Figure 4: Integrative Genome Browser inspection of Oxford Nanopore Technologies long-read RNA sequencing mapped reads in lymphoblastoid cell line ND01137 rs3115534-GG bam and *in silico* edited to ND01137 rs3115534-TT bam. a) Zoomed out IGV capture of *GBA1* reads in both files, black box denoting intron 8 area of interest. No obvious mapping differences are observed with the manually edited base. b) Identical to a) mapped reads zoomed in on rs3115534 to confirm successful manual *in silico* edit from GG to TT at this locus.

a)

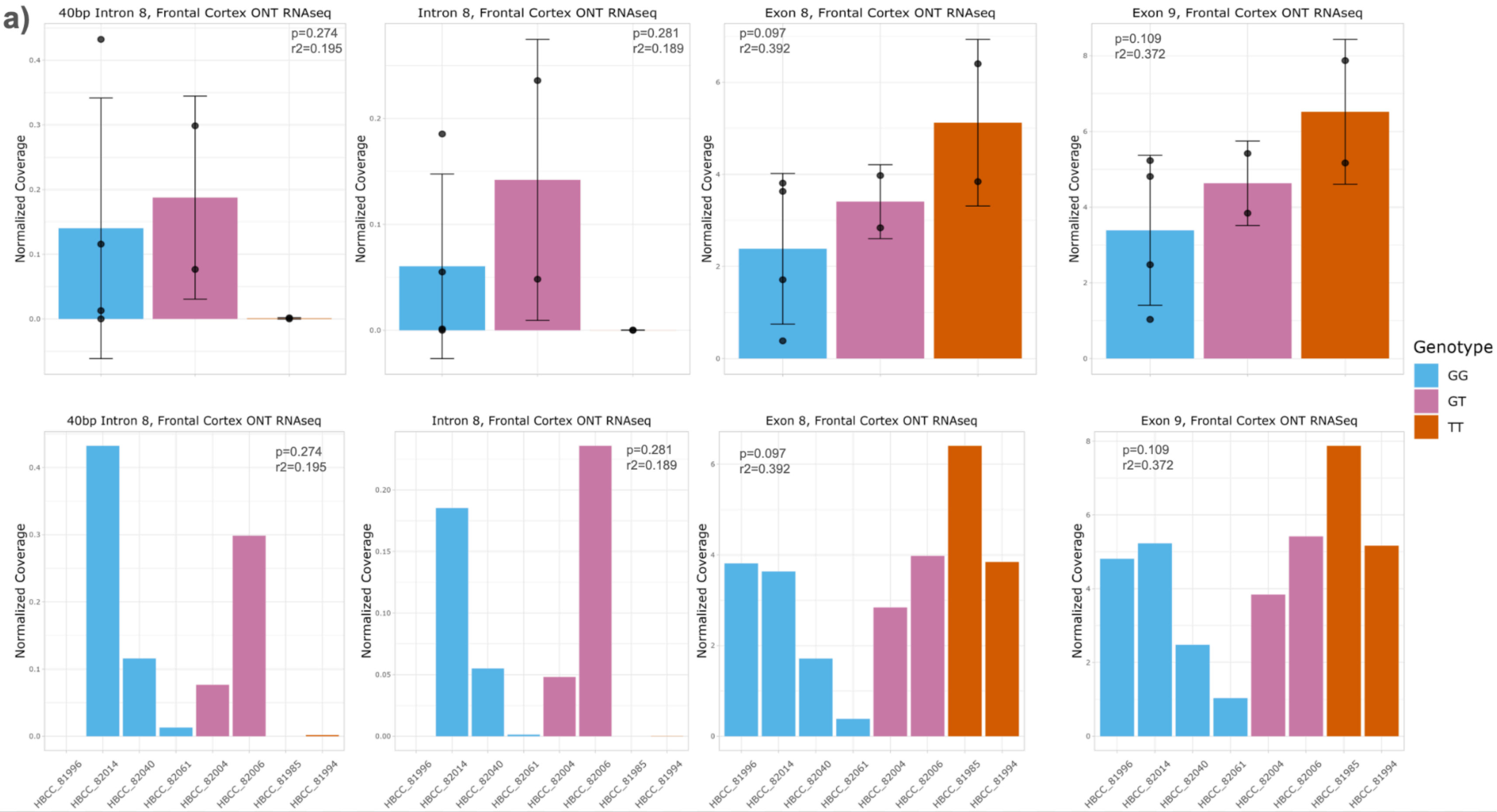

40bp Intron 8, Frontal Cortex ONT RNAseq
p=0.274
r2=0.195
Intron 8, Frontal Cortex ONT RNAseq
p=0.281
r2=0.189
Exon 8, Frontal Cortex ONT RNAseq
p=0.097
r2=0.392
Exon 9, Frontal Cortex ONT RNASeq
p=0.109
r2=0.372
Normalized Coverage
Genotype
GG
GT
TT
HBCC_81996
HBCC_82014
HBCC_82040
HBCC_82061
HBCC_82004
HBCC_82006
HBCC_81985
HBCC_81994

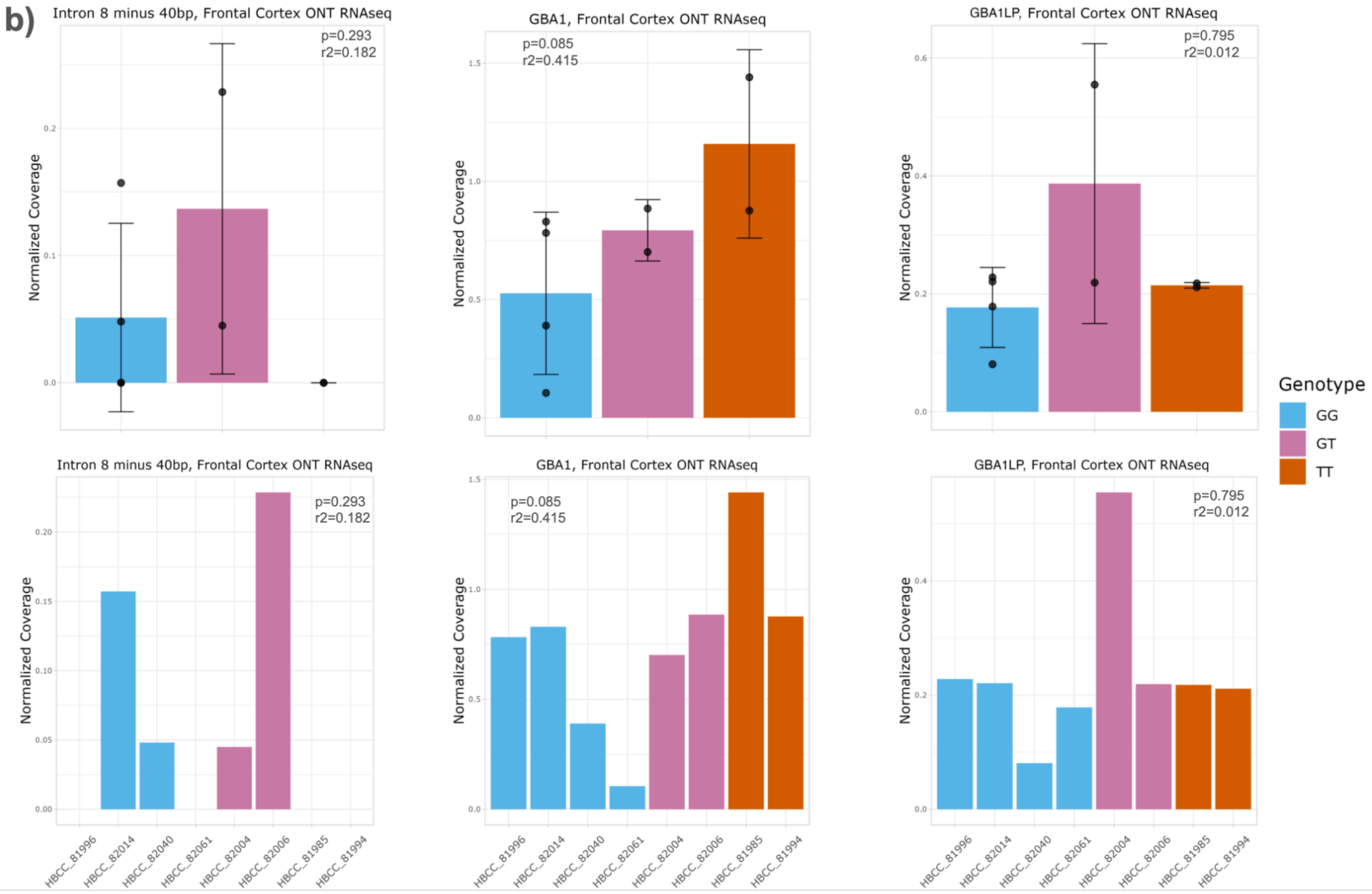
b)
Intron 8 minus 40bp, Frontal Cortex ONT RNAseq
p=0.293
r2=0.182
Normalized Coverage
GBA1, Frontal Cortex ONT RNAseq
p=0.085
r2=0.415
GBA1LP, Frontal Cortex ONT RNAseq
p=0.795
r2=0.012
Genotype
GG
GT
TT
HBCC_81996
HBCC_82014
HBCC_82040
HBCC_82061
HBCC_82004
HBCC_82006
HBCC_81985
HBCC_81994

Supplementary Figure 5: Regional coverage plots from human frontal cortex Oxford Nanopore Technologies long-read RNA sequencing showing enrichment of intron 8 expression in rs3115534-G carriers (n=8). a) Coverage plots of the highly expressed intron 8 40bp region, intron 8, exon 8, and exon 9. rs3115534-G carriers have a higher expression of intron 8 regions. Plots shown per sample and collapsed by genotype. b) Coverage plots of intron 8 minus 40bp region, *GBA1*, and *GBA1LP*. Plots shown per sample and collapsed by genotype. For all panels, a linear regression was run with GG + GT in one group versus TT. Error bars represent standard deviation for all panels with the center at the mean.

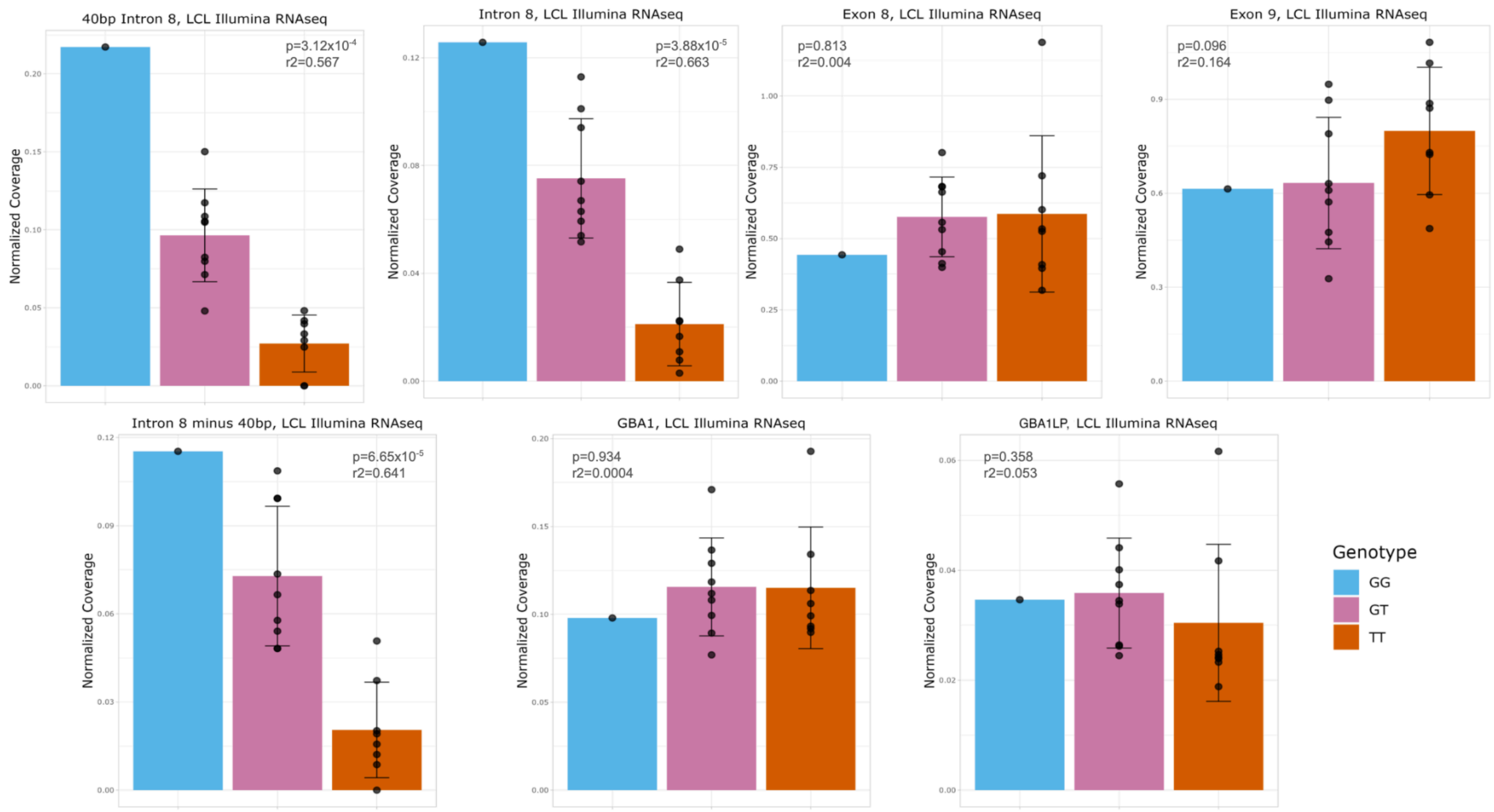

Supplementary Figure 6: Regional coverage plots from lymphoblastoid cell lines sequenced with Illumina RNA sequencing showing enrichment of intron 8 expression in rs3115534-G carriers (n=18). Coverage plots of regions of interest collapsed by genotype from lymphoblastoid cell lines similar as previous supplementary figures. Enrichment of intron 8 expression in G allele carriers still present with short-read sequencing. A linear regression was run with GG + GT in one group versus TT. Error bars represent standard deviation for all panels with the center at the mean.

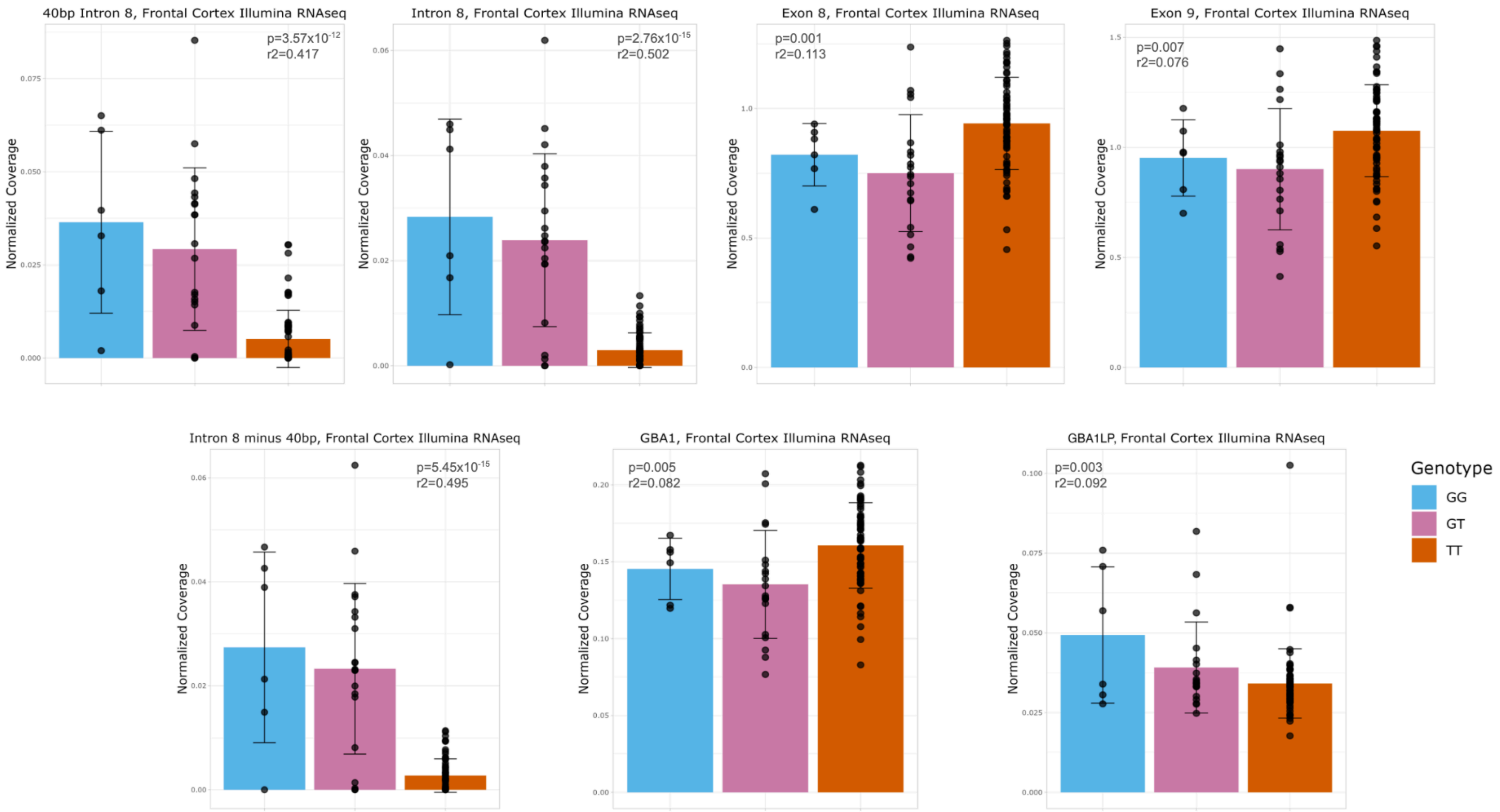

Supplementary Figure 7: Regional coverage plots from human frontal cortex Illumina short-read RNA sequencing showing enrichment of intron 8 expression in rs3115534-G carriers (n=92). Coverage plots of the highly expressed intron 8 40bp region, intron 8, exon 8, and exon 9, intron 8 minus 40bp region, *GBA1*, and *GBA1LP.* rs3115534-G carriers have a higher expression of intron 8 regions. Plots shown collapsed by genotype. A linear regression was run with GG/GT/TT in separate groups. Error bars represent standard deviation for all panels with the center at the mean.

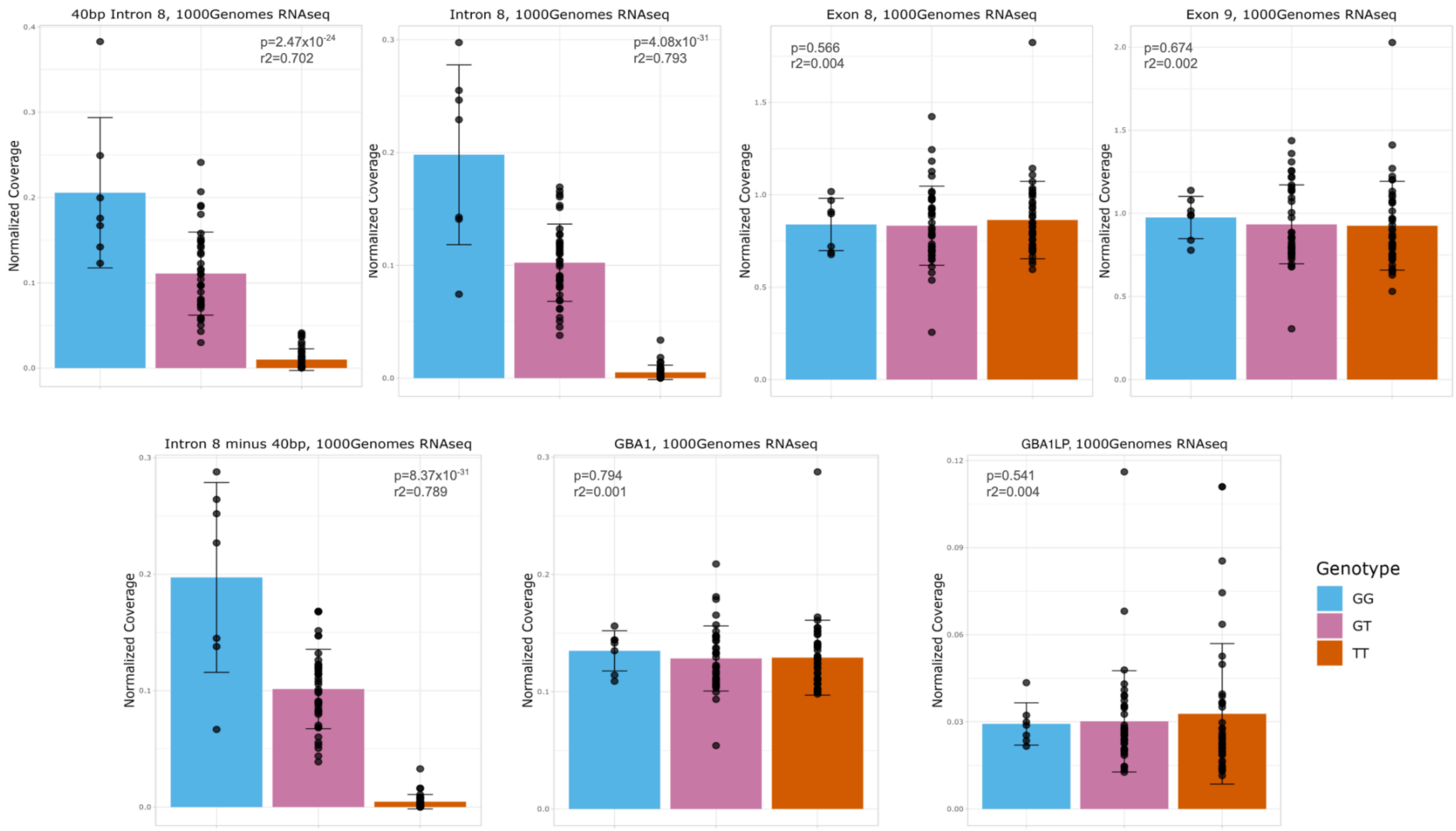

Supplementary Figure 8: Regional coverage plots from 1000 Genomes dataset showing enrichment of intron 8 expression in rs3115534-G carriers (n=88). Coverage plots of regions of interest collapsed by genotype similar as previous supplementary figures. Enrichment of intron 8 expression in rs3115534-G allele carriers still present with larger sample-sizes. A linear regression was run with GG/GT/TT in separate groups. Error bars represent standard deviation for all panels with the center at the mean.

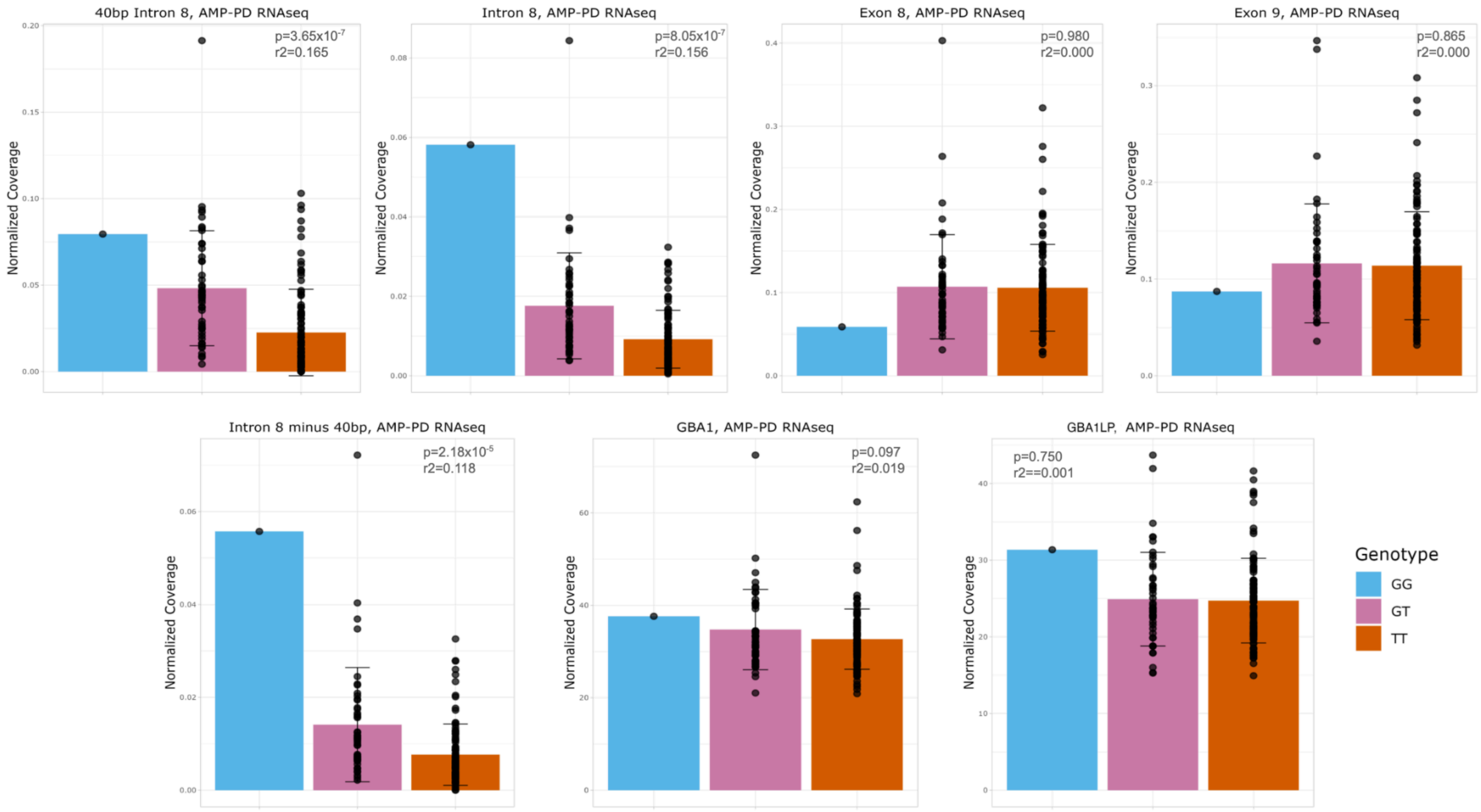

Supplementary Figure 9: Regional coverage plots from AMP-PD blood-based RNA sequencing showing enrichment of intron 8 expression in rs3115534-G carriers (n=146). Coverage plots of regions of interest collapsed by genotype similar as previous supplementary figures. Enrichment of intron 8 expression in rs3115534-G allele carriers still present in blood samples. A linear regression was run with GG + GT in one group versus TT. Error bars represent standard deviation for all panels with the center at the mean.

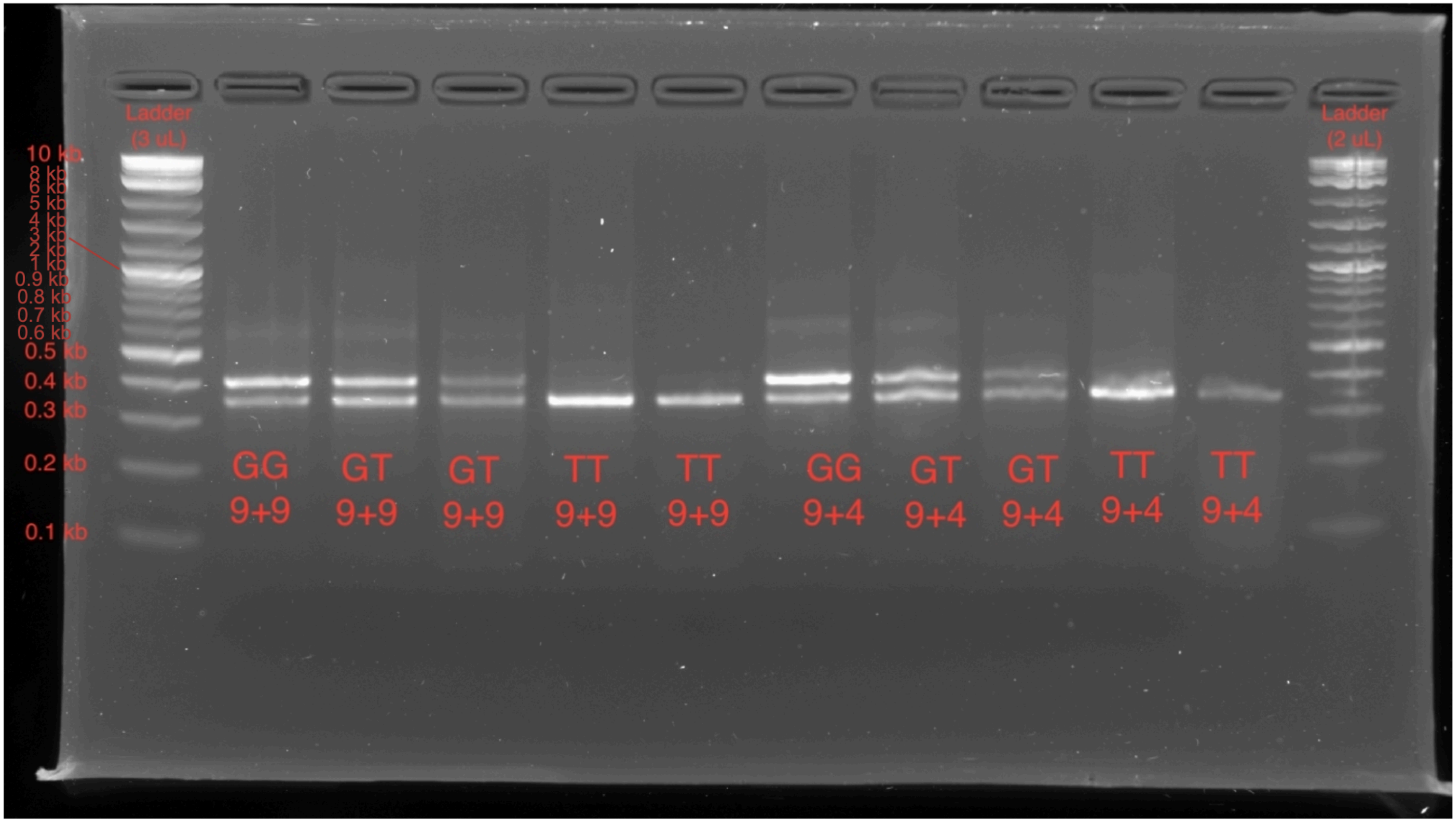

Supplementary Figure 10: Reverse transcription PCR of RNA from lymphoblastoid cell lines of various genotypes (GG, GT, or TT). RNA was isolated from LCLs of various genotypes (GG, GT, or TT) and converted to cDNA followed by PCR amplification with primer pairs 9F and 9R (9+9) as well as 9F and 4R (9+4) (**Supplementary Table 7**). All PCR products were run on 1% agarose gel twice.

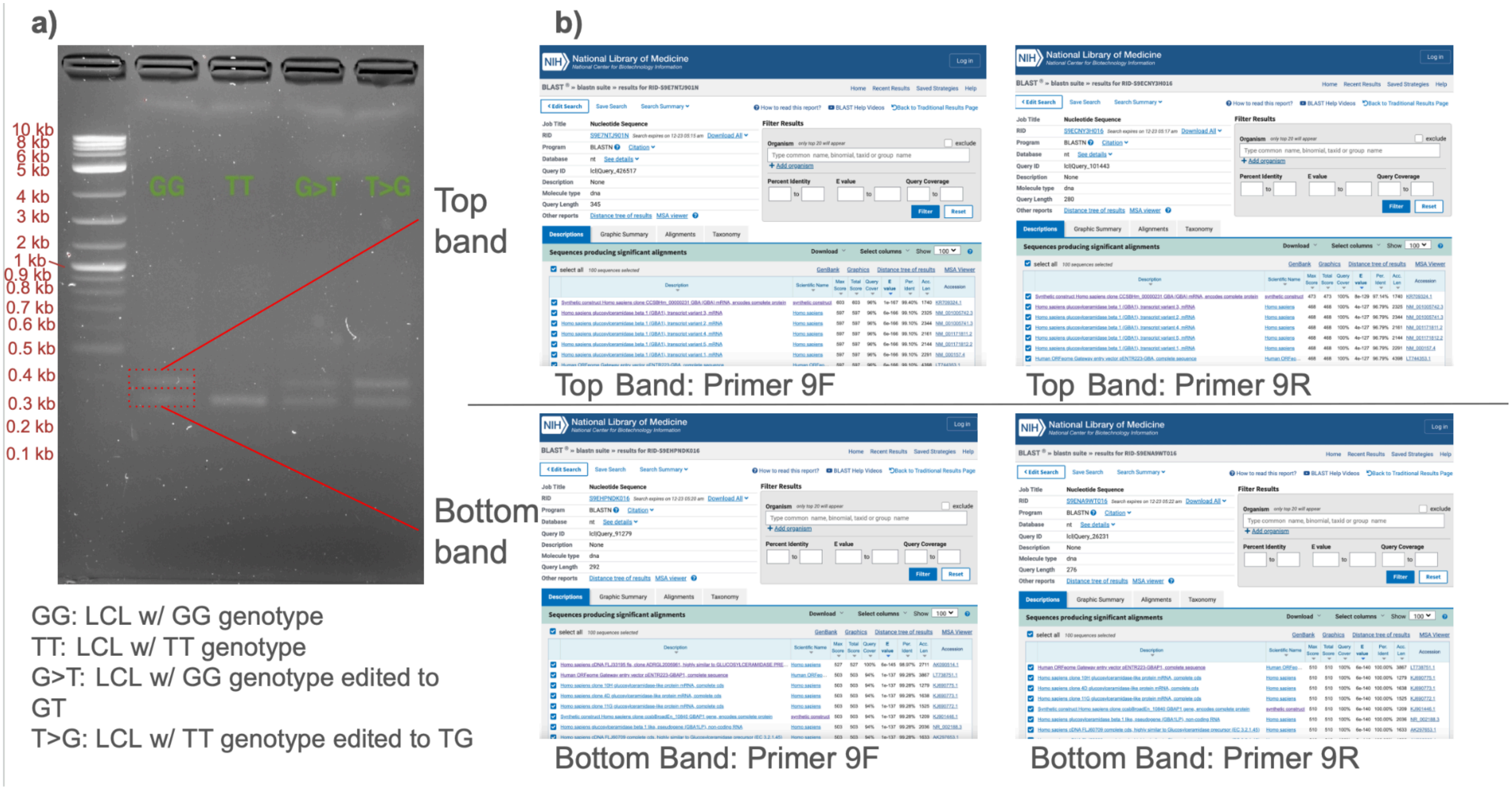

Supplementary Figure 11: Reverse transcription PCR products used for Sanger sequencing. a) 1% agarose gel containing PCR products that were excised and sent for Sanger sequencing. Sanger sequencing was performed once. b) BLAST results of sequences obtained from Sanger sequencing resulted in the top band to be *GBA1* transcript and the bottom band to be *GBA1LP* showing the 55bp deletion difference between *GBA1* and *GBA1LP* (See full BLAST results in **Supplementary Table 12**).

a)

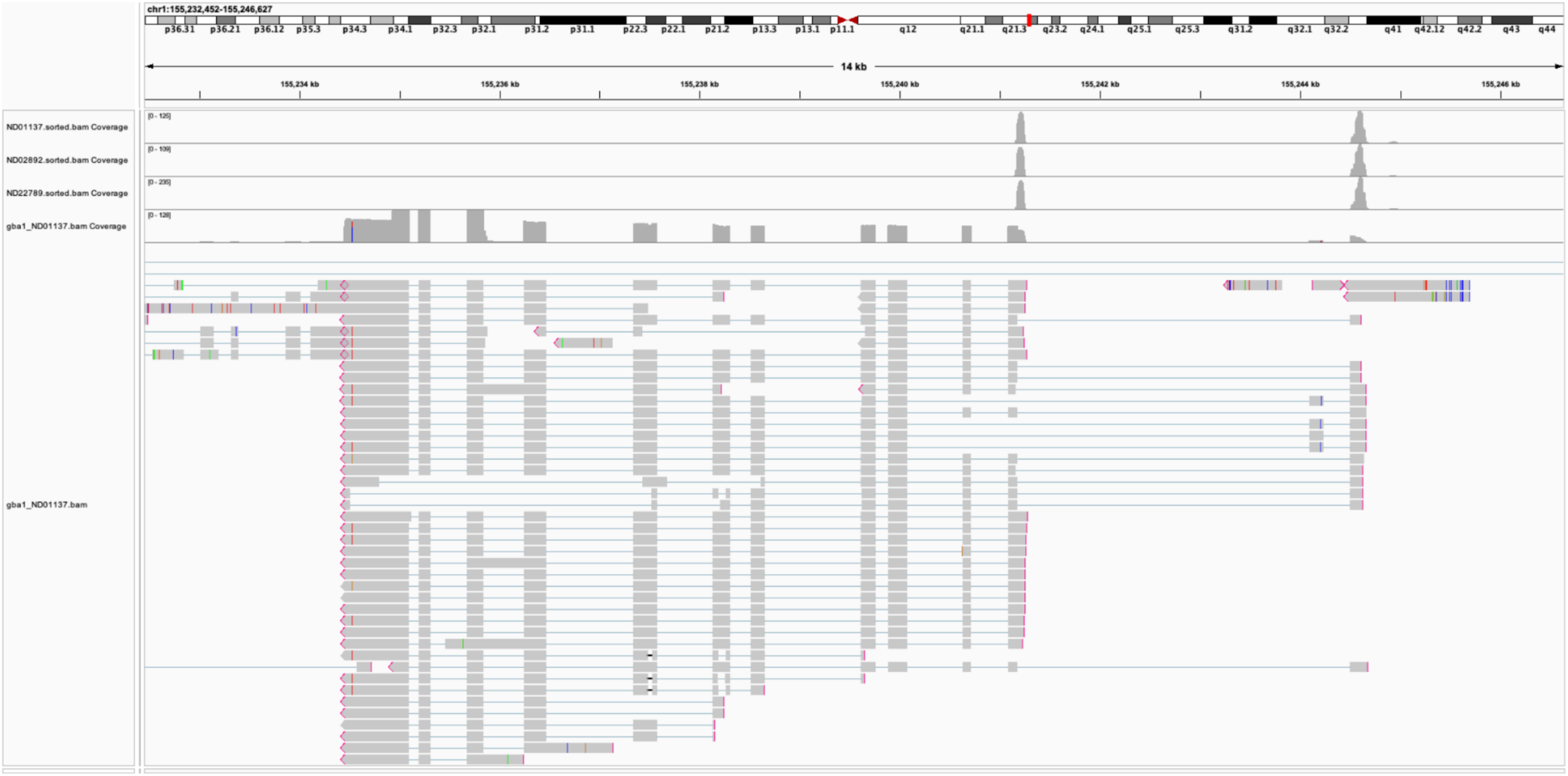
chr1:155,232,452-155,246,627
14 kb
155,234 kb
155,236 kb
155,238 kb
155,240 kb
155,242 kb
155,244 kb
155,246 kb
ND01137.sorted.bam Coverage
ND02892.sorted.bam Coverage
ND22789.sorted.bam Coverage
gba1_ND01137.bam Coverage
gba1_ND01137.bam

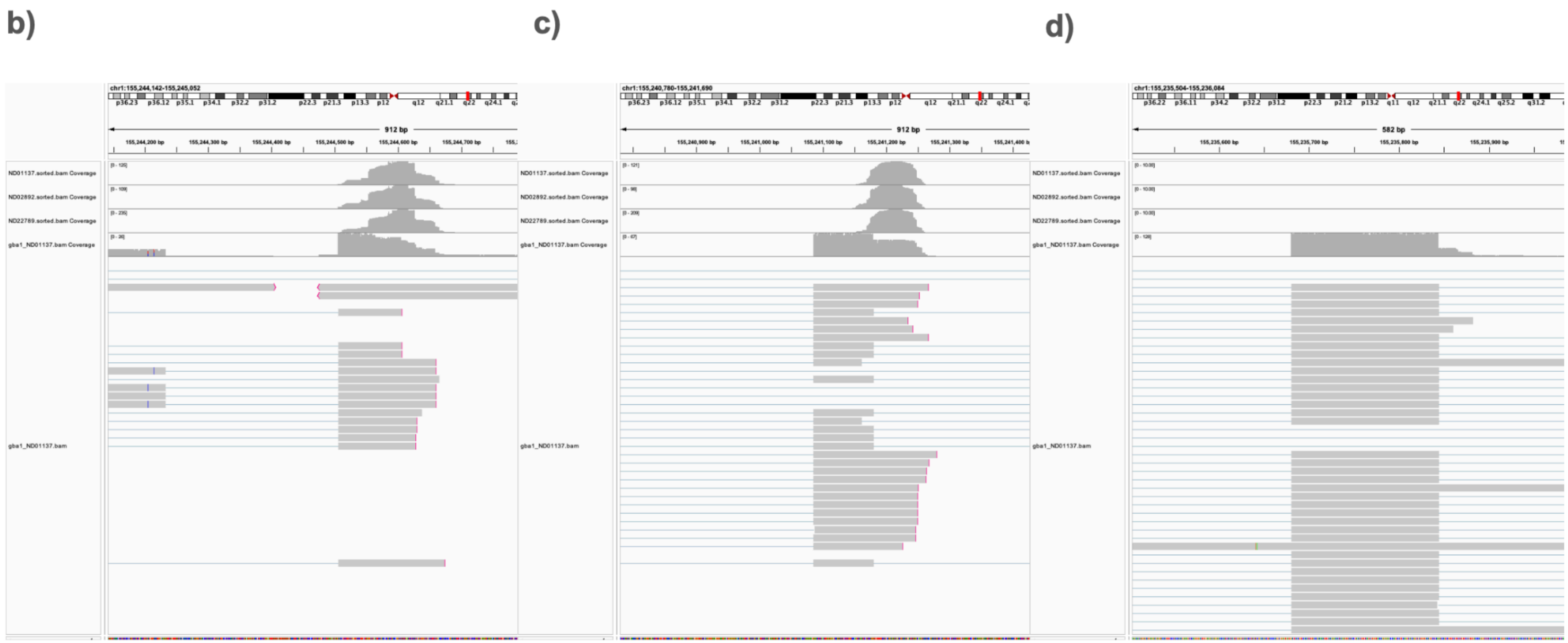

Supplementary Figure 12: CAGEseq sequencing rs3115534-GG, GT, TT to assess transcription start sites across genotypes. a) Full view of the *GBA1* gene resulted in the identification of two transcription start sites at exons 1 and 2. No sequence reads were detected at the exon 9 region. b) Zoomed in IGV screenshot of main transcription start site. c) Zoomed in IGV screenshot of secondary transcription start site. d) Zoomed in IGV screenshot of intron 8 region.

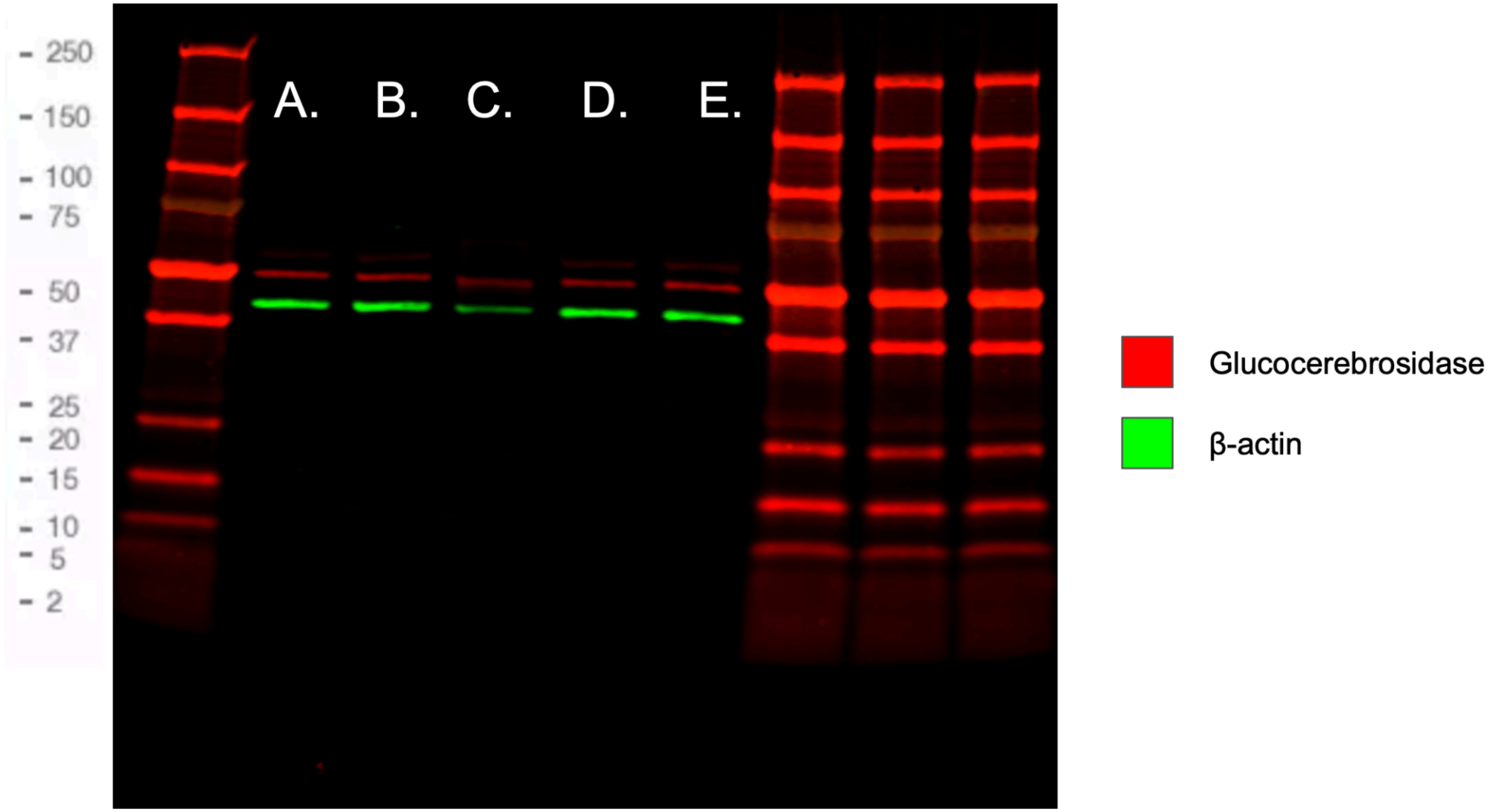

Supplementary Figure 13: Western blot of various lymphoblastoid cell lines rs3115534 genotypes to assess protein coding ability. 30 ug of LCL protein lysate was loaded into each well. **A.** *GBA1* p.N409S Gaucher Disease Type 1 **B.** rs3115534-GG (replicate 1) **C.** rs3115534-GG (replicate 2) **D.** rs3115534-GT **E.** rs3115534-TT. Glucocerebrosidase (57.9 kD) is represented in red while β-actin (42 kD) is in green. Western blot was run three times with both ECL and fluorescent multiplex methods.

a)

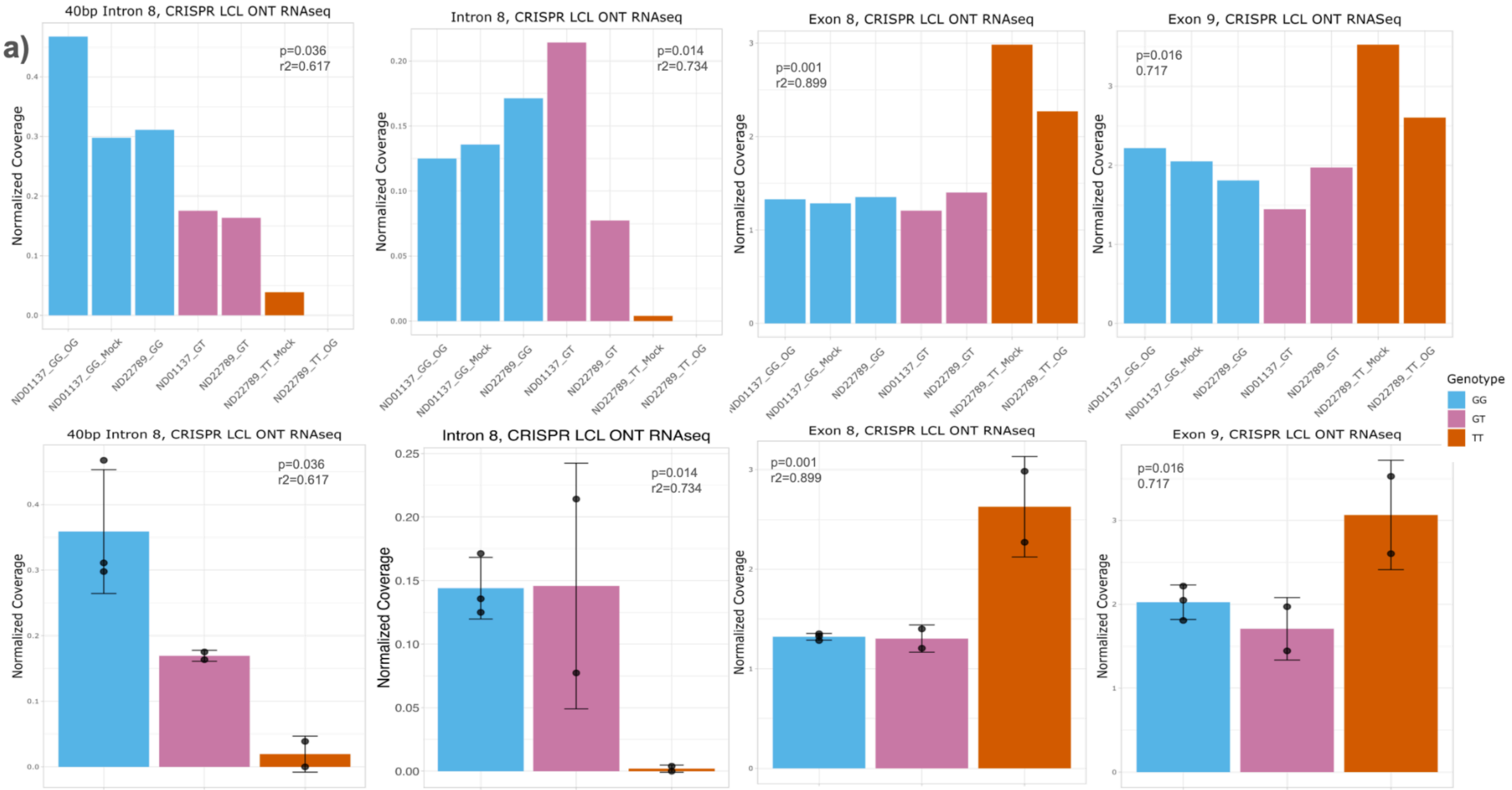
40bp Intron 8, CRISPR LCL ONT RNAseq
p=0.036
r2=0.617
Intron 8, CRISPR LCL ONT RNAseq
p=0.014
r2=0.734
Exon 8, CRISPR LCL ONT RNAseq
p=0.001
r2=0.899
Exon 9, CRISPR LCL ONT RNASeq
p=0.016
0.717
Normalized Coverage
ND01137_GG_OG
ND01137_GG_Mock
ND22789_GG
ND01137_GT
ND22789_GT
ND22789_TT_Mock
ND22789_TT_OG
Genotype
GG
GT
TT

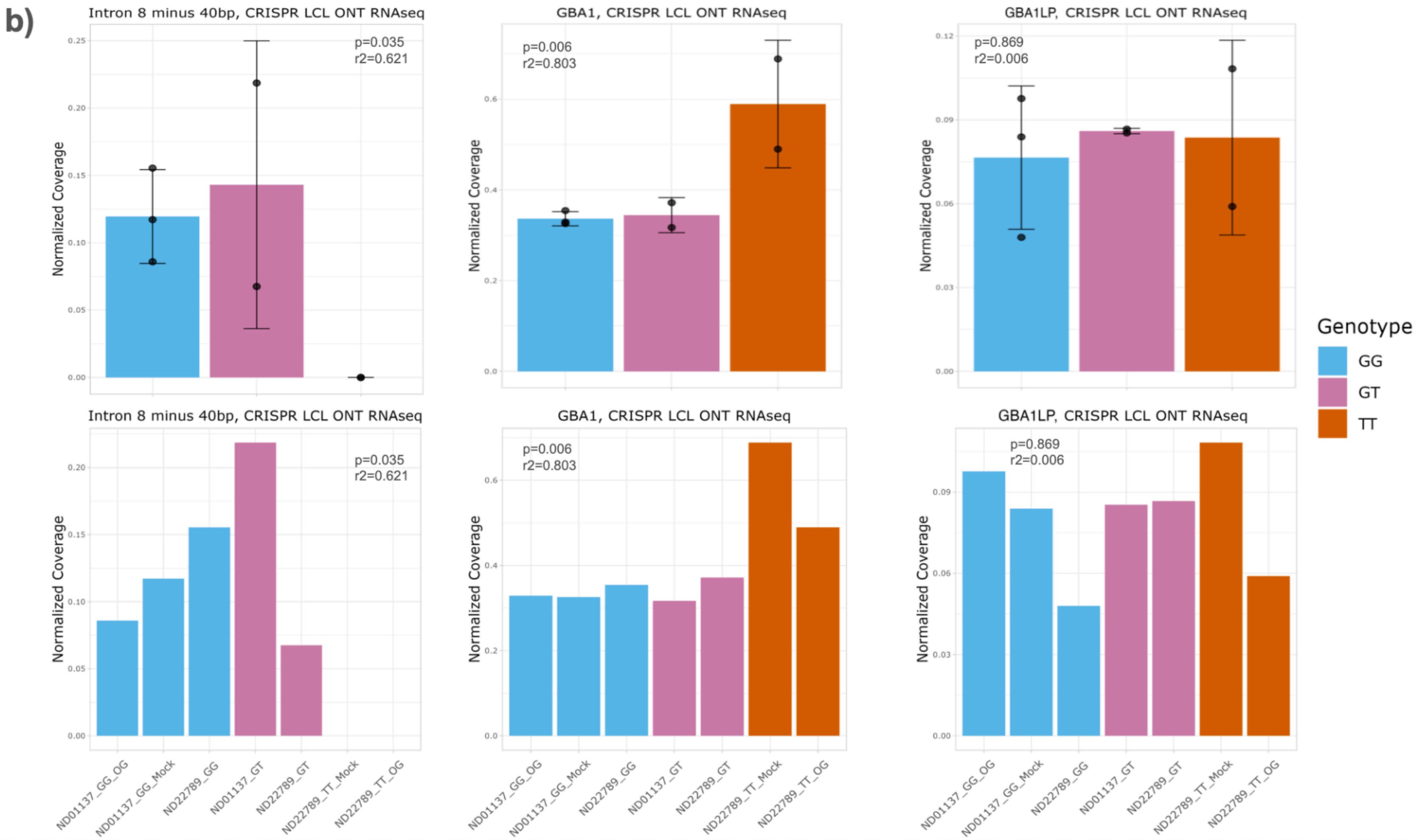

Supplementary Figure 14: Additional coverage plots for CRISPR-edited lymphoblastoid cell lines sequenced with Oxford Nanopore Technologies long read RNA sequencing (n=7). a) Quantification of intron 8, exon 8, and exon 9 expression CRISPR-edited lines. **b)** Additional coverage plots from CRISPR-edited LCLs for intron 8 minus 40bp transcript region, *GBA1*, and *GBA1LP*. For all regions, plots are shown both per sample and collapsed by genotype. Coverage for all panels normalized by dividing mean depth by total number of mapped reads per million as detailed in methods. For all panels, a linear regression was run with GG + GT in one group versus TT. Error bars represent standard deviation for all panels with the center at the mean

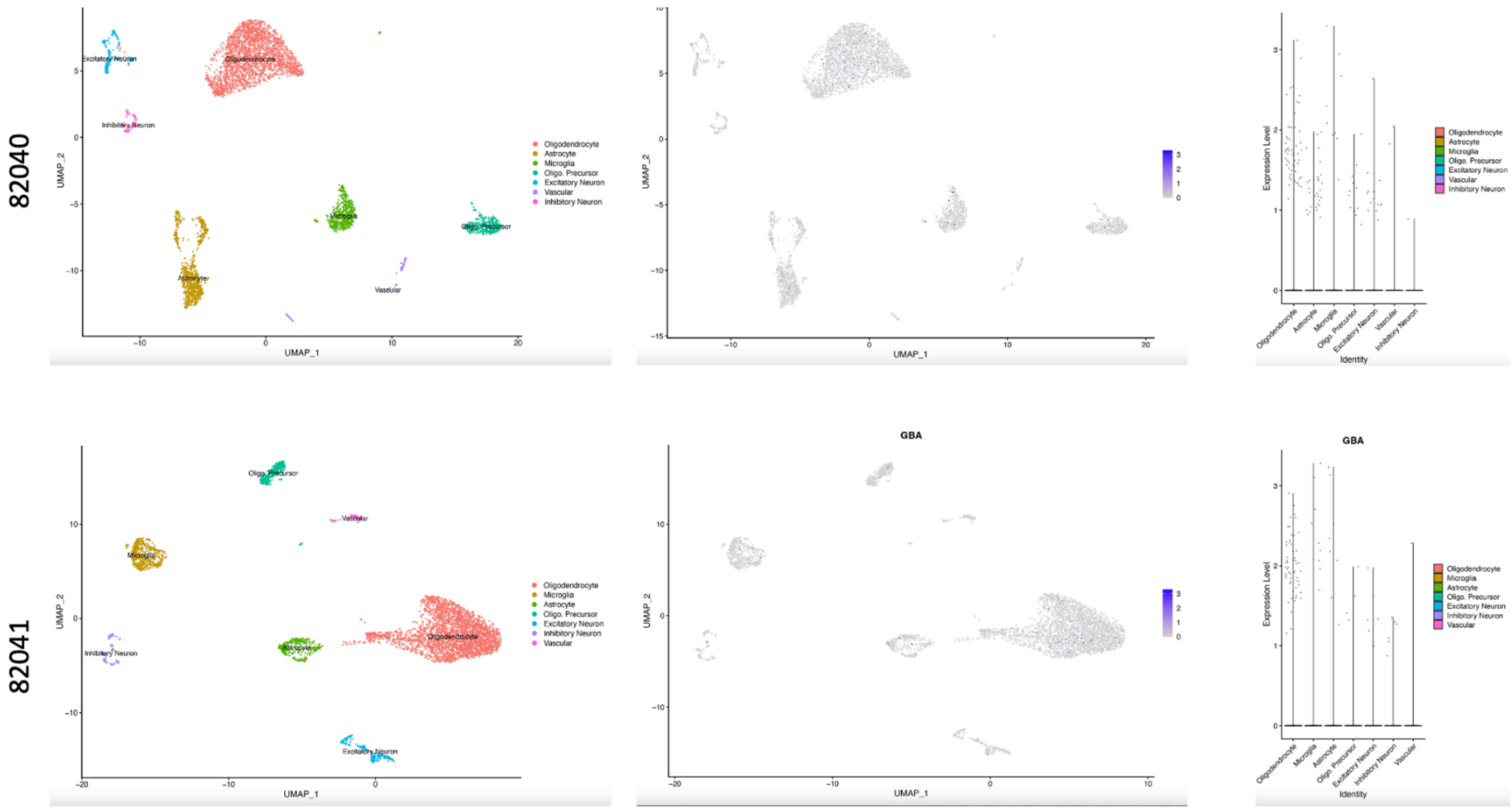

Supplementary Figure 15: Low *GBA1* gene expression across major brain cell types using single nuclei RNA sequencing. UMAP of single nuclei RNA sequencing of two frontal cortex brain tissue samples (1x rs3115534-GG and 1x rs3115534-TT) from 10X Illumina data shows clear clustering of 7 major brain cell types. *GBA1* expression differences across cell types can also be picked up, however *GBA1* expression is generally low.

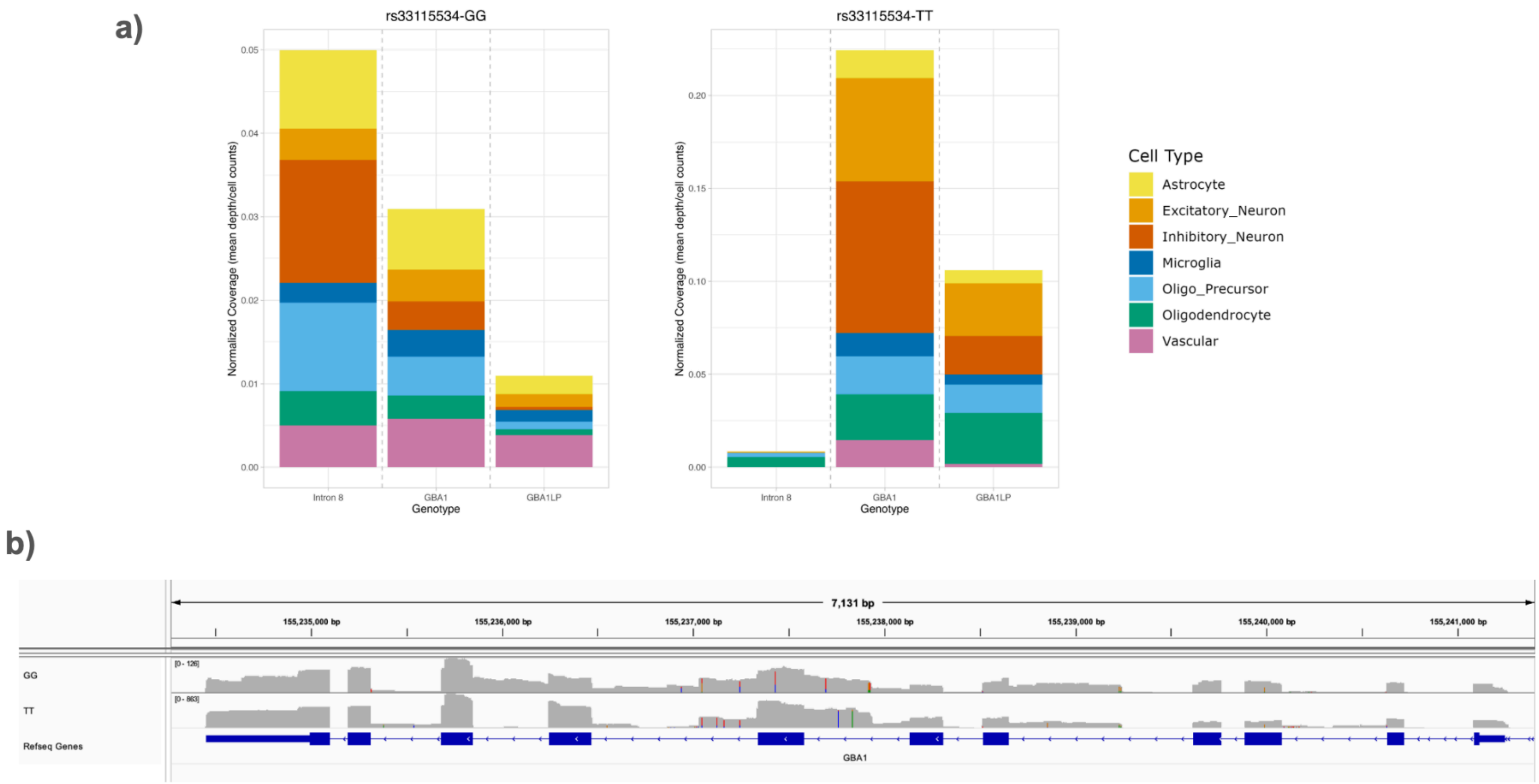

Supplementary Figure 16: Enriching of *GBA1* in single nuclei RNA sequencing shows *GBA1* expression across major brain cell types. a) Enrichments of *GBA1* transcripts of two frontal cortex brain tissue samples (1x rs3115534-GG and 1x rs3115534-TT) using probes resulted in a clear increase of transcripts to be identified. Differences were observed between GG and rs3115534-GG and rs3115534-TT in reads covering intron 8 but no clear cell-type enrichment was identified for intron 8 expression. **b)** IGV screenshot comparing the coverage track over *GBA1* for rs3115534-GG and rs3115534-TT. A difference in intron 8 expression is observed.

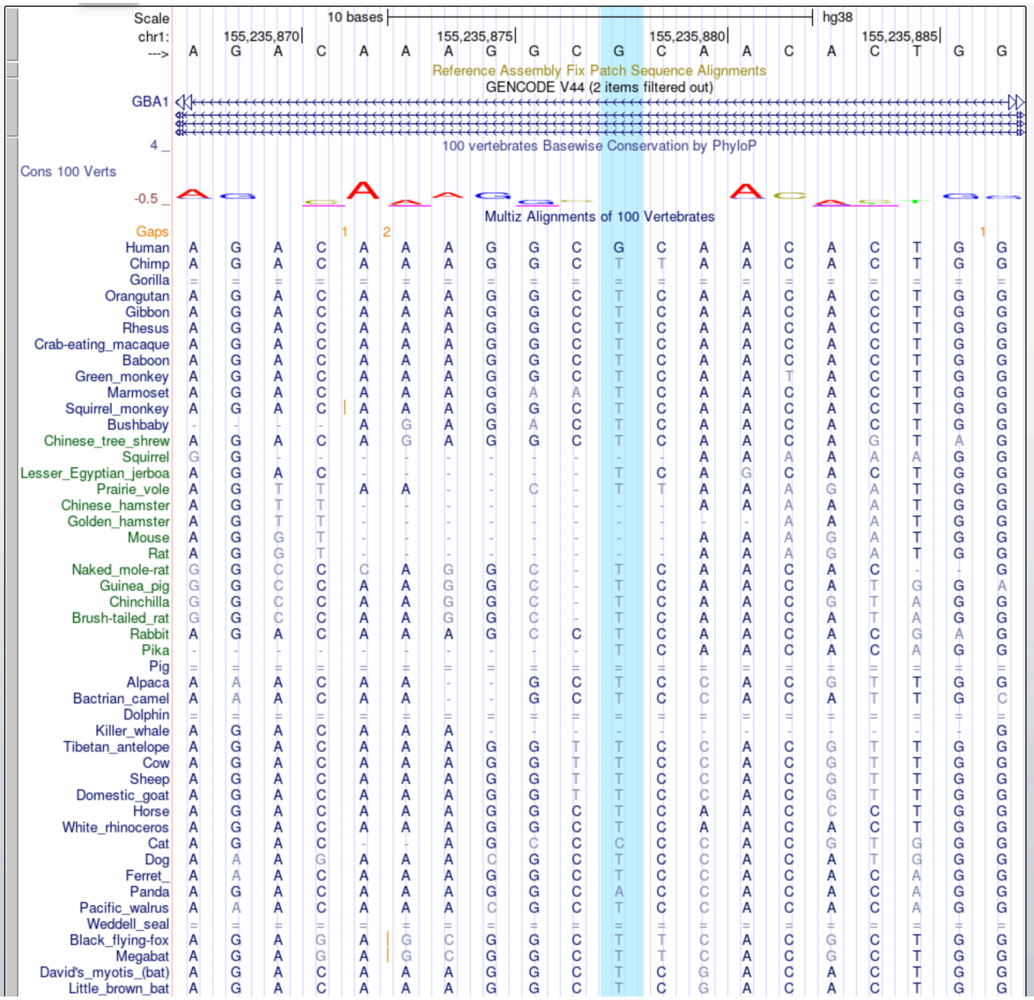

Supplementary Figure 17: Assessment of conservation across species for rs3115534. The UCSC Genome Browser was accessed to investigate the conservation of this allele across vertebrates. Very high conservation was observed for the T allele in this part of the genome and humans are the only ones reported to have a G allele here as reference allele.

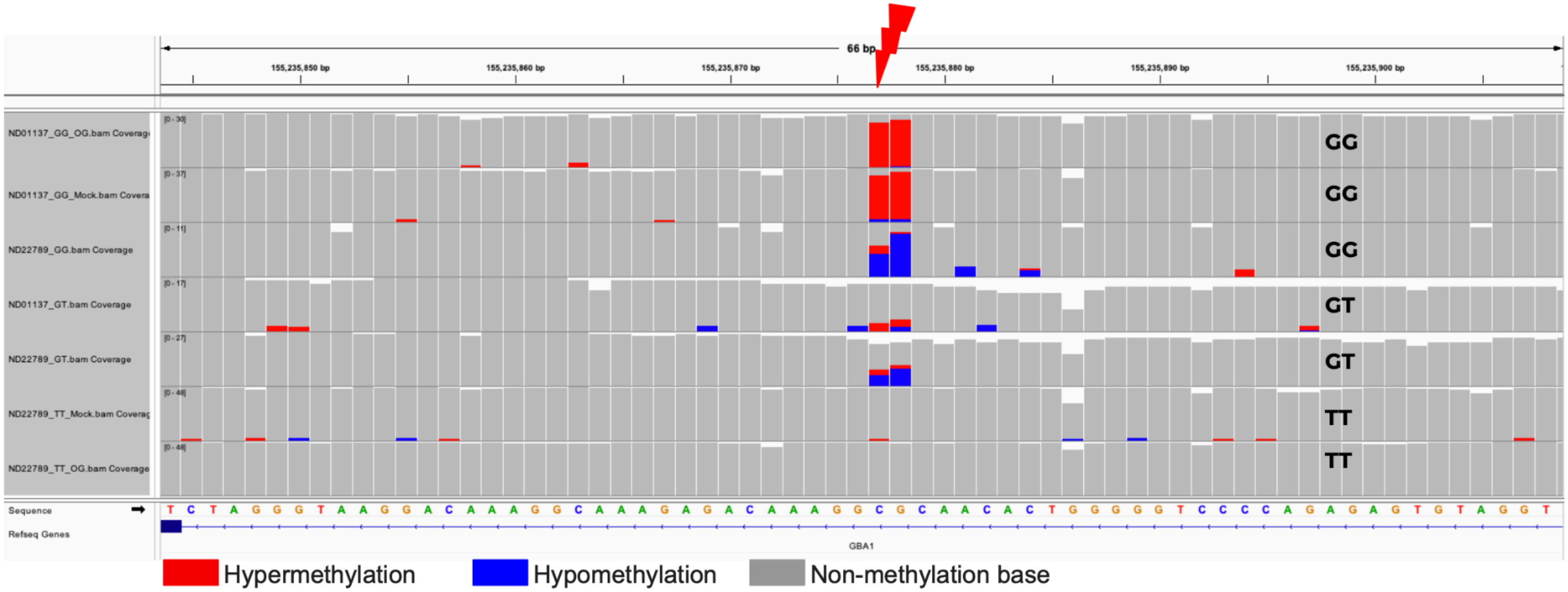

Supplementary Figure 18: Methylation differences in rs3115534 across CRISPR-edited lymphoblastoid cell lines. Methylation information obtained from Oxford Nanopore Technologies DNA sequencing. When the rs3115534-G risk allele is present, the locus turns into a methylation site. When rs3115534-T non-risk allele is present, the methylation site is lost.

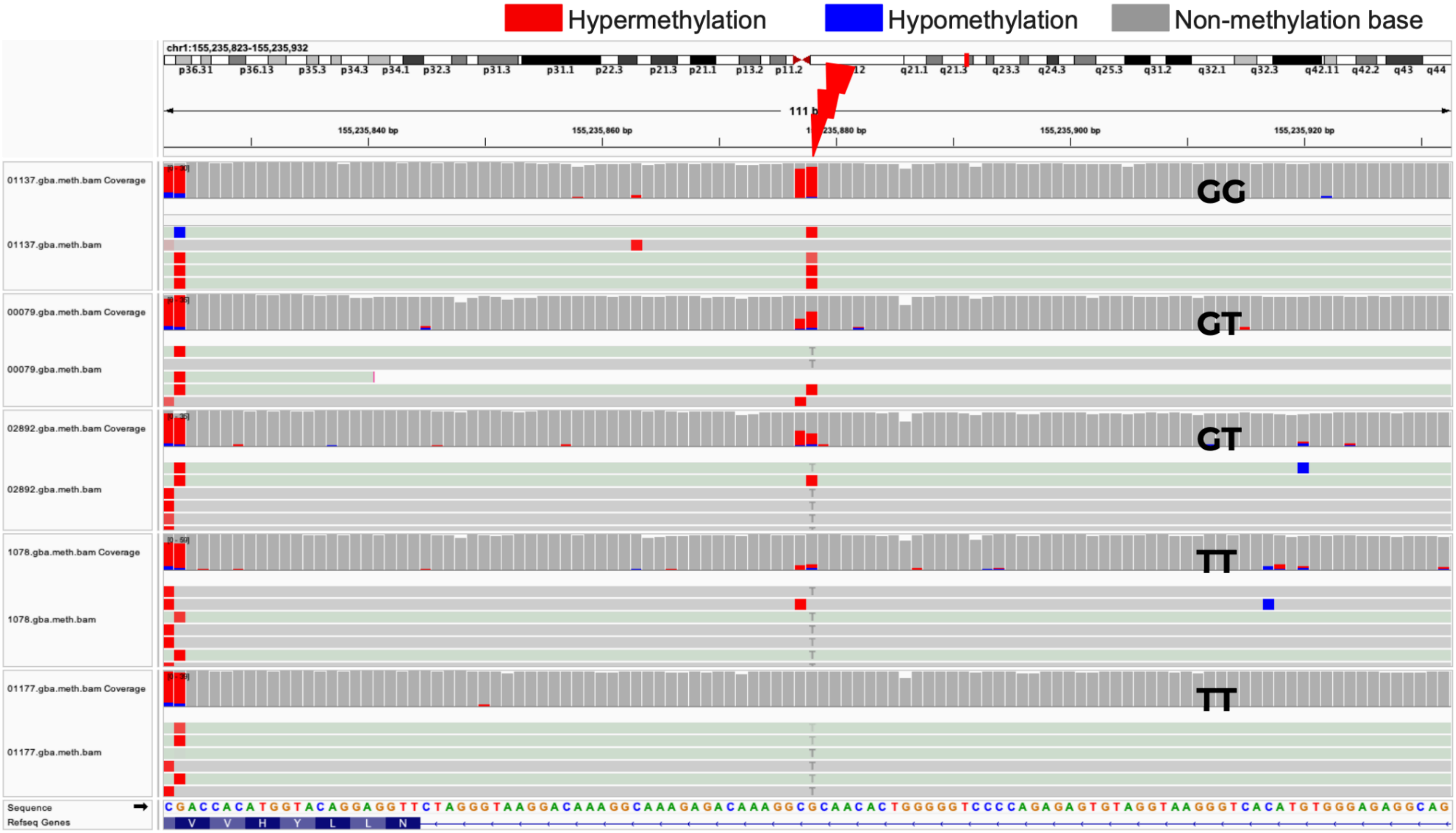

Supplementary Figure 19: Methylation differences in rs3115534 across lymphoblastoid cell lines. Methylation information obtained from Oxford Nanopore Technologies DNA sequencing. When the rs3115534-G risk allele is present, the locus turns into a methylation site. When T-non-risk allele is present, the methylation site is lost.

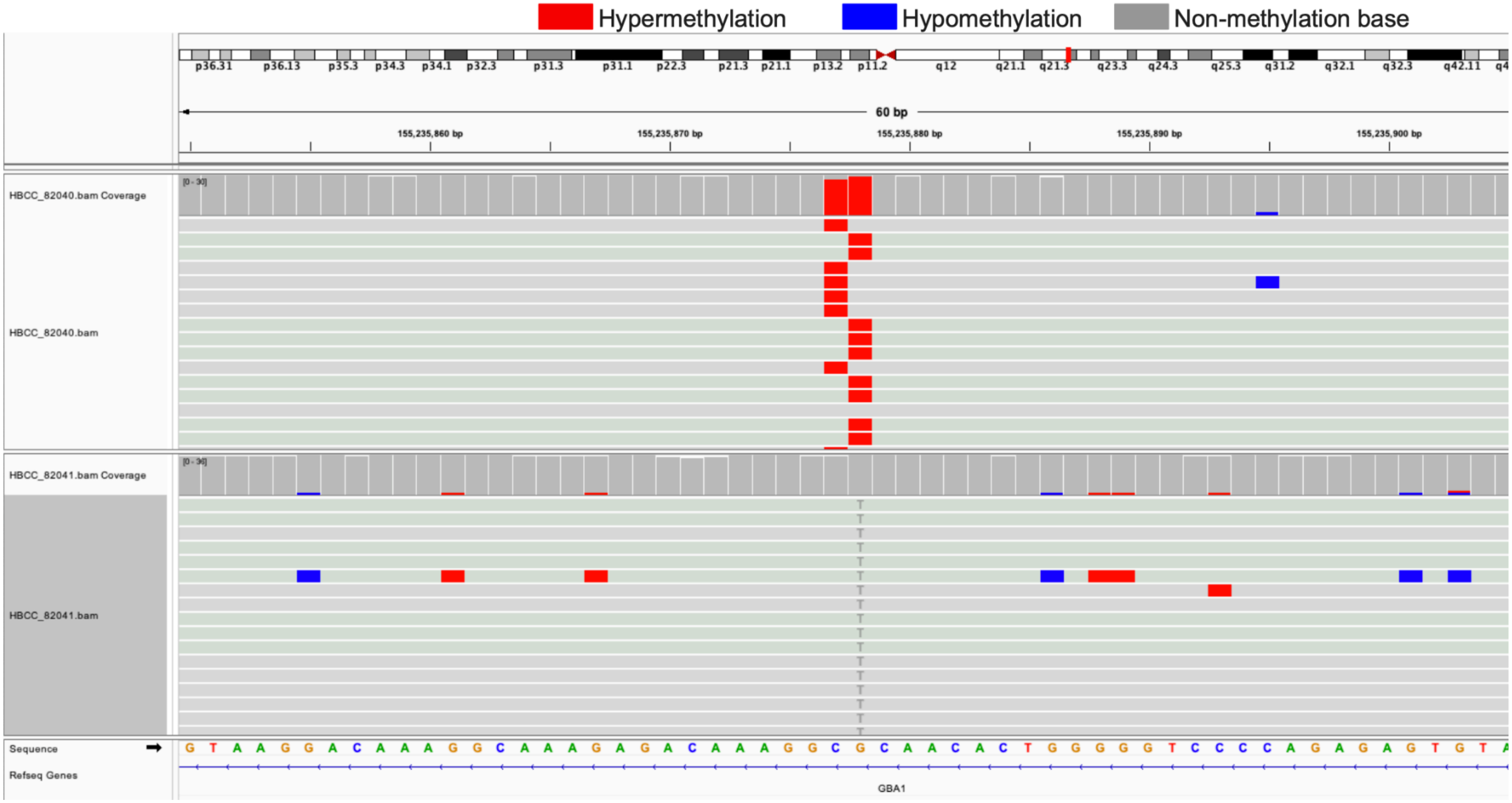

Supplementary Figure 20: Methylation differences in rs3115534 across frontal cortex samples. Methylation information obtained from Oxford Nanopore Technologies DNA sequencing. HBCC_82040 is rs3115534-GG (top track), while HBCC_82041 (bottom track) is rs3115534-TT. When the rs3115534-G risk allele is present, the locus turns into a methylation site. When T-non-risk allele is present, the methylation site is lost.

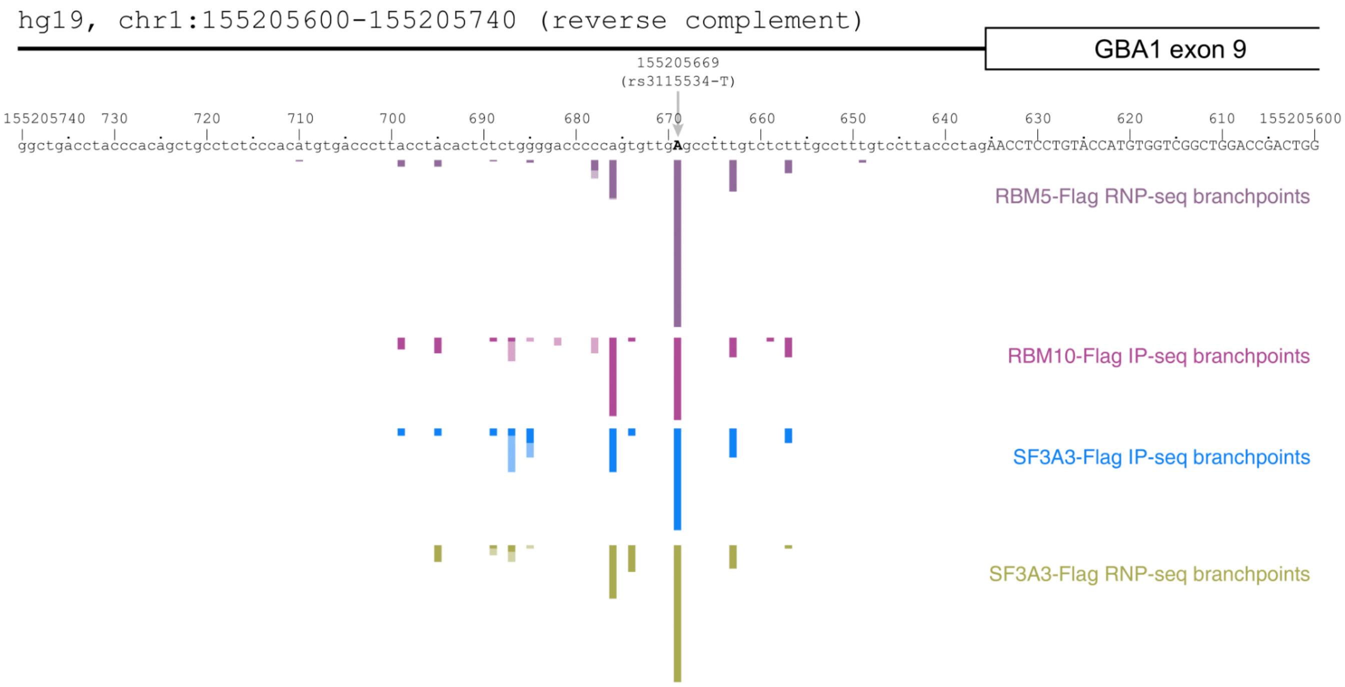

Supplementary Figure 21: rs3115534-T in human 293Flp-in cells is engaged as a branchpoint of *GBA1* intron 8. Branchpoint nucleotides were predicted from branch site reads unambiguously aligning with *GBA1*. These are represented by vertical bars with heights proportional to frequency of detection. Branchpoints from reads that may have originated from either *GBA1* or *GBA1LP* are shown in lighter color. Prediction was performed separately for each available branch site dataset (SF3A3, RBM5, RBM10) as indicated.

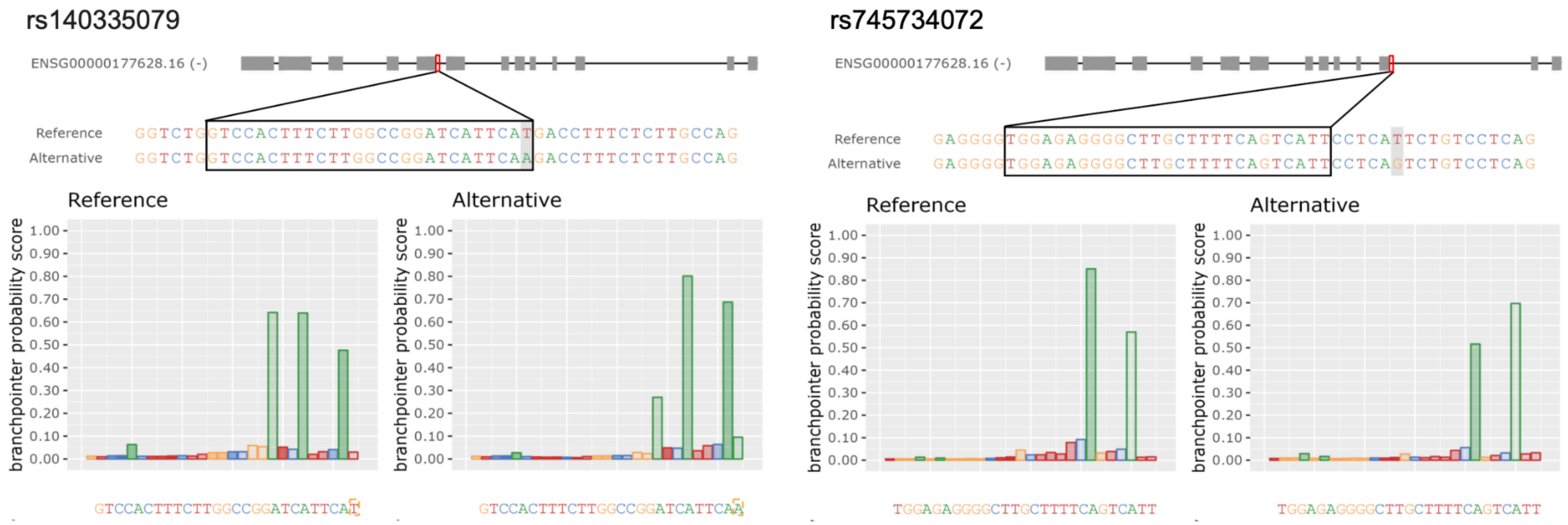

Supplementary Figure 22: Branchpoint investigation of rs140335079 and rs745734072. rs140335079 is reported by AGAIN to be in a branchpoint sequence. The A is the reference allele and T is the alternative allele. No drastic differences were observed in the Branchpointer probability scores. rs745734072 is reported by AGAIN to be in a branchpoint sequence. The A is the reference allele and C is the alternative allele. No drastic differences were observed in the Branchpointer probability scores.

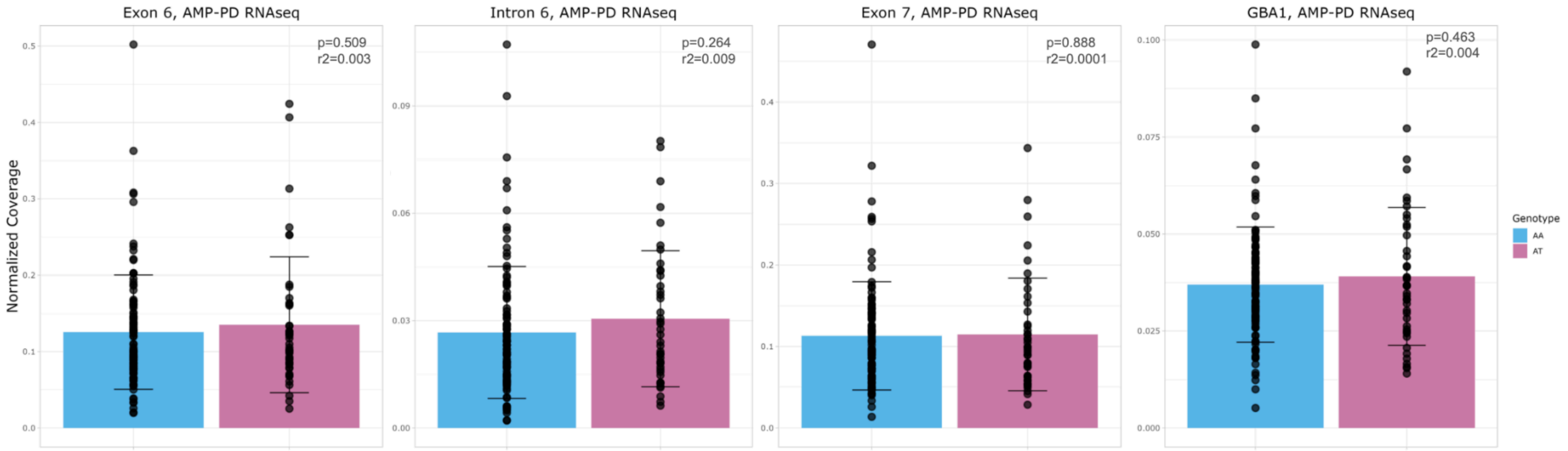

Supplementary Figure 23: No expression differences identified due to rs140335079. rs140335079 is reported by AGAIN to affect a branchpoint sequence, however no drastic differences were observed in the Branchpointer probability scores. To confirm that the was no similar branchpoint disruption mechanism like in rs3115534, expression around rs140335079 was plotted using AMP-PD blood-based RNAseq data (n=146) and no important intron retention events were observed based on genotype. A linear regression was run with AA versus TT. Error bars represent standard deviation for all panels.

Supplementary Tables

SampleID	Sample Type	Sample Origin	Neurologic Diagnosis	rs3115534	RIN	Assay Performed	Sex	Age at Sampling
ND01137	LCL	Coriell	Parkinson's Disease	GG	9.5	Illumina-RNA, ONT-DNA, ONT-RNA, CRISPR	Female	64
ND00079	LCL	Coriell	Parkinson's Disease	GT	9.3	Illumina-RNA, ONT-DNA, ONT-RNA	Male	60
ND02892	LCL	Coriell	Parkinson's Disease	GT	9.5	Illumina-RNA, ONT-DNA, ONT-RNA	Female	58
ND02895	LCL	Coriell	Control	GT	9.6	Illumina-RNA	Male	64
ND05171	LCL	Coriell	Parkinson's Disease	GT	9.4	Illumina-RNA, ONT-DNA, ONT-RNA	Female	61
ND12179	LCL	Coriell	Control	GT	9.5	Illumina-RNA	Female	62
ND15296	LCL	Coriell	Control	GT	9.2	Illumina-RNA	Male	59
ND24020	LCL	Coriell	Control	GT	9.8	Illumina-RNA	Female	58
ND02896	LCL	Coriell	Control	GT	9.3	Illumina-RNA	Male	56
ND04724	LCL	Coriell	Parkinson's Disease	GT	9.5	Illumina-RNA, ONT-DNA, ONT-RNA	Male	56
ND01177	LCL	Coriell	Parkinson's Disease	TT	9.4	Illumina-RNA, ONT-DNA, ONT-RNA	Female	63
ND06940	LCL	Coriell	Parkinson's Disease	TT	9.2	Illumina-RNA	Male	62
ND11329	LCL	Coriell	Control	TT	9.5	Illumina-RNA	Female	58
ND13434	LCL	Coriell	Control	TT	9.6	Illumina-RNA	Female	58
ND22789	LCL	Coriell	Control	TT	9.4	Illumina-RNA, ONT-DNA, ONT-RNA, CRISPR	Male	61
ND24062	LCL	Coriell	Control	TT	9.3	Illumina-RNA	Male	62

ND25221	LCL	Coriell	Control	TT	9.2	Illumina-RNA, ONT-DNA, ONT-RNA	Male	57
ND01078	LCL	Coriell	Parkinson's Disease	TT	9.2	Illumina-RNA, ONT-DNA, ONT-RNA	Female	58
HBCC_82014	Brain frontal cortex	HBCC brain bank	Control	GG	6.9	ONT-DNA, ONT-RNA	Male	18.1
HBCC_81996	Brain frontal cortex	HBCC brain bank	Control	GG	5.1	ONT-DNA, ONT-RNA	Female	66.1
HBCC_82040	Brain frontal cortex	HBCC brain bank	Control	GG	6.3	ONT-DNA, ONT-RNA, ONT-10X	Female	30
HBCC_82061	Brain frontal cortex	HBCC brain bank	Control	GG	5.7	ONT-DNA, ONT-RNA	Male	57.1
HBCC_82004	Brain frontal cortex	HBCC brain bank	Control	GT	6.5	ONT-DNA, ONT-RNA	Female	45.4
HBCC_82006	Brain frontal cortex	HBCC brain bank	Control	GT	7.5	ONT-DNA, ONT-RNA	Male	27.3
HBCC_81985	Brain frontal cortex	HBCC brain bank	Control	TT	8.6	ONT-DNA, ONT-RNA	Male	42
HBCC_81994	Brain frontal cortex	HBCC brain bank	Control	TT	7.7	ONT-DNA, ONT-RNA	Male	38.8
HBCC_82041	Brain frontal cortex	HBCC brain bank	Control	TT	NA*	ONT-10X	Male	20.2

Supplementary Table 1: Biosamples used for assessment of effects of *GBA1* rs3115534. Lymphoblastoid cell lines (LCLs) from Coriell Institute for Medical Research and frontal cortex tissue samples from the Human Brain Collection Core of GG- GT- and TT-rs3115534 genotypes were used. Sample type (LCLs or front cortex), origin, genotype, RNA Integrity numbers (RIN), and downstream analyses are provided for each sample.

chr1	start (hg19)	end (hg19)	width	strand	SequenceName	Group.Name	Sequence	Request.ID	GC.Percent	Exonic_Intronic
chr1	155208169	155208289	121	-	747793_36360005_GBA(2629).3_5	GBA(2629)	TCAGGCCCTGAGGGCCCTGATGTCTGGGGGTTGAGAAACTGTAGGGTAGGTCTGCTTGTACAGACATTTTGTCCCCTGCTGTTTTGTCCTGGGGGTGGGAGGGTGGAGGCTAATGGCTGA	747793	56.67	Intronic
chr1	155213809	155213929	121	-	747793_36360010_GBA(2629).8_1	GBA(2629)	CGTGAGCCACCGCGCCCGGCCAACCATCATTATTATTTTTAACGGTAAGGATGGTCAGATTTTACTAATGAAGAAGAGATTATAAAATCTTCAAGTCTTTATATCCACTTGCTTTTTGAG	747793	38.33	Intronic
chr1	155209873	155209993	121	-	747793_36360006_GBA(2629).4_1	GBA(2629)	TGTGAGCCCCTGCGCCCGGCCAAGGGGTGAGGAATTTTGAAACCGTGTTCAGTCTCTCCTAGCAGATGTGTCCATTCTCCATGTCTTCATCAGACCTCACTCTGCTTGTACTCCCTCCCT	747793	54.17	Intronic
chr1	155206939	155207057	119	-	747793_36360004_GBA(2629).2_5	GBA(2629)	GCGATTTCTCTACCCAACTATGGATTCCTAGAGCACCATTCCCCTGGACCTCCAGGGTGCCATGGATCCCACAGTTGTCGCTTGAAACCTTTCTAGGGGCTGGGCGAGGTGGCTCACT	747793	55.93	Intronic

chr1	155210536	155210656	121	-	747793_36360007_GBA(2629).5_13	GBA(2629)	GAGCTGCACCCAGGTTTCTGTGGGCCTTGTCCTAATGAATGTGGGAGACCGGGCCATGGGCACCCAAAGGCAGCTAAGCCCTGCCCAGGAGAGTAGTTGAGGGGTGGAGAGGGGCTTGCT	747793	60.83	Intronic
chr1	155205682	155205802	121	-	747793_36360003_GBA(2629).1_6	GBA(2629)	TTGAGATGCCTGGATCTTCACACCCCCAACTCCTTAGCTACTAAGGAATGTGCCCCTCACAGGGCTGACCTACCCACAGCTGCCTCTCCCACATGTGACCCTTACCTACACTCTCTGGGG	747793	55.83	Intronic
chr1	155211676	155211796	121	-	747793_36360007_GBA(2629).5_3	GBA(2629)	ACAGCTTCCTCCTTGTGTCCACTGAGGATATGGCTTTGTACAACACTTTGGTTTTTGAACGACTTTACAAACCTCCCTGTCTTGTGAGGAAGGAAGAACAGTTATTACCATCTGCATCTG	747793	43.33	Intronic
chr1	155211904	155212024	121	-	747793_36360007_GBA(2629).5_1	GBA(2629)	AACAACAACAAAAAATACAAACAAGAGACAAGTAGTTCCCAGGTGCCTACCAAGTGGTCAGGCACTGCACTTACCTCACTGACTGCAGTAACCACCCTTTGAGGTTGTGGCATTGCCTCC	747793	47.5	Intronic
chr1	155208520	155208640	121	-	747793_36360005_GBA(2629).3_2	GBA(2629)	ATCATCTGCTAACTGCTACGGACTCAGGCTCAGAAAGGCCTGCGCTTCACCCAGGTGCCAGCCTCCACAGGTTCCAACCCAGGAGCCCAAGTTCCC	747793	56.67	Intronic

							TTTGGCCCTGACTCAGACACTATT			
chr1	155211562	155211682	121	-	747793_36360007_GBA(2629).5_4	GBA(2629)	CATCTGATGATGAAACAAGGGACGCTGCAGAGGAGCCGCACTGACCACTCCCTCCCTCCAGTCCTGTCATCCCACTGCCAGTGTCCCACCCTCTTGTGCCCTGCACTTCACTGGCTAATA	747793	57.5	Intronic
chr1	155205202	155205322	121	-	747793_36360003_GBA(2629).1_10	GBA(2629)	GCAGGAAGTGACTAGGTAGCAACAGAAAACCCCAATGCCTGAGGCTGGACTGCGATGCAGAAAAGCAGGGTCAGTGCCCAGCAGCATGGCTCCAGGCCTAGAGAGCCAGGGCAGAGCCTC	747793	59.17	Intronic
chr1	155212285	155212405	121	-	747793_36360008_GBA(2629).6_2	GBA(2629)	GGAGGAACCTAGAAGAAGAAATGATCAATTTTGCTTGGAGTGTATCTAGAAAGACTTCACTGAGATCATTTAAAGAACAAAAAGGATGGCTGGGGTCCAGCGCAGTGGCTCATGCCTGTA	747793	43.33	Intronic
chr1	155211106	155211226	121	-	747793_36360007_GBA(2629).5_8	GBA(2629)	AAATCTGTGTTCTAGGCTCTTCCTAAAGTTGTCACCCATACATGCCCTCCAGAGTTTTATAGGGCATATAATCTGTAACAGATGAGAGGAAGCCAATTGCCCTTTAGAAATATGGCTGTG	747793	41.67	Intronic

chr1	155210650	155210770	121	-	747793_36360007_GBA(2629).5_12	GBA(2629)	TTTGAGCAAGGCACAGATGGAGGGAGGGGTCGAAGTTGAAATGGGTGGGAAGAGTGGTGGGGAGCATCCTGATTTGGGGTGGGCAGAGAGTTGTCATCAGAAGGGTTGCAGGGAGAGCTG	747793	55.83	Intronic
chr1	155206282	155206402	121	-	747793_36360003_GBA(2629).1_1	GBA(2629)	TGTCTCAAAAACAAAAAAAAATCTCCCCAAACCTCTCTAGTTGCATTCTTCCCGTCACCCAACTCCAGGATTCCTACAACAGGAACTAGAAGTTCCAGAAGCCTGTGTGCAAGGTCCAGG	747793	45.83	Intronic
chr1	155205322	155205442	121	-	747793_36360003_GBA(2629).1_9	GBA(2629)	TACCAGGCCTATCATCTCCTACATCGGATGGCTTACATCACTCTACACCACGAGGGAGCAGGAAGGTGTTCAGGGTGGAACCTCGGAAGAGGCACACCCATCCCCTTTTGCACCATGGAG	747793	55	Intronic
chr1	155211220	155211340	121	-	747793_36360007_GBA(2629).5_7	GBA(2629)	TTTAAATTATATCAGCTTTATTTGTACCTTTTTGACATTTCTATCAAAAAAGAAGTGTGCCTGCTGTGGTTCCCATCCTCTGGGATTTAGGAGCCTCTACCCCATTCTCCATGCAAATCT	747793	38.33	Intronic
chr1	155206162	155206282	121	-	747793_36360003_GBA(2629).1_2	GBA(2629)	ATCAGTTGCTCTTCCTTTGCAGGTACTGACAGACCCAGAAGCAGCTAAATATGTTCATGGCATTGCTGTACATTGGTACCTGGACTTTCTGGCTCCAG	747793	50	Exonic

							CCAAAGCCACCCTAGGGGAGAC			
chr1	155206042	155206162	121	-	747793_36360003_GBA(2629).1_3	GBA(2629)	ACACCGCCTGTTCCCCAACACCATGCTCTTTGCCTCAGAGGCCTGTGTGGGCTCCAAGTTCTGGGAGCAGAGTGTGCGGCTAGGCTCCTGGGATCGAGGGATGCAGTACAGCCACAGCAT	747793	60	Exonic
chr1	155205922	155206042	121	-	747793_36360003_GBA(2629).1_4	GBA(2629)	CATCACGGTAAGCCACCCCAGTCTCCCTTCCTGCAAAGCAGACCTCAGACCTCTTACTAGTTTCACCAAAGACTGACAGAAGCCCTTCCTGTCCAGCTTTCCCCAGCTAGCCTGCCCTTT	747793	54.17	Exonic
chr1	155205562	155205682	121	-	747793_36360003_GBA(2629).1_7	GBA(2629)	ACCCCCAGTGTTGCGCCTTTGTCTCTTTGCCTTTGTCCTTACCCTAGAACCTCCTGTACCATGTGGTCGGCTGGACCGACTGGAACCTTGCCCTGAACCCCGAAGGAGGACCCAATTGGG	747793	57.5	Exonic
chr1	155205442	155205562	121	-	747793_36360003_GBA(2629).1_8	GBA(2629)	TGCGTAACTTTGTCGACAGTCCCATCATTGTAGACATCACCAAGGACACGTTTTACAAACAGCCCATGTTCTACCACCTTGGCCACTTCAGGTGAGTGGAGGGCGGGCACCCCCATTCCA	747793	52.5	Exonic

chr1	155205082	155205202	121	-	747793_36360003_GBA(2629).1_11	GBA(2629)	TGCAGGAGTTATGGGGTGGGTCCGTGGGTGGGTGACTTCTTAGATGAGGGTTTCATGGGAGGTACCCCGAGGGACTCTGACCATCTGTTCCCACATTCAGCAAGTTCATTCCTGAGGGCT	747793	55	Exonic
chr1	155204962	155205082	121	-	747793_36360003_GBA(2629).1_12	GBA(2629)	CCCAGAGAGTGGGGCTGGTTGCCAGTCAGAAGAACGACCTGGACGCAGTGGCACTGATGCATCCCGATGGCTCTGCTGTTGTGGTCGTGCTAAACCGGTGAGGGCAATGGTGAGGTCTGG	747793	60	Exonic
chr1	155204842	155204962	121	-	747793_36360003_GBA(2629).1_13	GBA(2629)	GAAGTGGGCTGAAGACAGCGTTGGGGGCCTTGGCAGGATCACACTCTCAGCTTCTCCTCCCTGCTCCCTAGCTCCTCTAAGGATGTGCCTCTTACCATCAAGGATCCTGCTGTGGGCTTC	747793	57.5	Exonic
chr1	155204722	155204842	121	-	747793_36360003_GBA(2629).1_14	GBA(2629)	CTGGAGACAATCTCACCTGGCTACTCCATTCACACCTACCTGTGGCGTCGCCAGTGATGGAGCAGATACTCAAGGAGGCACTGGGCTCAGCCTGGGCATTAAAGGGACAGAGTCAGCTCA	747793	55.83	Exonic
chr1	155204602	155204722	121	-	747793_36360003_GBA(2629).1_15	GBA(2629)	CACGCTGTCTGTGACTAAAGAGGGCACAGCAGGGCCAGTGTGAGCTTACAGCGACGTAAGCCCAGGGGCAATGGTTTGGGTGACTCACTTTCCC	747793	60	Exonic

							CTCTAGGTGGTGCCAGGGGCTGGAGG			
chr1	155204482	155204602	121	-	747793_36360003_GBA(2629).1_16	GBA(2629)	CCCCTAGAAAAAGATCAGTAAGCCCCAGTGTCCCCCCAGCCCCCATGCTTATGTGAACATGCGCTGTGTGCTGCTTGCTTTGGAAACTGGGCCTGGGTCCAGGCCTAGGGTGAGCTCACT	747793	57.5	Exonic
chr1	155204362	155204482	121	-	747793_36360003_GBA(2629).1_17	GBA(2629)	GTCCGTACAAACACAAGATCAGGGCTGAGGGTAAGGAAAAGAAGAGACTAGGAAAGCTGGGCCCAAAACTGGAGACTGTTTGTCTTTCCTGGAGATGCAGAACTGGGCCCGTGGAGCAGC	747793	52.5	Exonic
chr1	155204242	155204362	121	-	747793_36360003_GBA(2629).1_18	GBA(2629)	AGTGTCAGCATCAGGGCGGAAGCCTTAAAGCAGCAGCGGGTGTGCCCAGGCACCCAGATGATTCCTATGGCACCAGCCAGGAAAAATGGCAGCTCTTAAAGGAGAAAATGTTTGAGCCCA	747793	53.33	Exonic
chr1	155204122	155204242	121	-	747793_36360003_GBA(2629).1_19	GBA(2629)	GTCAGTGTGAGTGGCTTTATTCTGGGTGGCAGCACCCCGTGTCCGGCTGTACCAACAACGAGGAGGCACGGGGGCCTCTGGAATGCATGAGAGTAGAAAAACCAGTCTTGGGAGCGTGAG	747793	57.5	Exonic

chr1	155207321	155207441	121	-	747793_36360004_GBA(2629).2_1	GBA(2629)	GTGTGAGCCACCACACCCAGCTGGTCTGGTCCACTTTCTTGGCCGGATCATTCATGACCTTTCTCTTGCCAGGTTCCTGGATGCCTATGCTGAGCACAAGTTACAGTTCTGGGCAGTGAC	747793	54.17	Exonic
chr1	155207225	155207345	121	-	747793_36360004_GBA(2629).2_2	GBA(2629)	CAAGTTACAGTTCTGGGCAGTGACAGCTGAAAATGAGCCTTCTGCTGGGCTGTTGAGTGGATACCCCTTCCAGTGCCTGGGCTTCACCCCTGAACATCAGCGAGACTTCATTGCCCGTGA	747793	54.17	Exonic
chr1	155207129	155207249	121	-	747793_36360004_GBA(2629).2_3	GBA(2629)	TCAGCGAGACTTCATTGCCCGTGACCTAGGTCCTACCCTCGCCAACAGTACTCACCACAATGTCCGCCTACTCATGCTGGATGACCAACGCTTGCTGCTGCCCCACTGGGCAAAGGTGGT	747793	57.5	Exonic
chr1	155207033	155207153	121	-	747793_36360004_GBA(2629).2_4	GBA(2629)	GCTGCCCCACTGGGCAAAGGTGGTAAGGCCTGGACCTCCATGGTGCTCCAGTGACCTTCAAATCCAGCATCCAAATGACTGGCTCCCAAACTTAGAGCGATTTCTCTACCCAACTATGGA	747793	53.33	Exonic
chr1	155208403	155208523	121	-	747793_36360005_GBA(2629).3_3	GBA(2629)	ATTAGGACTGGCAAGTGATAAGCAGAGTCCCATACTCTCCTATTGACTCGGACTACCATATCTTGATCATCCTTTTCTGTAGGAATCGGATATAACATCA	747793	45.83	Exonic

							TCCGGGTACCCATGGCCAGC			
chr1	155208286	155208406	121	-	747793_36360005_GBA(2629).3_4	GBA(2629)	AGCTGTGACTTCTCCATCCGCACCTACACCTATGCAGACACCCCTGATGATTTCCAGTTGCACAACTTCAGCCTCCCAGAGGAAGATACCAAGCTCAAGGTAGGCATTCTAGCTTTTTCA	747793	49.17	Exonic
chr1	155208052	155208172	121	-	747793_36360005_GBA(2629).3_6	GBA(2629)	TGAACCGGATGCACTGGTTGGGCTAGTATGTGTTCCAACTCTGGGTGCTTCTCTCTTCACTACCTTTGTCTCTAGATACCCCTGATTCACCGAGCCCTGCAGTTGGCCCAGCGTCCCGTT	747793	54.17	Exonic
chr1	155207935	155208055	121	-	747793_36360005_GBA(2629).3_7	GBA(2629)	GTTTCACTCCTTGCCAGCCCCTGGACATCACCCACTTGGCTCAAGACCAATGGAGCGGTGAATGGGAAGGGGTCACTCAAGGGACAGCCCGGAGACATCTACCACCAGACCTGGGCCAGA	747793	59.17	Exonic
chr1	155207818	155207938	121	-	747793_36360005_GBA(2629).3_8	GBA(2629)	AGATACTTTGTGAAGTAAGGGATCAGCAAGGATGTGGGATCAGGACTGGCCTCCCATTTAGCCATGCTGATCTGTGTCCCAACCCTCAACCTAGTTCCACTTCCAGATCTGCCTGTCCTC	747793	50.83	Exonic

chr1	155209767	155209887	121	-	747793_36360006_GBA(2629).4_2	GBA(2629)	TGTACTCCCTCCCTCCCAGGTGCCCGCCCCTGCATCCCTAAAAGCTTCGGCTACAGCTCGGTGGTGTGTGTCTGCAATGCCACATACTGTGACTCCTTTGACCCCCCGACCTTTCCTGCC	747793	60	Exonic
chr1	155209661	155209781	121	-	747793_36360006_GBA(2629).4_3	GBA(2629)	CGACCTTTCCTGCCCTTGGTACCTTCAGCCGCTATGAGAGTACACGCAGTGGGCGACGGATGGAGCTGAGTATGGGGCCCATCCAGGCTAATCACACGGGCACAGGTAACCATTACACCC	747793	58.33	Exonic
chr1	155209449	155209569	121	-	747793_36360006_GBA(2629).4_5	GBA(2629)	CTGTGGATGTCCTCAGGCCTGCTACTGACCCTGCAGCCAGAACAGAAGTTCCAGAAAGTGAAGGGATTTGGAGGGGCCATGACAGATGCTGCTGCTCTCAACATCCTTGCCCTGTCACCC	747793	55.83	Exonic
chr1	155209343	155209463	121	-	747793_36360006_GBA(2629).4_6	GBA(2629)	TTGCCCTGTCACCCCCTGCCCAAAATTTGCTACTTAAATCGTACTTCTCTGAAGAAGGTGAGGAGGAAGGGGACAAGATGACATAGAGCCATTGAAACTTTTCGTTTTTCTTTTCTTTTT	747793	41.67	Exonic
chr1	155210992	155211112	121	-	747793_36360007_GBA(2629).5_9	GBA(2629)	GCTGTGATTGCCTCACTTCCTGTGTCATGTGACGCTCCTAGTCATCACATGACCCATCCACATCGGGAAGCCGGAATTACTTGCAGGGCTAACCTAGT	747793	52.5	Exonic

							GCCTATAGCTAAGGCAGGTACC			
chr1	155210878	155210998	121	-	747793_36360007_GBA(2629).5_10	GBA(2629)	GGTACCTGCATCCTTGTTTTTGTTTAGTGGATCCTCTATCCTTCAGAGACTCTGGAACCCCTGTGGTCTTCTCTTCATCTAATGACCCTGAGGGGATGGAGTTTTCAAGTCCTTCCAGAG	747793	47.5	Exonic
chr1	155210764	155210884	121	-	747793_36360007_GBA(2629).5_11	GBA(2629)	CCAGAGAGGTAAGAGAGAGAGCTCCCAATCAGCATTGTCACAGTGCTTCTGGAATCCTGGCACTGGAATTTAATGAATGACAGACTCTCTTTGAATCCAGGGCCATCATGGCTCTTTGAG	747793	47.5	Exonic
chr1	155210422	155210542	121	-	747793_36360007_GBA(2629).5_14	GBA(2629)	CTTGCTTTTCAGTCATTCCTCATTCTGTCCTCAGGAATGTCCCAAGCCTTTGAGTAGGGTAAGCATCATGGCTGGCAGCCTCACAGGATTGCTTCTACTTCAGGCAGTGTCGTGGGCATC	747793	50.83	Exonic
chr1	155210308	155210428	121	-	747793_36360007_GBA(2629).5_15	GBA(2629)	GGCATCAGGTGAGTGAGTCAAGGCAGTGGGGAGGTAGCACAGAGCCTCCCTTCTGCCTCATAGTCCTTTGGTAGCCTTCCAGTAAGCTGGTGGTAGACTTTTAGTAGGTGCTCAATAAAT	747793	50.83	Exonic

chr1	155214477	155214597	121	-	747793_36360011_GBA(2629).9_1	GBA(2629)	CTCTCGCTCTCTCTCTCTCTCCGGCTCGCCAGCGACACTTGTTCGTTCAACTTGACCAATGAGACTTGAGGAAGGGCTCTGAGTCCCGCCTCTGCATGAGTGACCGTCTCTTTTCCAATC	747793	55	Exonic
chr1	155214385	155214505	121	-	747793_36360011_GBA(2629).9_2	GBA(2629)	TGCATGAGTGACCGTCTCTTTTCCAATCCAGGTCCCGCCCCGACTCCCCAGGGCTGCTTTTCTCGCGGCTGCGGGTGGTCGGGCTGCATCCTGCCTTCAGAGTCTTACTGCGCGGGGCCC	747793	65	Exonic
chr1	155214293	155214413	121	-	747793_36360011_GBA(2629).9_3	GBA(2629)	GCCTTCAGAGTCTTACTGCGCGGGGCCCCAGTCTCCAGTCCCGCCCAGGCGCCTTTGCAGGCTGCGGTGGGATTTCGTTTTGCCTCCGGTTGGGGCTGCTGTTTCTCTTCGCCGACGGTA	747793	64.17	Exonic
chr1	155214201	155214321	121	-	747793_36360011_GBA(2629).9_4	GBA(2629)	GGGCTGCTGTTTCTCTTCGCCGACGGTAGGCGTAATGAATATTTCGACCTTTGGATCTTAGCTGTCCCCTCCCTGCGTTCGCACTTAACCTTTTTCACCATTATTATTATTATTGTTATT	747793	43.33	Exonic
chr1	155214645	155214764	120	-	747793_36360012_GBA(2629).10_1	GBA(2629)	TCGGTGGGCTGGACTCACTGTGGGAATTCAATCGCCCCCATCCACCAACAGTGTGCTGGCGGGAAAACGCCGACACGCATGCGTAGTTCTCGCG	747793	60.5	Exonic

							CCGGCTCCTCTCTCTCTCT CTCTCT			

Supplementary Table 2: Probes used to capture *GBA1* transcripts. Custom designed *GBA1* xGen Lockdown Probes (IDT) were used to specifically select and subsequently amplify any *GBA1* cDNA prior to sequencing.

Capture Reactions

**cDNA Amplification #1**		
Reagent	Volume (uL)	Sequence
NEBNext Single Cell cDNA PCR Master Mix	50	
10x cDNA PCR Primer FW	1	NNNAAGCAGTGGTATCAACGCAGAGTACAT

10x cDNA PCR Primer RV	1	NNNCTACACGACGCTCTTCCGATCT
NEBNext Cell Lysis Buffer	0.5	
H20	2	
cDNA	45.5	

**cDNA Amplification #1 _PCR Program**		
Step	Temp	Time
1	98C	45 seconds
2	98C	10 seconds
3	62C	15 seconds
4	72C	3 minutes
Repeat steps 2-4 for 14 cycles		
5	72C	5 minutes
6	4C	Hold

**Hybridization**		
Reagent	Volume (uL)	Sequence
2X Hybridization Buffer	9.5	
Hybridization Buffer Enhancer	3	
xGen Asym TSO Block	1	GCAATGAAGTCGCAGGGTTGGG/3SpC3/
H2O	1	

1X xGen Lockdown Panels/Probes	4.5	See Supplementary Table 2

**Hybridization program (lid set to 100C)**	
Temp	Time
95C	30 sec
65C	4hr
65C	Hold

**Captured cDNA Amplification #2**		
Reagent	Volume (uL)	Sequence
NEBNext Single Cell cDNA PCR Master Mix	50	
10x cDNA PCR Primer FW	1	NNNAAGCAGTGGTATCAACGCAGAGTACAT
10x cDNA PCR Primer RV	1	NNNCTACACGACGCTCTTCCGATCT
NEBNext Cell Lysis Buffer	0.5	
H20	2	
cDNA	45.5	

**Captured cDNA Amplification #2 _PCR Program**		
Step	Temp	Time
1	98C	45 seconds
2	98C	10 seconds
3	62C	15 seconds
4	72C	3 minutes

Repeat steps 2-4 for 9-12 cycles		
5	72C	5 minutes
6	4C	Hold

Library Preparation

**Biotin Tagging Reaction**		
Reagent	Volume (uL)	Sequence
cDNA template	10ng, xuL	
[Btn]Fwd_3580_partial_read1_defined (10uM)	2	5'-/5Biosg/CAGCACTTGCCTGTCGCTCTATCTTCCTACACGACGCTCTTCCGATCT-3'
Rev_PR2_partial_TSO_defined (10uM)	2	5'-CAGCTTTCTGTTGGTGCTGATATTGCAAGCAGTGGTATCAACGCAGAG-3'
H20	21-x uL	
LongAmp Hot Start Taq 2X Master Mix	25	

Step	Temp	Ramp Rate	Time
1	94C	max	3 minutes
2	94C	max	30 seconds
3	66C down to 58C	0.2C/s	90 seconds
4	65C	max	6 minutes
Repeat steps 2-4 for 4 cycles			
5	65C	max	10 minutes
6	4C	-	Hold

Pull-down PCR	
Reagent	Volume (uL)
cPRM	1
H2O	4
LongAmp Hot Start Taq 2X Master Mix	25
Amplicon-bead conjugate	20

Step	Temp	Ramp Rate	Time
1	94C	max	3 minutes
2	94C	max	15 seconds
3	66C down to 58C	0.2C/s	15 seconds
4	65C	max	6 minutes
Repeat steps 2-4 for 4 cycles			
5	65C	max	10 minutes
6	4C	-	Hold

Supplementary Table 3: 10x ONT capture methods. The ONT capture protocol consisted of an initial cDNA amplification, followed by hybridization with custom probes to capture *GBA1* cDNA. The captured cDNA was then amplified before proceeding to library preparation. During library preparation captured and amplified cDNA underwent biotin tagging and pull-down PCR before sequencing.

chr	start	end	region
chr1	155235845	155235885	intron8transcript
chr1	155235681	155235844	exon9
chr1	155236245	155236469	exon8
chr1	155235845	155236244	intron8

chr1	155235886	155236244	intron8_minus_transcrip t
chr1	155234452	155244627	*GBA1*
chr1	155213825	155227534	*GBA1LP*

Supplementary Table 4: Bed file for coverage plots of *GBA1*. Ensembl coordinates were used to assess coverage in various *GBA1* transcript regions including the ~40 bp short transcript region observed in intron 8, both exons 8 and 9, intron 8; intron 8 excluding the ~40 bp short transcript region, and lastly both *GBA1* and *GBA1LP*.

SampleID	Phenotype_PD	Sex	Age at Baseline	HapMap_Ancestry	rs3115534	COHORT
PD-PDAW035PFW	Control	Female	35	AFRICA	TT	PDBP
PD-PDMP314UYR	Control	Male	52	EUROPE	TT	PDBP
PD-PDPG365GUX	Control	Female	58	EUROPE	TT	PDBP
PP-4054	Case	Female	62	AFRICA	GT	PPMI

PD-PDAD058WN2	Control	Male	58	EUROPE	TT	PDBP
PD-PDAJ625BEE	Case	Male	77	EUROPE	TT	PDBP
PD-PDAT617NNK	Case	Male	51	EUROPE	TT	PDBP
PD-PDAZ595YFB	Control	Male	57	EUROPE	TT	PDBP
PD-PDBN442LGY	Case	Male	58	EUROPE	GT	PDBP
PD-PDBT843RR4	Control	Male	79	EUROPE	TT	PDBP
PD-PDCC802BWZ	Case	Male	80	EUROPE	TT	PDBP
PD-PDCF366ZNK	Case	Male	65	ADMIX	TT	PDBP
PD-PDDG639EDV	Control	Female	75	EUROPE	TT	PDBP
PD-PDDR208XUQ	Case	Male	58	EUROPE	TT	PDBP
PD-PDEA596AUU	Case	Female	55	EUROPE	TT	PDBP
PD-PDED633MRU	Control	Female	68	AFRICA	TT	PDBP
PD-PDEL817NAR	Case	Male	42	AFRICA	GT	PDBP
PD-PDEZ752YGZ	Case	Male	79	EUROPE	TT	PDBP
PD-PDFC083TPY	Control	Female	57	AFRICA	TT	PDBP
PD-PDFH598YPL	Case	Male	60	EUROPE	TT	PDBP
PD-PDFK195KHK	Control	Male	79	EUROPE	GT	PDBP
PD-PDFP184NXJ	Case	Male	69	EUROPE	TT	PDBP
PD-PDFU123GRM	Control	Female	68	EUROPE	TT	PDBP
PD-PDFV321TNV	Case	Male	58	EUROPE	TT	PDBP
PD-PDGC177LBJ	Case	Male	53	ADMIX	TT	PDBP
PD-PDGL166MPM	Case	Male	59	EUROPE	TT	PDBP
PD-PDGL666TUD	Case	Male	60	EUROPE	GT	PDBP
PD-PDHR901EH2	Control	Male	70	EUROPE	GT	PDBP
PD-PDHT199DNF	Control	Female	79	EUROPE	TT	PDBP

PD-PDHY086NRC	Case	Male	61	ADMIX	TT	PDBP
PD-PDJG352DVL	Control	Male	64	EUROPE	TT	PDBP
PD-PDJR209DL5	Case	Male	76	EUROPE	TT	PDBP
PD-PDKC771CTD	Case	Female	61	EUROPE	TT	PDBP
PD-PDKF565VCJ	Case	Male	53	EUROPE	TT	PDBP
PD-PDKJ872VYL	Case	Female	67	AFRICA	TT	PDBP
PD-PDKU919CLW	Case	Male	74	EUROPE	GT	PDBP
PD-PDLF034LZ7	Case	Male	61	EUROPE	GT	PDBP
PD-PDLN584XE1	Control	Female	52	EUROPE	TT	PDBP
PD-PDLW898UEJ	Case	Male	72	EUROPE	TT	PDBP
PD-PDMF065VWD	Control	Male	65	EUROPE	TT	PDBP
PD-PDMM387JY7	Case	Female	56	AFRICA	GG	PDBP
PD-PDMM423XZH	Control	Male	79	EUROPE	GT	PDBP
PD-PDMW916MCX	Case	Male	48	EUROPE	GT	PDBP
PD-PDMX357NA5	Case	Male	38	EUROPE	TT	PDBP
PD-PDMY333PTR	Case	Female	58	EUROPE	GT	PDBP
PD-PDNB017RBH	Case	Female	64	AFRICA	GT	PDBP
PD-PDNB563CBA	Control	Male	79	EUROPE	GT	PDBP
PD-PDNR621JVA	Control	Male	58	EUROPE	TT	PDBP
PD-PDNT699ZTN	Case	Male	69	EUROPE	GT	PDBP
PD-PDNV525LXN	Case	Male	71	EUROPE	TT	PDBP
PD-PDNZ095VCJ	Control	Male	70	AFRICA	GT	PDBP
PD-PDPJ264VEU	Case	Male	67	EUROPE	GT	PDBP
PD-PDPJ859VT8	Control	Female	53	EUROPE	TT	PDBP
PD-PDPK706ZF3	Case	Male	81	EUROPE	GT	PDBP

PD-PDPY235UBA	Control	Male	57	AFRICA	TT	PDBP
PD-PDRB766CVC	Control	Female	81	EUROPE	TT	PDBP
PD-PDRG028AC6	Control	Female	66	EUROPE	TT	PDBP
PD-PDRT048WTN	Case	Female	46	EUROPE	TT	PDBP
PD-PDRX791LAJ	Case	Male	68	EUROPE	TT	PDBP
PD-PDRZ663BT0	Case	Male	66	EUROPE	TT	PDBP
PD-PDTB167HLE	Control	Female	58	ADMIX	GT	PDBP
PD-PDTL596HLB	Case	Male	68	EUROPE	GT	PDBP
PD-PDTN282KNQ	Control	Male	68	EUROPE	TT	PDBP
PD-PDTU185FY2	Control	Male	64	EUROPE	TT	PDBP
PD-PDUC469GHX	Control	Female	49	ADMIX	GT	PDBP
PD-PDUH411AA3	Case	Female	50	EUROPE	GT	PDBP
PD-PDUR690WXZ	Control	Male	55	EUROPE	TT	PDBP
PD-PDUW668XHZ	Control	Female	70	EUROPE	TT	PDBP
PD-PDVP464PFE	Case	Female	67	EUROPE	TT	PDBP
PD-PDVY964MT7	Case	Female	74	EUROPE	TT	PDBP
PD-PDWD667FYL	Case	Female	77	ADMIX	GT	PDBP
PD-PDXD799FTD	Case	Female	51	ADMIX	GT	PDBP
PD-PDXG651JZH	Control	Male	84	EUROPE	TT	PDBP
PD-PDXH265ZCZ	Control	Female	72	EUROPE	GT	PDBP
PD-PDXM323AVM	Control	Male	51	AFRICA	TT	PDBP
PD-PDXT901PPY	Case	Female	65	EUROPE	TT	PDBP
PD-PDXV913MME	Case	Male	55	EUROPE	TT	PDBP
PD-PDYU466BGL	Case	Female	74	EUROPE	TT	PDBP
PD-PDZF741MB6	Control	Female	69	EUROPE	TT	PDBP

PD-PDZP686RU1	Control	Male	82	EUROPE	GT	PDBP
PD-PDZZ075NW6	Case	Male	64	EUROPE	TT	PDBP
PP-3009	Control	Female	84	AFRICA	TT	PPMI
PP-3069	Control	Female	54	EUROPE	TT	PPMI
PP-3072	Control	Female	47	EUROPE	TT	PPMI
PP-3106	Control	Female	70	EUROPE	TT	PPMI
PP-3160	Case	Male	79	EUROPE	GT	PPMI
PP-3178	Case	Male	72	ASIA	TT	PPMI
PP-3189	Case	Female	71	EUROPE	TT	PPMI
PP-3227	Case	Female	51	EUROPE	TT	PPMI
PP-3231	Case	Female	56	EUROPE	GT	PPMI
PP-3253	Case	Female	42	ADMIX	GT	PPMI
PP-3305	Case	Male	65	EUROPE	TT	PPMI
PP-3357	Control	Male	44	AFRICA	TT	PPMI
PP-3375	Case	Male	50	ADMIX	TT	PPMI
PP-3420	Case	Male	51	EUROPE	TT	PPMI
PP-3501	Case	Male	46	ADMIX	GT	PPMI
PP-3537	Case	Female	42	EUROPE	TT	PPMI
PP-3569	Control	Female	40	AFRICA	TT	PPMI
PP-3631	Case	Female	68	EUROPE	TT	PPMI
PP-3637	Control	Male	58	EUROPE	TT	PPMI
PP-3662	Control	Male	57	EUROPE	GT	PPMI
PP-3704	Case	Male	62	EUROPE	TT	PPMI
PP-4018	Control	Male	64	AFRICA	GT	PPMI
PP-4032	Control	Male	67	EUROPE	TT	PPMI

PP-4036	Control	Female	50	EUROPE	GT	PPMI
PP-40553	Case	Female	69	EUROPE	TT	PPMI
PP-40759	Control	Female	44	EUROPE	TT	PPMI
PP-4082	Case	Male	65	EUROPE	TT	PPMI
PP-4111	Case	Male	74	EUROPE	GT	PPMI
PP-4114	Case	Male	67	ADMIX	GT	PPMI
PP-41285	Case	Female	68	EUROPE	TT	PPMI
PP-41303	Case	Female	68	EUROPE	GT	PPMI
PP-41382	Case	Male	59	EUROPE	TT	PPMI
PP-41430	Case	Male	50	EUROPE	TT	PPMI
PP-41467	Case	Female	73	EUROPE	GT	PPMI
PP-41519	Case	Female	71	EUROPE	TT	PPMI
PP-41523	Control	Female	51	EUROPE	TT	PPMI
PP-41984	Control	Female	62	EUROPE	GT	PPMI
PP-41993	Control	Male	68	EUROPE	GT	PPMI
PP-42418	Case	Female	68	EUROPE	TT	PPMI
PP-42446	Control	Male	74	EUROPE	GT	PPMI
PP-50027	Case	Male	64	EUROPE	GT	PPMI
PP-50110	Control	Male	56	EUROPE	TT	PPMI
PP-50172	Control	Female	65	EUROPE	TT	PPMI
PP-50961	Control	Male	68	EUROPE	TT	PPMI
PP-51519	Control	Female	29	EUROPE	TT	PPMI
PP-51538	Control	Female	68	EUROPE	GT	PPMI
PP-51556	Case	Female	60	EUROPE	GT	PPMI
PP-51670	Case	Female	71	EUROPE	TT	PPMI

PP-51686	Control	Female	57	EUROPE	GT	PPMI
PP-51918	Control	Male	75	EUROPE	TT	PPMI
PP-52037	Control	Male	55	EUROPE	TT	PPMI
PP-52565	Control	Male	26	EUROPE	TT	PPMI
PP-54309	Control	Male	72	EUROPE	TT	PPMI
PP-54663	Control	Female	48	EUROPE	GT	PPMI
PP-55380	Control	Female	66	EUROPE	TT	PPMI
PP-55726	Control	Female	33	EUROPE	TT	PPMI
PP-57159	Control	Male	56	EUROPE	GT	PPMI
PP-57365	Control	Male	41	EUROPE	TT	PPMI
PP-57714	Control	Female	58	EUROPE	TT	PPMI
PP-58224	Control	Female	60	EUROPE	TT	PPMI
PP-59343	Control	Female	46	ADMIX	TT	PPMI
PP-59398	Case	Male	67	EUROPE	GT	PPMI
PP-59926	Control	Male	47	EUROPE	GT	PPMI
PP-60048	Case	Female	74	EUROPE	TT	PPMI
PP-60101	Case	Male	67	EUROPE	TT	PPMI

Supplementary Table 5: AMP-PD samples used for coverage information. Data was accessed through the AMP-PD platform for WGS and RNAseq. Individual sample identifiers, case-control status, sex, predicted ancestry, rs3115534 variant status and cohort or origin are provided.

SampleID	rs3115534	Sex	Ancestry	Population
NA18486	TT	Male	African	Yoruba
NA18487	GT	Male	African	Yoruba
NA18488	GT	Female	African	Yoruba

NA18489	TT	Female	African	Yoruba
NA18498	GG	Male	African	Yoruba
NA18499	TT	Female	African	Yoruba
NA18502	GT	Female	African	Yoruba
NA18505	GT	Female	African	Yoruba
NA18508	TT	Female	African	Yoruba
NA18510	TT	Male	African	Yoruba
NA18511	GT	Female	African	Yoruba
NA18517	TT	Female	African	Yoruba
NA18519	TT	Male	African	Yoruba
NA18520	TT	Female	African	Yoruba
NA18858	GT	Female	African	Yoruba
NA18861	GT	Female	African	Yoruba
NA18867	GT	Female	African	Yoruba
NA18868	TT	Male	African	Yoruba
NA18870	GT	Female	African	Yoruba
NA18873	TT	Female	African	Yoruba
NA18907	TT	Female	African	Yoruba
NA18908	TT	Male	African	Yoruba
NA18909	GT	Female	African	Yoruba
NA18910	GT	Male	African	Yoruba
NA18916	TT	Female	African	Yoruba
NA18917	TT	Male	African	Yoruba
NA18923	TT	Male	African	Yoruba
NA18933	TT	Female	African	Yoruba

NA18934	GT	Male	African	Yoruba
NA19092	GT	Male	African	Yoruba
NA19093	GG	Female	African	Yoruba
NA19095	TT	Female	African	Yoruba
NA19096	TT	Male	African	Yoruba
NA19098	TT	Male	African	Yoruba
NA19099	GT	Female	African	Yoruba
NA19102	GT	Female	African	Yoruba
NA19107	TT	Male	African	Yoruba
NA19108	GG	Female	African	Yoruba
NA19113	GT	Male	African	Yoruba
NA19114	GT	Female	African	Yoruba
NA19116	GT	Female	African	Yoruba
NA19117	GT	Male	African	Yoruba
NA19118	GT	Female	African	Yoruba
NA19119	TT	Male	African	Yoruba
NA19121	GT	Male	African	Yoruba
NA19129	GT	Female	African	Yoruba
NA19130	TT	Male	African	Yoruba
NA19131	GT	Female	African	Yoruba
NA19137	GT	Female	African	Yoruba
NA19138	TT	Male	African	Yoruba
NA19141	GT	Male	African	Yoruba
NA19143	TT	Female	African	Yoruba
NA19144	TT	Male	African	Yoruba

NA19146	TT	Male	African	Yoruba
NA19147	GT	Female	African	Yoruba
NA19149	GT	Female	African	Yoruba
NA19150	TT	Male	African	Yoruba
NA19152	GG	Female	African	Yoruba
NA19153	GT	Male	African	Yoruba
NA19159	TT	Female	African	Yoruba
NA19160	GG	Male	African	Yoruba
NA19171	TT	Male	African	Yoruba
NA19172	TT	Female	African	Yoruba
NA19175	GT	Male	African	Yoruba
NA19184	TT	Male	African	Yoruba
NA19185	GT	Female	African	Yoruba
NA19189	GT	Male	African	Yoruba
NA19190	GT	Female	African	Yoruba
NA19197	GT	Female	African	Yoruba
NA19198	GT	Male	African	Yoruba
NA19200	GT	Male	African	Yoruba
NA19201	GG	Female	African	Yoruba
NA19204	TT	Female	African	Yoruba
NA19206	GT	Female	African	Yoruba
NA19207	TT	Male	African	Yoruba
NA19209	GT	Female	African	Yoruba
NA19210	TT	Male	African	Yoruba
NA19213	TT	Male	African	Yoruba

NA19214	TT	Female	African	Yoruba
NA19222	TT	Female	African	Yoruba
NA19223	TT	Male	African	Yoruba
NA19225	TT	Female	African	Yoruba
NA19235	GT	Female	African	Yoruba
NA19236	TT	Male	African	Yoruba
NA19247	GT	Female	African	Yoruba
NA19248	GG	Male	African	Yoruba
NA19256	TT	Male	African	Yoruba
NA19257	GT	Female	African	Yoruba

Supplementary Table 6: 1000 Genomes samples used for coverage information. Data was accessed through The International Genome Sample Resource platform for WGS and RNAseq. Individual sample identifiers, sex, ancestry and specific population, as well as rs3115534 variant status are provided.

SampleID	Sample Type	Sample Origin	Neurologic diagnosis	rs3115534	Assay Performed	Sex	Age at Sampling
CMC_HBCC_014	Frontal Cortex	HBCC Brain Bank	Control	TT	Illumina RNAseq	Male	22.1
CMC_HBCC_023	Frontal Cortex	HBCC Brain Bank	Control	GG	Illumina RNAseq	Male	18.1

CMC_HBCC_025	Frontal Cortex	HBCC Brain Bank	Control	TT	Illumina RNAseq	Male	18.2
CMC_HBCC_035	Frontal Cortex	HBCC Brain Bank	Control	TT	Illumina RNAseq	Male	42.3
CMC_HBCC_044	Frontal Cortex	HBCC Brain Bank	Control	TT	Illumina RNAseq	Male	41.6
CMC_HBCC_049	Frontal Cortex	HBCC Brain Bank	Control	GT	Illumina RNAseq	Male	35.3
CMC_HBCC_052	Frontal Cortex	HBCC Brain Bank	Control	GT	Illumina RNAseq	Male	21
CMC_HBCC_062	Frontal Cortex	HBCC Brain Bank	Control	TT	Illumina RNAseq	Male	20.9
CMC_HBCC_066	Frontal Cortex	HBCC Brain Bank	Control	TT	Illumina RNAseq	Male	58.5
CMC_HBCC_097	Frontal Cortex	HBCC Brain Bank	Control	TT	Illumina RNAseq	Female	21.5
CMC_HBCC_105	Frontal Cortex	HBCC Brain Bank	Control	TT	Illumina RNAseq	Male	22.2
CMC_HBCC_208	Frontal Cortex	HBCC Brain Bank	Control	TT	Illumina RNAseq	Male	48.5
CMC_HBCC_226	Frontal Cortex	HBCC Brain Bank	Control	TT	Illumina RNAseq	Male	27.2
CMC_HBCC_269	Frontal Cortex	HBCC Brain Bank	Control	TT	Illumina RNAseq	Male	48.6
CMC_HBCC_285	Frontal Cortex	HBCC Brain Bank	Control	GT	Illumina RNAseq	Male	54.9
CMC_HBCC_318	Frontal Cortex	HBCC Brain Bank	Control	TT	Illumina RNAseq	Male	46

CMC_HBCC_320	Frontal Cortex	HBCC Brain Bank	Control	TT	Illumina RNAseq	Female	46.7
CMC_HBCC_362	Frontal Cortex	HBCC Brain Bank	Control	TT	Illumina RNAseq	Male	44.7
CMC_HBCC_412	Frontal Cortex	HBCC Brain Bank	Control	TT	Illumina RNAseq	Female	56.6
CMC_HBCC_418	Frontal Cortex	HBCC Brain Bank	Control	TT	Illumina RNAseq	Male	36.1
CMC_HBCC_457	Frontal Cortex	HBCC Brain Bank	Control	TT	Illumina RNAseq	Female	57.4
CMC_HBCC_RNA_PFC_3054	Frontal Cortex	HBCC Brain Bank	Control	TT	Illumina RNAseq	Male	51.7
CMC_HBCC_RNA_PFC_3059	Frontal Cortex	HBCC Brain Bank	Control	TT	Illumina RNAseq	Male	37.6
CMC_HBCC_RNA_PFC_3064	Frontal Cortex	HBCC Brain Bank	Control	TT	Illumina RNAseq	Female	54.6
CMC_HBCC_RNA_PFC_3067	Frontal Cortex	HBCC Brain Bank	Control	TT	Illumina RNAseq	Female	38.6
CMC_HBCC_RNA_PFC_3071	Frontal Cortex	HBCC Brain Bank	Control	TT	Illumina RNAseq	Male	51.6
CMC_HBCC_RNA_PFC_3072	Frontal Cortex	HBCC Brain Bank	Control	GT	Illumina RNAseq	Male	24.9
CMC_HBCC_RNA_PFC_3075	Frontal Cortex	HBCC Brain Bank	Control	TT	Illumina RNAseq	Male	65.6
CMC_HBCC_RNA_PFC_3076	Frontal Cortex	HBCC Brain Bank	Control	TT	Illumina RNAseq	Male	25.8
CMC_HBCC_RNA_PFC_3080	Frontal Cortex	HBCC Brain Bank	Control	GT	Illumina RNAseq	Male	60.8

CMC_HBCC_RNA_PFC_3081	Frontal Cortex	HBCC Brain Bank	Control	GT	Illumina RNAseq	Female	31.5
CMC_HBCC_RNA_PFC_3085	Frontal Cortex	HBCC Brain Bank	Control	TT	Illumina RNAseq	Male	22.6
CMC_HBCC_RNA_PFC_3087	Frontal Cortex	HBCC Brain Bank	Control	TT	Illumina RNAseq	Male	35.6
CMC_HBCC_RNA_PFC_3088	Frontal Cortex	HBCC Brain Bank	Control	TT	Illumina RNAseq	Male	64.6
CMC_HBCC_RNA_PFC_3093	Frontal Cortex	HBCC Brain Bank	Control	GG	Illumina RNAseq	Female	30
CMC_HBCC_RNA_PFC_3095	Frontal Cortex	HBCC Brain Bank	Control	TT	Illumina RNAseq	Male	42
CMC_HBCC_RNA_PFC_3096	Frontal Cortex	HBCC Brain Bank	Control	TT	Illumina RNAseq	Male	49.8
CMC_HBCC_RNA_PFC_3097	Frontal Cortex	HBCC Brain Bank	Control	GT	Illumina RNAseq	Female	26.8
CMC_HBCC_RNA_PFC_3098	Frontal Cortex	HBCC Brain Bank	Control	TT	Illumina RNAseq	Female	26.2
CMC_HBCC_RNA_PFC_3105	Frontal Cortex	HBCC Brain Bank	Control	TT	Illumina RNAseq	Male	20.2
CMC_HBCC_RNA_PFC_3108	Frontal Cortex	HBCC Brain Bank	Control	TT	Illumina RNAseq	Male	73.9
CMC_HBCC_RNA_PFC_3109	Frontal Cortex	HBCC Brain Bank	Control	GT	Illumina RNAseq	Female	41
CMC_HBCC_RNA_PFC_3110	Frontal Cortex	HBCC Brain Bank	Control	GT	Illumina RNAseq	Female	40.8
CMC_HBCC_RNA_PFC_3111	Frontal Cortex	HBCC Brain Bank	Control	TT	Illumina RNAseq	Male	31.6

CMC_HBCC_RNA_PFC_3116	Frontal Cortex	HBCC Brain Bank	Control	TT	Illumina RNAseq	Female	64.9
CMC_HBCC_RNA_PFC_3119	Frontal Cortex	HBCC Brain Bank	Control	GG	Illumina RNAseq	Female	53.6
CMC_HBCC_RNA_PFC_3132	Frontal Cortex	HBCC Brain Bank	Control	TT	Illumina RNAseq	Female	46.6
CMC_HBCC_RNA_PFC_3136	Frontal Cortex	HBCC Brain Bank	Control	TT	Illumina RNAseq	Female	21.3
CMC_HBCC_RNA_PFC_3137	Frontal Cortex	HBCC Brain Bank	Control	GT	Illumina RNAseq	Female	65
CMC_HBCC_RNA_PFC_3139	Frontal Cortex	HBCC Brain Bank	Control	TT	Illumina RNAseq	Female	71.8
CMC_HBCC_RNA_PFC_3149	Frontal Cortex	HBCC Brain Bank	Control	TT	Illumina RNAseq	Male	53.3
CMC_HBCC_RNA_PFC_3153	Frontal Cortex	HBCC Brain Bank	Control	GT	Illumina RNAseq	Male	43.9
CMC_HBCC_RNA_PFC_3154	Frontal Cortex	HBCC Brain Bank	Control	TT	Illumina RNAseq	Female	30.5
CMC_HBCC_RNA_PFC_3162	Frontal Cortex	HBCC Brain Bank	Control	TT	Illumina RNAseq	Male	85.2
CMC_HBCC_RNA_PFC_3164	Frontal Cortex	HBCC Brain Bank	Control	GT	Illumina RNAseq	Male	58.5
CMC_HBCC_RNA_PFC_3167	Frontal Cortex	HBCC Brain Bank	Control	TT	Illumina RNAseq	Male	48.7
CMC_HBCC_RNA_PFC_3177	Frontal Cortex	HBCC Brain Bank	Control	TT	Illumina RNAseq	Female	52.6
CMC_HBCC_RNA_PFC_3182	Frontal Cortex	HBCC Brain Bank	Control	TT	Illumina RNAseq	Female	53

CMC_HBCC_RNA_PFC_3186	Frontal Cortex	HBCC Brain Bank	Control	TT	Illumina RNAseq	Male	71.4
CMC_HBCC_RNA_PFC_3188	Frontal Cortex	HBCC Brain Bank	Control	TT	Illumina RNAseq	Male	19.4
CMC_HBCC_RNA_PFC_3189	Frontal Cortex	HBCC Brain Bank	Control	TT	Illumina RNAseq	Female	51.5
CMC_HBCC_RNA_PFC_3196	Frontal Cortex	HBCC Brain Bank	Control	TT	Illumina RNAseq	Female	67.8
CMC_HBCC_RNA_PFC_3199	Frontal Cortex	HBCC Brain Bank	Control	TT	Illumina RNAseq	Female	44.8
CMC_HBCC_RNA_PFC_3206	Frontal Cortex	HBCC Brain Bank	Control	TT	Illumina RNAseq	Male	31.8
CMC_HBCC_RNA_PFC_3209	Frontal Cortex	HBCC Brain Bank	Control	TT	Illumina RNAseq	Female	40.2
CMC_HBCC_RNA_PFC_3362	Frontal Cortex	HBCC Brain Bank	Control	TT	Illumina RNAseq	Male	45.3
CMC_HBCC_RNA_PFC_3363	Frontal Cortex	HBCC Brain Bank	Control	GT	Illumina RNAseq	Male	34.8
CMC_HBCC_RNA_PFC_3364	Frontal Cortex	HBCC Brain Bank	Control	GG	Illumina RNAseq	Male	29.1
CMC_HBCC_RNA_PFC_3372	Frontal Cortex	HBCC Brain Bank	Control	TT	Illumina RNAseq	Female	54.5
CMC_HBCC_RNA_PFC_3374	Frontal Cortex	HBCC Brain Bank	Control	TT	Illumina RNAseq	Male	49.7
CMC_HBCC_RNA_PFC_3382	Frontal Cortex	HBCC Brain Bank	Control	TT	Illumina RNAseq	Male	68.6
CMC_HBCC_RNA_PFC_3384	Frontal Cortex	HBCC Brain Bank	Control	GT	Illumina RNAseq	Male	48.6

CMC_HBCC_RNA_PFC_3385	Frontal Cortex	HBCC Brain Bank	Control	TT	Illumina RNAseq	Female	65
CMC_HBCC_RNA_PFC_3386	Frontal Cortex	HBCC Brain Bank	Control	TT	Illumina RNAseq	Female	36.5
CMC_HBCC_RNA_PFC_3387	Frontal Cortex	HBCC Brain Bank	Control	GG	Illumina RNAseq	Male	23.6
CMC_HBCC_RNA_PFC_3391	Frontal Cortex	HBCC Brain Bank	Control	GT	Illumina RNAseq	Female	58
CMC_HBCC_RNA_PFC_3395	Frontal Cortex	HBCC Brain Bank	Control	TT	Illumina RNAseq	Male	51.6
CMC_HBCC_RNA_PFC_3400	Frontal Cortex	HBCC Brain Bank	Control	GT	Illumina RNAseq	Female	45.4
CMC_HBCC_RNA_PFC_3402	Frontal Cortex	HBCC Brain Bank	Control	GT	Illumina RNAseq	Male	23.2
CMC_HBCC_RNA_PFC_3406	Frontal Cortex	HBCC Brain Bank	Control	GT	Illumina RNAseq	Female	48.1
CMC_HBCC_RNA_PFC_3408	Frontal Cortex	HBCC Brain Bank	Control	TT	Illumina RNAseq	Male	45.2
CMC_HBCC_RNA_PFC_3417	Frontal Cortex	HBCC Brain Bank	Control	GT	Illumina RNAseq	Male	50.5
CMC_HBCC_RNA_PFC_3508	Frontal Cortex	HBCC Brain Bank	Control	TT	Illumina RNAseq	Male	84.2
CMC_HBCC_RNA_PFC_3521	Frontal Cortex	HBCC Brain Bank	Control	TT	Illumina RNAseq	Male	54.5
CMC_HBCC_RNA_PFC_3523	Frontal Cortex	HBCC Brain Bank	Control	TT	Illumina RNAseq	Male	24.9
CMC_HBCC_RNA_PFC_3536	Frontal Cortex	HBCC Brain Bank	Control	TT	Illumina RNAseq	Male	27.6

CMC_HBCC_RNA_PFC_3552	Frontal Cortex	HBCC Brain Bank	Control	TT	Illumina RNAseq	Male	65.9
CMC_HBCC_RNA_PFC_3595	Frontal Cortex	HBCC Brain Bank	Control	TT	Illumina RNAseq	Female	54.9
CMC_HBCC_RNA_PFC_3601	Frontal Cortex	HBCC Brain Bank	Control	TT	Illumina RNAseq	Male	50.6
CMC_HBCC_RNA_PFC_3883	Frontal Cortex	HBCC Brain Bank	Control	GT	Illumina RNAseq	Female	40.3
CMC_HBCC_RNA_PFC_3888	Frontal Cortex	HBCC Brain Bank	Control	GG	Illumina RNAseq	Male	40.3
CMC_HBCC_RNA_PFC_3909	Frontal Cortex	HBCC Brain Bank	Control	TT	Illumina RNAseq	Male	56.3

Supplementary Table 7: HBCC Illumina RNAseq samples used for coverage information. Data was accessed through the Human Brain Collection Core for Illumina RNAseq data. Individual sample identifiers, disease status, and rs3115534 variant status are provided.

Primers	Sequence	Name
Forward	GCGACGCCACAGGTAG	GBA1_X11_F9
Reverse	CTTTGTCCTTACCCTAGAACCTC	GBA1_X11_R9
Reversed	CCTTTGTCCTTACCCTAGAACC	GBA1_UTR_R4

PCR Conditions			
**Stage**	**Temperature**	**Time**	
Initial Denaturation	95°C	4:00	
Denaturation	95°C	0:30	40x cycles
Annealing	65°C (-0.3°C/cycle)	1:00	

Extension	72°C	1:00	
Final Extension	72°C	10:00	
Hold	4°C	Indefinite	

Supplementary Table 8: Reverse transcription PCR Primers and Conditions. Primers 9F, 9R and 4R were selected after initial optimization–respective sequences are provided. In an effort to increase specificity a touchdown PCR method was used as detailed in the PCR conditions.

**Sample (genotype, band, primer)**	**BLAST Top Hit**	**Selected Sequence**	**Selected BP**	**Complete Sequence**	**Complete BP**
GG Top 9F	Homo sapiens glucosylceramidase beta 1 (*GBA1*), transcript variant 3, mRNA	GGAAGCCCACAGCAGGATCCTTGATGGTAAGAGGCACATCCTTAGAGGAGCGGTTTAGGACGACCACAACAGCAGAGCCATCGGGATGCATCAGTGCCACTGCGTCCGGGTCGTTCTTCTGACTGGCAACCAGCCCCACTCTCTGGGAGCCCTCAGGAATGAACTTGCTGAAGTGGCCAAGGTGGTAGAACATGGGCTGTTTGTAAAACGTGTCCTTGGTGATGTCTACAATGATGGGACTGTCGACAAAGTTACGCACCCAATTGGGTCCTCCTTCGGGGTTCAGGGCAAAGTTCCAGTCGGTCCAGCCGACCACATGGTACAGGAGGTTCTAGGGTAAGGACA	345	NNNNNNNNNNGNNAGANTGTCTCCNGGAAGCCCACAGCAGGATCCTTGATGGTAAGAGGCACATCCTTAGAGGAGCGGTTTAGGACGACCACAACAGCAGAGCCATCGGGATGCATCAGTGCCACTGCGTCCGGGTCGTTCTTCTGACTGGCAACCAGCCCCACTCTCTGGGAGCCCTCAGGAATGAACTTGCTGAAGTGGCCAAGGTGGTAGAACATGGGCTGTTTGTAAAACGTGTCCTTGGTGATGTCTACAATGATGGGACTGTCGACAAAGTTACGCACCCAATTGGGTCCTCCTTCGGGGTTCAGGGCAAAGTTCCAGTCGGTCCAGCCGACCACATGGTACAGGAGGTTCTAGGGTAAGGACAAAA	373

GG Top 9R	Homo sapiens glucosylceramidase beta 1 (*GBA1*), transcript variant 3, mRNA	CATTGTACACATCACCAAGGAC<br>ACGTTTTACAAACAGCCCATGT<br>TCTACCACCTTGGCCACTTCAG<br>CAAGTTCATTCCTGAGGGCTCC<br>CACATAGTGGGGCTGGTTGCCA<br>TTCAGAAGAACGACCCGGACC<br>CACTGGCACTGATGCATCCCAA<br>TGGCTCTGCTGTTGTGGTCGTG<br>CTAAACCGCTCCTCTAAGGATG<br>TGCCTCTTACCATCAAGGATCC<br>TGCTGTGGGCATCCTGGAGAC<br>AATCTCACCTGGCTACTCCATTC<br>ACACCTACCTGTGGCGT	280	NNNNNGNNTCNGNNNGACC<br>GACTGGNNNCTTGCCCTGA<br>ACCCCGAAGGAGGACCCAA<br>TTGGGTGCGTAACTTTGTCC<br>ACAGTCCCATCATTGTACAC<br>ATCACCAAGGACACGTTTTA<br>CAAACAGCCCATGTTCTACC<br>ACCTTGGCCACTTCAGCAA<br>GTTCATTCCTGAGGGCTCC<br>CACATAGTGGGGCTGGTTG<br>CCATTCAGAAGAACGACCC<br>GGACCCACTGGCACTGATG<br>CATCCCAATGGCTCTGCTGT<br>TGTGGTCGTGCTAAACCGC<br>TCCTCTAAGGATGTGCCTCT<br>TACCATCAAGGATCCTGCTG<br>TGGGCATCCTGGAGACAAT<br>CTCACCTGGCTACTCCATTC<br>ACACCTACCTGTGGCGTCG<br>CCAN	373
GG Bottom 9F	Human ORFeome Gateway entry vector pENTR223-*GBAP1*, complete sequence	GGAAGCCCACAGCAGGATCCT<br>TGATGGTAAGAGGCACATCCTT<br>AGAGGAGCGGTTTNGGNCGAC<br>CACAACAGGAGAGCCATCGGG<br>ATGCATCAGTGCCACTGCGTCC<br>GGGTCGTTCTTCTGACTGGCAA<br>CCAGCCCCACTCTCTGGGAGC<br>CCTCAGGAATGAACTTGCTGAA<br>GTGGCCAAGGTGGTAGAACAT<br>GGGCTGTTTGTAAAACGTGTGC<br>TTGGTGATGTCTACAATGATGG<br>GTTCCAGTCGGTCCAGCCGAC<br>CACATGGTACAGGAGGTTCTAG<br>GGTAAGGACAAA	292	NNNNNNNNGNNANANTGNC<br>TCCNGGAAGCCCACAGCAG<br>GATCCTTGATGGTAAGAGG<br>CACATCCTTAGAGGAGCGG<br>TTTNGGNCGACCACAACAG<br>GAGAGCCATCGGGATGCAT<br>CAGTGCCACTGCGTCCGGG<br>TCGTTCTTCTGACTGGCAAC<br>CAGCCCCACTCTCTGGGAG<br>CCCTCAGGAATGAACTTGC<br>TGAAGTGGCCAAGGTGGTA<br>GAACATGGGCTGTTTGTAAA<br>ACGTGTGCTTGGTGATGTC<br>TACAATGATGGGTTCCAGTC	320

				GGTCCAGCCGACCACATGGTACAGGAGGTTCTAGGGTAAGGACAAAACAGG	
GG Bottom 9R	Human ORFeome Gateway entry vector pENTR223-*GBAP1*, complete sequence	GTAGACATCACCAAGCACACGTTTTACAAACAGCCCATGTTCTACCACCTTGGCCACTTCAGCAAGTTCATTCCTGAGGGCTCCCAGAGAGTGGGGCTGGTTGCCAGTCAGAAGAACGACCCGGACGCAGTGGCACTGATGCATCCCGATGGCTCTCCTGTTGTGGTCGTCCTAAACCGCTCCTCTAAGGATGTGCCTCTTACCATCAAGGATCCTGCTGTGGGCTTCCTGGAGACAATCTCACCTGGCTACTCCATTCACACCTACCTGTGGCGT	276	NGNNNNNNNNCNGCTGGACGACTGGACCCATCATTGTAGACATCACCAAGCACACGTTTTACAAACAGCCCATGTTCTACCACCTTGGCCACTTCAGCAAGTTCATTCCTGAGGGCTCCCAGAGAGTGGGGCTGGTTGCCAGTCAGAAGAACGACCCGGACGCAGTGGCACTGATGCATCCCGATGGCTCTCCTGTTGTGGTCGTCCTAAACCGCTCCTCTAAGGATGTGCCTCTTACCATCAAGGATCCTGCTGTGGGCTTCCTGGAGACAATCTCACCTGGCTACTCCATTCACACCTACCTGTGGCGTCGCCA	316

TT Bottom 9F	Human ORFeome Gateway entry vector pENTR223-*GBAP1*, complete sequence	GACCACAACAGGAGAGCCATC<br>GGGATGCATCAGTGCCACTGC<br>GTCCGGGTCGTTCTTCTGACTG<br>GCAACCAGCCCCACTCTCTGG<br>GAGCCCTCAGGAATGAACTTGC<br>TGAAGTGGCCAAGGTGGTAGA<br>ACATGGGCTGTTTGTAAAACGT<br>GTGCTTGGTGATGTCTACAATG<br>ATGGGTTCCAGTCGGTCCAGC<br>CGACCACATGGTACAGGAGGTT<br>CTAGGGTAAGGACAAAACAGGT<br>ACGTGTGAATGGAGTAGCCAG<br>GTGAGATTGTCTCCGGAAGCCA<br>CAGCAGGATCCTTGATGGTAAG<br>AGGCCATCTTAGAGGAGCGGAT<br>TTAGGAGACACAACATG	341	NNNNNNNNNNNNGANNTTG<br>TCTCCNGGAAGCCCACAGC<br>AGGATCCTTGATGGTAAGAG<br>GCACATCCTTAGAGGAGCG<br>GTTTNGGACGACCACAACA<br>GGAGAGCCATCGGGATGCA<br>TCAGTGCCACTGCGTCCGG<br>GTCGTTCTTCTGACTGGCA<br>ACCAGCCCCACTCTCTGGG<br>AGCCCTCAGGAATGAACTT<br>GCTGAAGTGGCCAAGGTGG<br>TAGAACATGGGCTGTTTGTA<br>AAACGTGTGCTTGGTGATG<br>TCTACAATGATGGGTTCCAG<br>TCGGTCCAGCCGACCACAT<br>GGTACAGGAGGTTCTAGGG<br>TAAGGACAAAACAGGTACG<br>TGTGAATGGAGTAGCCAGG<br>TGAGATTGTCTCCGGAAGC<br>CACAGCAGGATCCTTGATG<br>GTAAGAGGCCATCTTAGAG<br>GAGCGGATTTAGGAGACAC<br>AACATGAGAGCCATCAGGAT<br>GCATCCGGCCCTGGGTCCG<br>GTGTTCTTCTCATGCCAACA<br>TCCCCATCTCTGGGTGACC<br>TCTGGAATGAACTTGCTGTA<br>AGTGTGCCACGTGAGTANA<br>ACACGNGCCTGGTTAGGAC<br>AAAGGNGCTTGGNTGAATG<br>TACTAACGACCTCGAGTCTC<br>NN	598

TT Bottom 9R	Human ORFeome Gateway entry vector pENTR223-*GBAP1*, complete sequence	ATCATTGTAGACATCACCAAGCA<br>CACGTTTTACAAACAGCCCATG<br>TTCTACCACCTTGGCCACTTCA<br>GCAAGTTCATTCCTGAGGGCTC<br>CCAGAGAGTGGGGCTGGTTGC<br>CAGTCAGAAGAACGACCCGGA<br>CGCAGTGGCACTGATGCATCCC<br>GATGGCTCTCCTGTTGTGGTCG<br>TCCTAAACCGCTCCTCTAAGGA<br>TGTGCCTCTTACCATCAAGGAT<br>CCTGCTGTGGGCTTCCTGGAG<br>ACAATCTCACCTGGCTACTCCA<br>TTCACACCTACCTGTGGCGTCG<br>CC	286	NNNNNNNGGTNNCTGGACG<br>ACTGGACCCATCATTGTAGA<br>CATCACCAAGC<br>ACACGTTTTACAAACAGCCC<br>ATGTTCTACCACCTTGGCCA<br>CTTCAGCAAG<br>TTCATTCCTGAGGGCTCCC<br>AGAGAGTGGGGCTGGTTGC<br>CAGTCAGAAGAA<br>CGACCCGGACGCAGTGGC<br>ACTGATGCATCCCGATGGCT<br>CTCCTGTTGTGG<br>TCGTCCTAAACCGCTCCTCT<br>AAGGATGTGCCTCTTACCAT<br>CAAGGATCCT<br>GCTGTGGGCTTCCTGGAGA<br>CAATCTCACCTGGCTACTCC<br>ATTCACACCTA<br>CCTGTGGCGTCGCCN	315
G>T Top 9F	Homo sapiens glucosylceramidase beta 1 (*GBA1*), transcript variant 3, mRNA	CAGGAGAGCCATCGGGATGCAT<br>CAGTGCCACTGCGTCCGGGTC<br>GTTCTTCTGACTGGCAACCAGC<br>CCCACTCTCTGGGAGCCCTCA<br>CGAATGAACTTGCTGAAGTGGC<br>CAAGGTGGTAGAACACGGGCT<br>GTTTGTAAAACCCGCCCATGGT<br>GATGTCTACAATGATGGGACCG<br>CCGAAAAAACTCCCCCCCCATT<br>TGGCTCCTCCTTTGCCT	212	NNNNNNNNNNNNNNNNANN<br>GTCTCCNGGAAGCCCACAG<br>CAGGATCCTTGNNGGTAAG<br>AGGCACATCCTTAGAGGAG<br>CGGTTANGNACGACCACAA<br>CAGGAGAGCCATCGGGATG<br>CATCAGTGCCACTGCGTCC<br>GGGTCGTTCTTCTGACTGG<br>CAACCAGCCCCACTCTCTG<br>GGAGCCCTCACGAATGAAC<br>TTGCTGAAGTGGCCAAGGT<br>GGTAGAACACGGGCTGTTT<br>GTAAAACCCGCCCATGGTG<br>ATGTCTACAATGATGGGACC<br>GCCGAAAAAACTCCCCCCC	365

				CATTTGGCTCCTCCTTTGCC<br>TNTAATGGCANAACANTNNT<br>CGGCCCANACGACCCCCTG<br>GTAATTGATGTTCTACGGTA	
G>T Top 9R	Homo sapiens glucosylceramidase beta 1 (*GBA1*), transcript variant 3, mRNA	GCCCTGACCCCTCAAGGAGGA<br>CCCAATTGGGTGCGTAACTTTG<br>TCCACCGTCCCAGNCCTGTTCA<br>CATCACCAATGACACGTTTTACC<br>AACACTCCATGTTCTACCTGCTT<br>GGCCAATTAACCAAGTTCATTC<br>CTGAGGGCTCCCACATAGTGAG<br>GCTGGTTGTGATTCCGAAGACC<br>TAACCGGATCCTCTGGCGCTGA<br>TGCTTCTCCATGGCTCTGCTGT<br>TGTGGTCGTGCTAAAGAGATCC<br>TCTCAGGATGCGCCTCTTACCA<br>TCAACGATCGTGGTGTCGCCAT<br>CCTGGAGACAATCTCACCTGGC<br>TACTCCATTCAACCTACCTGTG<br>GCGTCGCCA	340	GNTTNNGGNNNNTGGANNA<br>CTGGAACCTTGCCCTGACC<br>CCTCAAGGAGGA<br>CCCAATTGGGTGCGTAACTT<br>TGTCCACCGTCCCAGNCCT<br>GTTCACATCAC<br>CAATGACACGTTTTACCAAC<br>ACTCCATGTTCTACCTGCTT<br>GGCCAATTAA<br>CCAAGTTCATTCCTGAGGG<br>CTCCCACATAGTGAGGCTG<br>GTTGTGATTCCG<br>AAGACCTAACCGGATCCTCT<br>GGCGCTGATGCTTCTCCAT<br>GGCTCTGCTGT<br>TGTGGTCGTGCTAAAGAGA<br>TCCTCTCAGGATGCGCCTC<br>TTACCATCAACG<br>ATCGTGGTGTCGCCATCCT<br>GGAGACAATCTCACCTGGC<br>TACTCCATTCAA<br>CCTACCTGTGGCGTCGCCA<br>N	370

G>T Bottom 9F	Human ORFeome Gateway entry vector pENTR223-*GBAP1*, complete sequence	GATTGTCTCCNGGAAGCCCACA<br>GCAGGATCCTTGATGGTAAGAG<br>GCACATCCTTAGAGGAGCGGTT<br>TAGGACGACCACAACAGGAGA<br>GCCATCGGGATGCATCAGTGCC<br>ACTGCGTCCGGGTCGTTCTTCT<br>GACTGGCAACCAGCCCCACTC<br>TCTGGGAGCCCTCAGGAATGAA<br>CTTGCTGAAGTGGCCAAGGTG<br>GTAGAACATGGGCTGTTTGTAA<br>AACGTGTGCTTGGTGATGTCTA<br>CAATGATGGGTTCCAGTCGGTC<br>CAGCCGACCACATGGTACAGG<br>AGGTTCTAGGGTAAGGACAAAA<br>ACAGGANGGTGTGAATGGAGT<br>CTCCAGGTGAGATTGTCTCCGG<br>NAAGCCANAGAAAGATCCTTGA<br>TGGTAANAGGCAATCCTTANNG<br>CGCGGTTTATGACGACCAACAT<br>GGAGA	418	NNNNNNNNNNTNAGATTGT<br>CTCCNGGAAGCCCACAGCA<br>GGATCCTTGATG<br>GTAAGAGGCACATCCTTAGA<br>GGAGCGGTTTAGGACGACC<br>ACAACAGGAGA<br>GCCATCGGGATGCATCAGT<br>GCCACTGCGTCCGGGTCGT<br>TCTTCTGACTGG<br>CAACCAGCCCCACTCTCTG<br>GGAGCCCTCAGGAATGAAC<br>TTGCTGAAGTGG<br>CCAAGGTGGTAGAACATGG<br>GCTGTTTGTAAAACGTGTGC<br>TTGGTGATGTC<br>TACAATGATGGGTTCCAGTC<br>GGTCCAGCCGACCACATGG<br>TACAGGAGGTT<br>CTAGGGTAAGGACAAAAAC<br>AGGANGGTGTGAATGGAGT<br>CTCCAGGTGAGA<br>TTGTCTCCGGNAAGCCANA<br>GAAAGATCCTTGATGGTAAN<br>AGGCAATCCTT<br>ANNGCGCGGTTTATGACGA<br>CCAACATGGAGAGCCATTG<br>GGGA	442

G>T Bottom 9R	Human ORFeome Gateway entry vector pENTR223-*GBAP1*, complete sequence	TCATTGTAGACATCACCAAGCA<br>CACGTTTTACAAACAGCCCATG<br>TTCTACCACCTTGGCCACTTCA<br>GCAAGTTCATTCCTGAGGGCTC<br>CCAGAGAGTGGGGCTGGTTGC<br>CAGTCAGAAGAACGACCCGGA<br>CGCAGTGGCACTGATGCATCCC<br>GATGGCTCTCCTGTTGTGGTCG<br>TCCTAAACCGCTCCTCTAAGGA<br>TGTGCCTCTTACCATCAAGGAT<br>CCTGCTGTGGGCTTCCTGGAG<br>ACAATCTCACCTGGCTACTCCA<br>TTCACACCTACCTGTGGCGTC	282	NNNNNNNNNNNNCTGGACN<br>ACTGGANCCATCATTGTAGA<br>CATCACCAAGC<br>ACACGTTTTACAAACAGCCC<br>ATGTTCTACCACCTTGGCCA<br>CTTCAGCAAG<br>TTCATTCCTGAGGGCTCCC<br>AGAGAGTGGGGCTGGTTGC<br>CAGTCAGAAGAA<br>CGACCCGGACGCAGTGGC<br>ACTGATGCATCCCGATGGCT<br>CTCCTGTTGTGG<br>TCGTCCTAAACCGCTCCTCT<br>AAGGATGTGCCTCTTACCAT<br>CAAGGATCCT<br>GCTGTGGGCTTCCTGGAGA<br>CAATCTCACCTGGCTACTCC<br>ATTCACACCTA<br>CCTGTGGCGTCGCNNN	316
T>G Top 9F	Human ORFeome Gateway entry vector pENTR223-*GBAP1*, complete sequence	CATCGGGATGCATCAGTGCCAC<br>TGCGTCCGGGTCGTTCTTCTGA<br>CTGGCAACCAGCCCCACTCTCT<br>GGGAGCCCTCAGGAATGAACTT<br>GCTGAAGTGGCCAAGGTGGTA<br>GAACATGGGCTGTTTGTAAAAC<br>GTGTGCTTGGTGATGTCTACAA<br>TGATGGGTTCCAGTCGGTCCAG<br>CCGACCACATGGTACAGGAGG<br>TTCTAGGGTAA	207	NNNNNNNNCNNNNNGANNT<br>TGTCTCCNNGNNNCCCACA<br>GCAGGATCCTTGATGGTAA<br>GAGGCACATCCTTAGAGGA<br>GCGGTTNNANANGACCACA<br>ACAGGAGAGCCATCGGGAT<br>GCATCAGTGCCACTGCGTC<br>CGGGTCGTTCTTCTGACTG<br>GCAACCAGCCCCACTCTCT<br>GGGAGCCCTCAGGAATGAA<br>CTTGCTGAAGTGGCCAAGG<br>TGGTAGAACATGGGCTGTTT<br>GTAAAACGTGTGCTTGGTG<br>ATGTCTACAATGATGGGTTC<br>CAGTCGGTCCAGCCGACCA	320

				CATGGTACAGGAGGTTCTA GGGTAAGGACAAAN	
T>G Top 9R	Human ORFeome Gateway entry vector pENTR223-*GBAP1*, complete sequence	GGCCACTTCAGCAAGTTCATTC CTGAGGGCTCCCAGAGAGTGG GGCTGGTTGCCAGTCAGAAGA ACGACCCGGACGCAGTGGCAC TGATGCATCCCGATGGCTCTCC TGTTGTGGTCGTCCTAAACCGC TCCTCTAAGGATGTGCCTCTTA CCATCAAGGATCCTGCTGTGGG CTTCCTGGAGACAATCTCACCT GGCTACTCCATTCACACCTACC TGT	220	GGNNNNGNNNNNNTGGAC CGACTGGACCCATCATTGTA GACATCACCAAGCACACGT TTTACAAACAGCCCATGTTC TACNNCCTTGGCCACTTCA GCAAGTTCATTCCTGAGGG CTCCCAGAGAGTGGGGCTG GTTGCCAGTCAGAAGAACG ACCCGGACGCAGTGGCACT GATGCATCCCGATGGCTCT CCTGTTGTGGTCGTCCTAAA CCGCTCCTCTAAGGATGTG CCTCTTACCATCAAGGATCC TGCTGTGGGCTTCCTGGAG ACAATCTCACCTGGCTACTC CATTCACACCTACCTGTGGC GTCGC	314

T>G Bottom 9F	Human ORFeome Gateway entry vector pENTR223-*GBAP1*, complete sequence	ACCACAACAGGAGAGCCATCGGGATGCATCAGTGCCACTGCGTCCGGGTCGTTCTTCTGACTGGCAACCAGCCCCACTCTCTGGGAGCCCTCAGGAATGAACTTGCTGAAGTGGCCAAGGTGGTAGAACATGGGCTGTTTGTAAAACGTGTGCTTGGTGATGTCTACAATGATGGGTTCCAGTCGGTCCAGCCGACCACATGGTACAGGAGGTTCTAGGGTAAG	224	NNNNNNNNNNGNNAGATTGTCTCCNGGAAGCCCACAGCAGGATCCTTGATGGTAAGAGGCACATCCTTAGAGGAGCGGTTTAGGACGACCACAACAGGAGAGCCATCGGGATGCATCAGTGCCACTGCGTCCGGGTCGTTCTTCTGACTGGCAACCAGCCCCACTCTCTGGGAGCCCTCAGGAATGAACTTGCTGAAGTGGCCAAGGTGGTAGAACATGGGCTGTTTGTAAAACGTGTGCTTGGTGATGTCTACAATGATGGGTTCCAGTCGGTCCAGCCGACCACATGGTACAGGAGGTTCTAGGGTAAGGACAAAACAGGTACGTGTGAATGGAGTAGCCAGGTGAGATTGTCTCCGGNAANCCACAAAGGATCCTTGATGGTAAGAGGCCATCNTTAGAGGAGCGGTTTAGGAGACAAACAGGAAAGCCTTCGGGATGCCTCAGTGCCCTGTGCCCGGTCGTTCTTCTGACTTCCCACCACCCACTCTCTGGGAGCCCTCCCGGATGAACTTGCTNAAAGTGTCCCGGCTGGTATAACATCGGTCGCTGTTGTAAACACCANNTNCTNGTGTTGATGCGTNANNTGAATCGCGATTCNGNNACCTCCAAANTGAANTGNCGCAANANCCNCGNNNNATAAGNCAAAAAC	688

				ACACTTCNCCCGCATNNCG CACCATGGCNCTGAGAN	
T>G Bottom 9R	Human ORFeome Gateway entry vector pENTR223-*GBAP1*, complete sequence	GTAGACATCACCAAGCACACGT TTTACAAACAGCCCATGTTCTAC CACCTTGGCCACTTCAGCAAGT TCATTCCTGAGGGCTCCCAGAG AGTGGGGCTGGTTGCCAGTCA GAAGAACGACCCGGACGCAGT GGCACTGATGCATCCCGATGGC TCTCCTGTTGTGGTCGTCCTAA ACCGCTCCTCTAAGGATGTGCC TCTTACCATCAAGGATCCTGCT GTGGGCTTCCTGGAGACAATCT CACCTGGCTACTCCATTCACAC CTACCTGTGGCGT	276	GNNNNNNGNNCGGNTGGA NCGACTGGACCCATCATTG TAGACATCACCAAGCACAC GTTTTACAAACAGCCCATGT TCTACCACCTTGGCCACTTC AGCAAGTTCATTCCTGAGG GCTCCCAGAGAGTGGGGCT GGTTGCCAGTCAGAAGAAC GACCCGGACGCAGTGGCA CTGATGCATCCCGATGGCT CTCCTGTTGTGGTCGTCCTA AACCGCTCCTCTAAGGATGT GCCTCTTACCATCAAGGATC CTGCTGTGGGCTTCCTGGA GACAATCTCACCTGGCTACT CCATTCACACCTACCTGTGG CGTCGCCAN	318

Supplementary Table 9: Sanger sequencing of excised PCR bands. Sanger sequencing results were visually inspected and select regions were searched in the publically available BLAST database. The best matched BLAST result is listed along with both complete and selected sequences and a total base pair count for each sequence that is provided.

SampleID	Sample Type	Sample Origin	Requested Genotype	Final qPCR Genotype	Note	RIN	Assay Performed
ND01137_GG_OG	LCL	Coriell	GG	GG		9.5	Illumina-RNA, ONT-DNA, ONT-RNA
ND01137_GG_MOCK	LCL	Coriell	GG	GG		9.8	ONT-DNA, ONT-RNA
ND01137_GT	LCL	Coriell	GT	GT		9.8	ONT-DNA, ONT-RNA
ND01137_TT	LCL	Coriell	TT	GT	failed complete editing	9.8	ONT-DNA, ONT-RNA
ND22789_TT_OG	LCL	Coriell	TT	TT		10.0	Illumina-RNA, ONT-DNA, ONT-RNA
ND22789_TT_MOCK	LCL	Coriell	TT	TT		9.9	ONT-DNA, ONT-RNA
ND22789_GT	LCL	Coriell	GT	GT		10.0	ONT-DNA, ONT-RNA
ND22789_GG	LCL	Coriell	GG	GG		9.9	ONT-DNA, ONT-RNA

Supplementary Table 10: CRISPR edited LCLs for rs3115534. All original LCLs were obtained from Coriell. CRISPR editing was performed by Synthego. ND01137 was originally rs3115534-GG while ND22789 was originally rs3115534-TT. Both lines were mock transfected, partially edited to yield heterozygote pools, and completely edited to homozygotes for the inverse allele. Note that complete editing of ND01137 to rs3115534-TT was not successful and genotyping revealed a heterozygous cell pool. Downstream analyses performed are listed by line.

Dataset	Region	n total	TT count	TG count	GG count	Analysis	Estimate	Std Error	P	Multiple R2	Adjusted R2
1000Genomes LCL	intron8_40bptranscript_only	88	41	40	7	linear regression GG, GT, TT	0.099	0.007	2.47E-24	0.702	0.699
1000Genomes LCL	exon9	88	41	40	7	linear regression GG, GT, TT	0.017	0.041	0.674	0.002	-0.010
1000Genomes LCL	exon8	88	41	40	7	linear regression GG, GT, TT	-0.020	0.035	0.566	0.004	-0.008
1000Genomes LCL	intron8	88	41	40	7	linear regression GG, GT, TT	0.097	0.005	4.08E-31	0.793	0.790
1000Genomes LCL	intron8_minus_40transcript	88	41	40	7	linear regression GG, GT, TT	0.097	0.005	8.36E-31	0.789	0.787
1000Genomes LCL	*GBA1*	88	41	40	7	linear regression GG, GT, TT	0.001	0.005	0.794	0.001	-0.011
1000Genomes LCL	*GBA1LP*	88	41	40	7	linear regression GG, GT, TT	-0.002	0.003	0.541	0.004	-0.007
AMP-PD Blood	intron8_40bptranscript_only	146	98	47	1	linear regression GG + GT , TT	0.026	0.005	3.65E-07	0.165	0.159
AMP-PD Blood	exon9	146	98	47	1	linear regression GG + GT , TT	0.002	0.010	0.865	0.000	-0.007
AMP-PD Blood	exon8	146	98	47	1	linear regression GG + GT , TT	0.000	0.010	0.98	0.000	-0.007
AMP-PD Blood	intron8	146	98	47	1	linear regression GG + GT , TT	0.009	0.002	8.05E-07	0.156	0.150
AMP-PD Blood	intron8_minus_40transcript	146	98	47	1	linear regression GG + GT , TT	0.007	0.002	2.18E-05	0.118	0.112

AMP-PD Blood	*GBA1*	146	98	47	1	linear regression GG + GT , TT	2.140	1.280	0.097	0.019	0.012
AMP-PD Blood	*GBA1LP*	146	98	47	1	linear regression GG + GT , TT	0.322	1.008	0.75	0.001	-0.006
HBCC Frontal Cortex ONT	intron8_40bptranscript_only	8	2	2	4	linear regression GG + GT , TT	0.155	0.129	0.274	0.195	0.060
HBCC Frontal Cortex ONT	exon9	8	2	2	4	linear regression GG + GT , TT	-2.718	1.443	0.109	0.372	0.267
HBCC Frontal Cortex ONT	exon8	8	2	2	4	linear regression GG + GT , TT	-2.397	1.218	0.097	0.392	0.291
HBCC Frontal Cortex ONT	intron8	8	2	2	4	linear regression GG + GT , TT	0.009	0.007	0.281	0.189	0.054
HBCC Frontal Cortex ONT	intron8_minus_40transcript	8	2	2	4	linear regression GG + GT , TT	0.008	0.007	0.293	0.182	0.045
HBCC Frontal Cortex ONT	*GBA1*	8	2	2	4	linear regression GG + GT , TT	-0.543	0.263	0.085	0.415	0.317
HBCC Frontal Cortex ONT	*GBA1LP*	8	2	2	4	linear regression GG + GT , TT	0.032	0.120	0.795	0.012	-0.153
HBCC Frontal Cortex Illumina	intron8_40bptranscript_only	92	66	20	6	linear regression GG, GT, TT	0.019	0.002	3.57E-12	0.417	0.411
HBCC Frontal Cortex Illumina	exon9	92	66	20	6	linear regression GG, GT, TT	-0.107	0.036	8.00E-03	0.076	0.065
HBCC Frontal Cortex Illumina	exon8	92	66	20	6	linear regression GG, GT, TT	-0.113	0.033	1.00E-03	0.113	0.103
HBCC Frontal Cortex Illumina	intron8	92	66	20	6	linear regression GG, GT, TT	0.016	0.002	2.76E-15	0.502	0.497

HBCC Frontal Cortex Illumina	intron8_minus_40transcript	92	66	20	6	linear regression GG, GT, TT	0.016	0.002	5.45E-15	0.495	0.489
HBCC Frontal Cortex Illumina	*GBA1*	92	66	20	6	linear regression GG, GT, TT	-0.015	0.005	6.00E-03	0.082	0.072
HBCC Frontal Cortex Illumina	*GBA1LP*	92	66	20	6	linear regression GG, GT, TT	0.007	0.002	3.00E-03	0.092	0.082
Coriell Illumina LCL	intron8_40bptranscript_only	18	8	9	1	linear regression GG + GT , TT	0.081	0.018	3.12E-04	0.567	0.540
Coriell Illumina LCL	exon9	18	8	9	1	linear regression GG + GT , TT	-0.168	0.095	0.0957	0.164	0.112
Coriell Illumina LCL	exon8	18	8	9	1	linear regression GG + GT , TT	-0.024	0.099	0.813	0.004	-0.059
Coriell Illumina LCL	intron8	18	8	9	1	linear regression GG + GT , TT	0.056	0.011	3.88E-05	0.663	0.642
Coriell Illumina LCL	intron8_minus_40transcript	18	8	9	1	linear regression GG + GT , TT	0.057	0.011	6.65E-05	0.641	0.618
Coriell Illumina LCL	*GBA1*	18	8	9	1	linear regression GG + GT , TT	-0.001	0.014	0.934	0.000	-0.062
Coriell Illumina LCL	*GBA1LP*	18	8	9	1	linear regression GG + GT , TT	0.005	0.006	0.358	0.053	-0.006
Coriell ONT LCL	intron8_40bptranscript_only	8	3	4	1	linear regression GG + GT , TT	0.331	0.072	3.74E-03	0.778	0.741
Coriell ONT LCL	exon9	8	3	4	1	linear regression GG + GT , TT	0.572	0.470	0.269	0.198	0.064
Coriell ONT LCL	exon8	8	3	4	1	linear regression GG + GT , TT	0.093	0.362	0.805	0.011	-0.154

Coriell ONT LCL	intron8	8	3	4	1	linear regression GG + GT , TT	0.156	0.052	0.0239	0.601	0.534
Coriell ONT LCL	intron8_minus_40transcript	8	3	4	1	linear regression GG + GT , TT	0.136	0.054	0.045	0.515	0.434
Coriell ONT LCL	*GBA1*	8	3	4	1	linear regression GG + GT , TT	0.049	0.081	0.563	0.059	-0.098
Coriell ONT LCL	*GBA1LP*	8	3	4	1	linear regression GG + GT , TT	0.007	0.018	0.726	0.022	-0.141
CRISPR Coriell ONT LCL	intron8_40bptranscript_only	7	2	2	3	linear regression GG + GT , TT	0.264	0.093	0.036	0.617	0.540
CRISPR Coriell ONT LCL	exon9	7	2	2	3	linear regression GG + GT , TT	-1.166	0.328	0.016	0.717	0.660
CRISPR Coriell ONT LCL	exon8	7	2	2	3	linear regression GG + GT , TT	-1.313	0.197	0.001	0.899	0.879
CRISPR Coriell ONT LCL	intron8	7	2	2	3	linear regression GG + GT , TT	0.143	0.038	0.014	0.734	0.681
CRISPR Coriell ONT LCL	intron8_minus_40transcript	7	2	2	3	linear regression GG + GT , TT	0.129	0.045	0.035	0.621	0.545
CRISPR Coriell ONT LCL	*GBA1*	7	2	2	3	linear regression GG + GT , TT	-0.250	0.055	0.006	0.803	0.764
CRISPR Coriell ONT LCL	*GBA1LP*	7	2	2	3	linear regression GG + GT , TT	-0.003	0.019	0.869	0.006	-0.193

**Dataset**	**Region**	**n total**	**AA count**	**AT count**	**TT count**	**Analysis**	**Estimate**	**Std Error**	**P**	**Multiple R2**	**Adjusted R2**
AMP-PD Blood	exon7	143	101	42	0	linear regression AA, AT	0.002	0.012	0.888	0.000	-0.007

AMP-PD Blood	exon6	143	101	42	0	linear regression AA, AT	0.010	0.015	0.509	0.003	-0.004
AMP-PD Blood	intron6	143	101	42	0	linear regression AA, AT	0.004	0.003	0.264	0.009	0.002
AMP-PD Blood	*GBA1*	143	101	42	0	linear regression AA, AT	0.002	0.003	0.463	0.004	-0.003

Dataset	n total	TT count	TG count	GG count	Analysis	Estimate	Std Error	P	Multiple R2	Adjusted R2
UKB Protein	1147	753	351	43	linear regression GG, GT , TT	-0.074	0.027	5.92E-03	0.007	0.006

Dataset	n total	TT count	TG count	GG count	Analysis	Estimate	Std Error	P	Multiple R2	Adjusted R2
GCase Activity	205	97	99	9	linear regression GG, GT , TT	-0.449	0.205	0.029	0.023	0.018

Supplementary Table 11: Overview of statistical tests performed for coverage. A variety of linear regressions were performed to compare coverage for various sequence regions from multiple datasets including, 1000Genomes LCL, AMP-PD Blood, HBCC Frontal Cortex ONT, HBCC Frontal Cortex Illumina, Coriell Illumina LCL, Coriell ONT LCL, CRISPR Coriell ONT LCL, AMP-PD Blood, and Centogene GCase.

		MAIN TRANSCRIPT - ENST00000368373			NOVEL TRANSCRIPT			RATIO	PRIMARY ALIGNED READS	
**Coriell LCLs**		**COV**	**FPKM**	**TPM**	**COV**	**FPKM**	**TPM**	**TPM/TPM**	**WHOLE**	***GBA1***
ND01137	GG	48.82	1.37	1.32	21.40	0.60	0.58	0.439	41112496	156
ND02892	GT	72.50	1.99	1.91	1.14	0.03	0.03	0.016	43776753	147
ND00079	GT	139.89	3.10	2.93	4.14	0.09	0.09	0.031	51669202	337
ND05171	GT	83.48	1.66	1.58	NA	NA	NA	NA	58854214	240
ND04724	GT	81.28	2.23	2.01	12.07	0.33	0.30	0.149	46129222	297
ND01078	TT	83.97	1.82	1.58	NA	NA	NA	NA	60267891	280
ND01177	TT	68.18	1.90	1.72	NA	NA	NA	NA	43530770	184
ND25221	TT	69.06	1.88	1.60	NA	NA	NA	NA	49449115	162

* Note: ND05171 showed transcript upon manual inspection on IGV

		MAIN TRANSCRIPT - ENST00000368373			NOVEL TRANSCRIPT			RATIO	PRIMARY ALIGNED READS	
**Coriell CRISPR LCLs**		**COV**	**FPKM**	**TPM**	**COV**	**FPKM**	**TPM**	**TPM/TPM**	**WHOLE**	***GBA1***
ND01137	GG-OG	48.82	1.37	1.32	21.40	0.60	0.58	0.439	41112496	156
ND01137	GG-Mock	67.61	1.94	1.53	8.25	0.24	0.19	0.124	50035605	202
ND01137	GT	23.59	0.87	0.73	NA	NA	NA	NA	35765096	107
ND22789	TT_OG	87.78	2.23	1.94	NA	NA	NA	NA	49035038	214
ND22789	TT-Mock	61.18	2.93	2.74	NA	NA	NA	NA	25748942	162
ND22789	GT	36.24	1.54	1.36	3.91	0.17	0.15	0.110	30152550	108
ND22789	GG	31.1	1.28	1.12	3.79	0.16	0.14	0.125	31527832	111

* Note: ND01137 GT showed transcript upon manual inspection on IGV

		MAIN TRANSCRIPT - ENST00000368373			NOVEL TRANSCRIPT			RATIO	PRIMARY ALIGNED READS	
HBCC Frontal Cortex		COV	FPKM	TPM	COV	FPKM	TPM	TPM/TPM	WHOLE	*GBA1*
HBCC_82014	GG	90.52	6.23	7.57	4.43	0.30	0.37	0.049	21272803	236
HBCC_81996	GG	9.05	3.38	4.08	NA	NA	NA	NA	3752987	33
HBCC_82040	GG	25.19	3.52	4.34	NA	NA	NA	NA	11807350	59
HBCC_82061	GG	5.85	1.42	1.67	NA	NA	NA	NA	7610114	14
HBCC_82004	GT	57.04	5.95	7.35	NA	NA	NA	NA	13698898	122
HBCC_82006	GT	120.73	7.11	8.20	NA	NA	NA	NA	23686272	213
HBCC_81985	TT	152.28	9.39	10.31	NA	NA	NA	NA	21401748	293
HBCC_81994	TT	60.47	6.37	7.07	NA	NA	NA	NA	14217204	134

Supplementary Table 12: Transcript quantifications using Stringtie2. Normalized transcript counts for original LCLs, CRISPR edited LCLs, and HBCC brain samples are shown. COV-coverage; FPKM-fragments per kilobase of transcript per million mapped reads; TPM-transcripts per million; RATIO-short transcript TPM divided by main transcript TPM; PRIMARY ALIGNED READS: WHOLE-all primary aligned read counts whole transcriptome; GBA-count of primary reads aligned to *GBA1*.

Please see separate excel file called: Sup_13_Mass_spectrometry_analysis.xlsx

Supplementary Table 13: Mass spectrometry analysis of excised 4-20% agarose gel region predicted to contain truncated *GBA1* protein. rs3115534-GG (replicate 1) and (replicate 2) represent cell lysates from the same stock vial of ND01137 LCLs electrophoresed in parallel on the same gel and excised at the same time. These correspond to lanes B and C of **Supplementary Figure 11**. The remaining samples rs3115534-GT and rs3115534-TT correspond to lanes D and E, respectively. No known *GBA1* motifs were detected. # PSM-peptide-spectrum match number.

Sample	rs3115534 Genotype	Gene	All reads	Reads with 10x barcodes
82040	GG	*GBA1*	5034	409
		GBA1LP	1976	182
82041	TT	*GBA1*	50909	2175
		GBA1LP	31145	2399

Supplementary Table 14: Reads mapping to *GBA1* after 10x capture enrichment. All primary aligned reads mapping to *GBA1* or *GBA1LP* after performing the 10x capture enrichment for *GBA1* are shown. For downstream analysis of cell type specificity, the number of primary aligned reads with 10x barcodes was also quantified.

CHR	POS	ID	ID_RS	REF
chr1	155237596	1:155237596:A:T	rs140335079	A
chr1	155235878	1:155235878:G:T	rs3115534	G
chr1	155240729	1:155240729:A:C	rs745734072	A

Supplementary Table 15: *GBA1* variants affecting branchpoint sequences according to AGAIN algorithm. List of three variants of interest in *GBA1* obtained from gnomAD that are predicted to impact a branchpoint sequence. See **Figure 4** and **Supplementary Figure 22** for a depiction of the scale of branchpoint sequence impact of these variants. CHR-chromosome; POS-base pair; ID-Chr:Pos:Ref:Alt; ID_RS-available rs ID; REF-reference allele.
