# Supplementary material for: African ancestry neurodegeneration risk variant disrupts an intronic branchpoint in GBA1
Source: Nat Struct Mol Biol. 2024 Dec 12;31(12):1955–63. doi: 10.1038/s41594-024-01423-2 (PMC11638064; doi:10.1038/s41594-024-01423-2)
Supplement: Supplementary file 2 — Reporting Summary [file 41594_2024_1423_MOESM2_ESM.pdf]

nature portfolio

Corresponding author(s): Cornelis Blauwendraat

Last updated by author(s): Aug 14, 2024

Reporting Summary

Nature Portfolio wishes to improve the reproducibility of the work that we publish. This form provides structure for consistency and transparency in reporting. For further information on Nature Portfolio policies, see our Editorial Policies and the Editorial Policy Checklist.

Statistics

For all statistical analyses, confirm that the following items are present in the figure legend, table legend, main text, or Methods section.

n/a	Confirmed	
☐	☒	The exact sample size (*n*) for each experimental group/condition, given as a discrete number and unit of measurement
☐	☒	A statement on whether measurements were taken from distinct samples or whether the same sample was measured repeatedly
☐	☒	The statistical test(s) used AND whether they are one- or two-sided<br>*Only common tests should be described solely by name; describe more complex techniques in the Methods section.*
☐	☒	A description of all covariates tested
☐	☒	A description of any assumptions or corrections, such as tests of normality and adjustment for multiple comparisons
☐	☒	A full description of the statistical parameters including central tendency (e.g. means) or other basic estimates (e.g. regression coefficient) AND variation (e.g. standard deviation) or associated estimates of uncertainty (e.g. confidence intervals)
☐	☒	For null hypothesis testing, the test statistic (e.g. *F*, *t*, *r*) with confidence intervals, effect sizes, degrees of freedom and *P* value noted<br>*Give P values as exact values whenever suitable.*
☒	☐	For Bayesian analysis, information on the choice of priors and Markov chain Monte Carlo settings
☒	☐	For hierarchical and complex designs, identification of the appropriate level for tests and full reporting of outcomes
☒	☐	Estimates of effect sizes (e.g. Cohen's *d*, Pearson's *r*), indicating how they were calculated

Our web collection on statistics for biologists contains articles on many of the points above.

Software and code

Policy information about availability of computer code

Data collection	All scripts and code for this project can be found at: https://github.com/GP2code/GBA1-rs3115534-branchpoint<br>Tools used for data collection involve:<br>Minknow 22.10.7<br>RTA v3<br>Illumina NextSeq500<br>QuantStudio 6 Pro
Data analysis	All scripts and code for this project can be found at: https://github.com/GP2code/GBA1-rs3115534-branchpoint<br>Tools used for data analysis involve:<br>Guppy 6.1.2<br>Minimap2 2.24<br>Minimap2 2.26<br>Sniffles 2.2<br>Samtools 1.17<br>Clair3 1.0.4<br>Pychopper 2.7.1<br>Stringtie 2.2.1<br>IGV 2.16.0<br>R 4.3.0<br>ggplot2<br>Virtual Studio Code

bcl2fast1 2.20.0.422
STAR 2.7.10
STAR 2.6.1
BWA 0.5.9
RegulomeDB 2.2
AGAIN
SpliceAI
Branchpointer 4.3.0
Proteome Discoverer 2.4
Illumina DRAGEN 3.7.8
Plink 2.0

For manuscripts utilizing custom algorithms or software that are central to the research but not yet described in published literature, software must be made available to editors and reviewers. We strongly encourage code deposition in a community repository (e.g. GitHub). See the Nature Portfolio guidelines for submitting code & software for further information.

Data

Policy information about availability of data

All manuscripts must include a data availability statement. This statement should provide the following information, where applicable:

- Accession codes, unique identifiers, or web links for publicly available datasets
- A description of any restrictions on data availability
- For clinical datasets or third party data, please ensure that the statement adheres to our policy

Unedited Coriell LCL lines are available at https://www.coriell.org/. CRISPR edited Coriell LCL lines are available upon request and establishment of an MTA with Coriell and NIH/CARD abiding by the Coriell NINDS Human Genetics Repository Material Transfer Agreement For Biospecimens. All generated LCL Coriell ONT DNAseq, CAGEseq and RNAseq data (ILM and ONT) is available at https://www.amp-pd.org/ via GP2 tier 2 access which is obtainable via filling in the form https://www.amp-pd.org/researchers/data-use-agreement. It is part of the following release DOI 10.5281/zenodo.10962119; release 7; https://console.cloud.google.com/storage/browser/gp2tier2/release7_30042024/gp2_omics/Alvarez_Jerez_et_al_2024. Additionally, data path is as follows: gp2tier2/release7_30042024/gp2_omics/Alvarez_Jerez_et_al_2024. AMP-PD ILM blood based RNAseq is available at https://www.amp-pd.org/ after signing the data use agreement. 1000 Genomes project data is publicly available at https://www.internationalgenome.org/. Brain tissue bulk RNAseq is available at https://www.ncbi.nlm.nih.gov/projects/gap/cgi-bin/study.cgi?study_id=phs000979.v3.p2 and frontal cortex data athttps://nda.nih.gov/edit_collection.html?id=3151. Summary statistics for cis-eQTLs and a catalog of ancestry-specific eQTLs from Kachuri et al.12 were obtained from https://doi.org/10.5281/zenodo.7735723.

Research involving human participants, their data, or biological material

Policy information about studies with human participants or human data. See also policy information about sex, gender (identity/presentation), and sexual orientation and race, ethnicity and racism.

Reporting on sex and gender	Sex was used exclusively in this study, and was either self-reported or confirmed through genetic data analysis. No conclusions of this manuscript pertain to only one sex nor where any sex-specific analyses performed.
Reporting on race, ethnicity, or other socially relevant groupings	Ancestries referenced in this study were determined through genetic data analysis and are specified throughout this paper.
Population characteristics	Population characteristics, where available, are specified in the supplementary information for each study cohort. In summary:<br>LCL Coriell samples included 9 females and 9 males with an average age of 60 yo.<br>ONT HBCC Samples included 3 females and 6 males with an average age of 38 yo.<br>Illumina HBCC samples included 33 females and 59 males with an average age of 44 yo.<br>AMP-PD samples included 65 females and 81 males with an average age of 62yo.<br>1000 Genomes samples included 48 females and 40 males. Age is not available for these samples due to consent.<br><br>All samples are of African, African Admixed, or African American ancestry with the exception of 118 AMP-PD samples that are European.
Recruitment	No specific participant recruitment was utilized in this study. We accessed publicly available data or biosamples from entities such as the UK Biobank, 1000Genomes, AMP-PD, Coriell Institute of Medicine, or the Human Brain Collection Core.
Ethics oversight	All our research complies with the relevant ethical regulations. The work is covered by local IRB approval at each site involved.

Note that full information on the approval of the study protocol must also be provided in the manuscript.

Field-specific reporting

Please select the one below that is the best fit for your research. If you are not sure, read the appropriate sections before making your selection.

☒ Life sciences ☐ Behavioural & social sciences ☐ Ecological, evolutionary & environmental sciences

Life sciences study design

All studies must disclose on these points even when the disclosure is negative.

Sample size	Sample sizes were determined by availability of samples with different with different rs3115534 genotypes. Sample sizes were also determined by available genetic data in samples of African or African Admixed ancestry.
Data exclusions	Samples were excluded based on ancestry where available. The only exception was the inclusion of European samples in AMP-PD data to increase the control group. Once samples were picked based on ancestry no other exclusions were applied.
Replication	Conclusions drawn for rs3115534 investigation in available biosamples were replicated in multiple publicly accessible datasets all of which are detailed in manuscript. For original finding of intron 8 retention in the LCL ONT data, that was replicated in LCL Illumina, CRISPR ONT sequencing, AMP-PD samples, frontal cortex ONT sequencing, and 1000Genomes cohort.<br>Single cell sequencing was only performed once.<br>The CRISPR PCR was run twice.<br>Sanger sequencing was only performed once. Wester blot experiments were run three times with both ECL and fluorescent methods.
Randomization	Samples were allocated to experimental groups based on their rs3115534 genotype for all analyses.
Blinding	Blinding was not relevant to our study, as we had to choose samples with specific genotypes.

Reporting for specific materials, systems and methods

We require information from authors about some types of materials, experimental systems and methods used in many studies. Here, indicate whether each material, system or method listed is relevant to your study. If you are not sure if a list item applies to your research, read the appropriate section before selecting a response.

Materials & experimental systems

n/a	Involved in the study	
☐	☒	Antibodies
☐	☒	Eukaryotic cell lines
☒	☐	Palaeontology and archaeology
☒	☐	Animals and other organisms
☒	☐	Clinical data
☒	☐	Dual use research of concern
☒	☐	Plants

Methods

n/a	Involved in the study	
☒	☐	ChIP-seq
☒	☐	Flow cytometry
☒	☐	MRI-based neuroimaging

Antibodies

Antibodies used	Primary glucocerebrosidase (1 ug/mL working concentration, 1:1000 dilution, Sigma-Aldrich, Polyclonal Clone G4171)<br>β-actin (1 ug/mL working concentration, 1:1000 dilution, Abcam, Monoclonal Clone mAbcam 8224)<br>Donkey anti-Rabbit (LiCor Biosciences, Lot No. D30328-05, 926-68073, 1:20,000 dilution) secondary antibody<br>Donkey anti-Mouse (LiCor Biosciences, Lot No. D30124-05, 926-32212, 1:20,000 dilution) secondary antibody<br>Goat anti-Rabbit IgG (H+L) Cross-Adsorbed Secondary Antibody, HRP (1:1000 dilution, Invitrogen, 31462)<br>Goat anti-Mouse IgG (H+L) Cross-Adsorbed Secondary Antibody, HRP (1:1000 dilution, Invitrogen, 31432)
Validation	Per Sigma Aldrich, primary glucocerebrosidase antibody (G4171, Sigma Aldrich) was validated as follows: "Emhanced antibody validation is an assay or method that provides researchers with additional assurance that the antibody specificity for the target antigen agrees with previously defined expression data". Additionally, guide on antibody validation for Sigma Aldrich can be found here: https://www.sigmaaldrich.com/US/en/technical-documents/technical-article/protein-biology/elisa/antibody-standard-validation<br>Per Abcam, primary β-actin (mAbcam 8224, Abcam) was validated and tested by the manufacturer in multiple cell lines with consistent and accurate results. Data from experiments performed by the manufacturer is available here:https://www.abcam.com/en-us/products/primary-antibodies/beta-actin-antibody-mabcam-8224-loading-control-ab8224#application=wb

Eukaryotic cell lines

Policy information about cell lines and Sex and Gender in Research

Cell line source(s)	All cell lines came from the Coriell Institute for Medical Research. Sex for each line is reported in Supplementary Tab;e 1 of the manuscript. In Summary, 9 were male and 9 were female.<br>The 293Flp-In cells are a commercially available: https://www.thermofisher.com/order/catalog/product/R78007.<br>These are derived from human HEK293, which is of female origin: https://www.synthego.com/hek293#:~:text=HEK293%20is

	%20a%20hypotriploid%20human,genome%2C%20which%20displays%20cytogenetic%20instability.
Authentication	Cell line authentication and QC was done by Coriell Institute for Medical Research. Additionally, Coriell cell lines underwent qPCR to confirm the annotated genotype at rs3115534.<br><br>For the 293Flp-In cells, they are periodically tested as describe here Damianov, A. et al. The splicing regulators RBM5 and RBM10 are subunits of the U2 snRNP engaged with intron branch sites on chromatin. Mol. Cell 84, 1496–1511.e7 (2024).
Mycoplasma contamination	All cell line checks were performed by the manufacturers and tested negative for mycoplasma contamination.
Commonly misidentified lines<br>(See ICLAC register)	No commonly misidentified lines were used in the study.

Plants

Seed stocks	NA
Novel plant genotypes	NA
Authentication	NA
