# Supplementary material for: African ancestry neurodegeneration risk variant disrupts an intronic branchpoint in GBA1
Source: Nat Struct Mol Biol. 2024 Dec 12;31(12):1955–63. doi: 10.1038/s41594-024-01423-2 (PMC11638064; doi:10.1038/s41594-024-01423-2)
Supplement: Supplementary file 4 — Full list of the GP2 consortium members. [file 41594_2024_1423_MOESM4_ESM.pdf]

Country	Name	Institution	City	State/Province
**Algeria**	Yasser Mecheri	Centre Hospitalo-Universitaire Dr Benbadis	Constantine	
**Argentina**	Emilia M Gatto	Sanatorio de la Trinidad Mitre- INEBA	Buenos Aires	
	Marcelo Kauffman	Hospital JM Ramos Mejia	Buenos Aires	
	Federico Capparelli	Centro de Educación Médica e Investigaciones Clínic	Buenos Aires	
**Armenia**	Samson Khachatryan	Somnus Neurology Clinic	Yerevan	
	Zaruhi Tavadyan	Somnus Neurology Clinic	Yerevan	
	Mariam Isayan	Somnus Neurology Clinic	Yerevan	
**Australia**	Claire E Shepherd	Neuroscience Research Australia	Sydney	New South Wales
	Kishore Kumar	Garvan Institute of Medical Research and Concord	Darlinghurst	New South Wales
	Melina Ellis	Concord Hospital	Concord	New South Wales
	Miguel E. Rentería	QIMR Berghofer Medical Research Institute	Herston	Queensland
	Sulev Koks	Murdoch University	Perth	Western Australia
	Simon Rowe	Neuroscience Research Australia	Sydney	New South Wales
	Laura Rudaks	Neuroscience Research Australia	Sydney	New South Wales
	Dennis Yeow	Neuroscience Research Australia	Sydney	New South Wales
	Carolyn Sue	Neuroscience Research Australia	Sydney	New South Wales
	Victor Flores Ocampo	QIMR Berghofer Medical Research Institute	Brisbane	Queensland
**Austria**	Alexander Zimprich	Medical University Vienna Austria	Vienna	
**Azerbaijan**	Kanan Jafarov	Istanbul Klinik	Baku	
**Belgium**	David Crosiers	University of Antwerp	Antwerp	
**Brazil**	Artur F. Schumacher-Schuh	Universidade Federal do Rio Grande do Sul /	Porto Alegre	
	Carlos Rieder	Federal University of Health Sciences of Porto	Porto Alegre	
	Paula Saffie Awad	Universidade Federal do Rio Grande do Sul	Porto Alegre	
	Vitor Tumas	University of São Paulo	São Paulo	
	Sarah Camargos	Universidade Federal de Minas Gerais	Belo Horizonte	
	Lucas Faria Costa	Universidade Federal de Minas Gerais	Belo Horizonte	
**Canada**	Edward A. Fon	Montreal Neurological Institute	Montreal	Quebec
	Oury Monchi	Institut universitaire de gériatrie de Montréal	Montreal	Quebec
	Ted Fon	McGill University	Montreal	Quebec
	Robert Thibault	Aligning Science Across Parkinson's	Vancouver	British Columbia
	Ziv Gan-Or	McGill University	Montreal	Quebec
	Anthony Lang	University of Toronto	Toronto	Ontario
	Konstantin Senkevich	McGill University	Montreal	Quebec
**Chile**	Benjamin Pizarro Galleguillos	Universidad de Chile	Santiago	
	Marcelo Miranda	Fundación Diagnosis	Santiago	
	Maria Leonor Bustamante	Faculty of Medicine Universidad de Chile	Santiago	
	Patricio Olguin	Universidad de Chile	Santiago	
	Pedro Chana	CETRAM	Santiago	
	Elias Fernandez	Hospital Regional de Concepción	Concepcion	
	Beisha Tang	Central South University	Changsha	
	Huifang Shang	West China Hospital Sichuan University	Chengdu	

Country	Name	Institution	City	State/Province
China	Jifeng Guo	Xiangya Hospital	Changsha	
	Piu Chan	Capital Medical University	Beijing	
	Wei Luo	Zhejiang University	Hangzhou	
	Zhenhua Liu	Xiangya Hospital, Central South University	Changsha	
Colombia	Gonzalo Arboleda	Universidad Nacional de Colombia	Bogotá	
	Jorge Orozco	Fundación Valle del Lili	Santiago De Cali	
	Marlene Jimenez del Rio	University of Antioquia	Medellin	
Costa Rica	Alvaro Hernandez	University of Costa Rica	San Jose	
Denmark	Per Borghammer	Aarhus University	Aarhus	
Egypt	Mohamed Salama	The American University in Cairo	Cairo	
	Walaa A. Kamel	Beni-Suef University	Beni Suef	
Ethiopia	Yared Z. Zewde	Addis Ababa University	Addis Ababa	
France	Alexis Brice	Paris Brain Institute	Paris	
	Jean-Christophe Corvol	Sorbonne Université	Paris	
	Mari Vidailhet	Salpêtriere Hospital (AP-HP), Sorbonne Université	Paris	
Georgia	Mariam Kekenadze	Tbilisi State Medical University	Tbilisi	
	Irine Khatiashvili	S. Khechinashvili University Hospital	Tbilisi	
Germany	Ana Westenberger	University of Lübeck	Lübeck	
	Anastasia Illarionova	Deutsches Zentrum für Neurodegenerative	Göttingen	
	Brit Mollenhauer	University Medical Center Göttingen	Göttingen	
	Christine Klein	University of Lübeck	Lübeck	
	Eva-Juliane Vollstedt	University of Lübeck	Lübeck	
	Franziska Hopfner	Department of Neurology, University Hospital, LMU	Munich	
	Günter Höglinger	Department of Neurology, University Hospital, LMU	Munich	
	Harutyun Madoev	University of Lübeck	Lübeck	
	Joanne Trinh	University of Lübeck	Lübeck	
	Johanna Junker	University of Lübeck	Lübeck	
	Katja Lohmann	University of Lübeck	Lübeck	
	Lara M. Lange	University of Lübeck and University Medical Center	Lübeck	
	Manu Sharma	University of Tubingen	Tübingen	
	Sergiu Groppa	University of Mainz	Mainz	
	Thomas Gasser	University of Tübingen	Tübingen	
	Zih-Hua Fang	The German Center for Neurodegenerative	Göttingen	
	Karl Heilbron	Charité - Universitätsmedizin Berlin	Berlin	
	Theresa Luth	University of Luebeck	Lübeck	
	Wenhua Sun	University of Teubingen	Tübingen	
	Inke König	University of Lübeck	Lübeck	
	Daniela Berg	University Medical Center Schleswig-Holstein	Lübeck	
	Bernhard Haslinger	Technical University of Munich	Munich	
Ghana	Albert Akpalu	University of Ghana Medical School	Accra	

Country	Name	Institution	City	State/Province
Greece	Georgia Xiromerisiou	University of Thessaly	Volos	
	Georgios Hadjigorgiou	University of Thessaly	Volos	
	Ioannis Dagklis	Aristotle University of Thessaloniki	Thessaloniki	
	Ioannis Tarnanas	Ionian University	Corfu	
	Leonidas Stefanis	Biomedical research Foundation of the Academy of	Athens	
	Maria Stamelou	Diagnostic and Therapeutic Centre HYGEIA	Marousi	
	Efthymios Dadiotis	University of Thessaly	Volos	
Honduras	Alex Medina	Hospital San Felipe	Tegucigalpa	
Hong Kong	Germaine Hiu-Fai Chan	Queen Elizabeth Hospital	Kowloon	
	Nancy Ip	The Hong Kong University of Science and	Kowloon	
	Nelson Yuk-Fai Cheung	Queen Elizabeth Hospital	Kowloon	
	Phillip Chan	The Hong Kong University of Science and	Kowloon	
	Xiaopu Zhou	The Hong Kong University of Science and	Kowloon	
India	Asha Kishore	Aster Medcity	Kochi	
	Divya KP	Sree Chitra Tirunal Institute for Medical Sciences	Thiruvananthapuram	
	Pramod Pal	National Institute of Mental Health & Neurosciences	Bengaluru	
	Prashanth Lingappa Kukkle	Manipal Hospital	Delhi	
	Roopa Rajan	All India Institute of Medical Sciences	Delhi	
	Rupam Borgohain	Nizam's Institute Of Medical Sciences	Hyderabad	
Iran	Mehri Salari	Shahid Beheshti University of Medical Science	Tehran	
Israel	Tamara Shiner	Tel Aviv Sourasky Medical Center	Tel Aviv-Yafo	
	Avner Thaler	Tel Aviv Sourasky Medical Center	Tel Aviv-Yafo	
Italy	Andrea Quattrone	Magna Græcia University of Catanzaro	Catanzaro	
	Enza Maria Valente	University of Pavia	Pavia	
	Grazia Annesi	National Research Council	Cosenza	
	Lucilla Parnetti	University of Perugia	Perugia	
	Micol Avenali	University of Pavia	Pavia	
	Monica Gagliardi	Magna Graecia University	Catanzaro	
	Tommaso Schirinzi	University of Rome Tor Vergata	Rome	
	Caterina Galandra	IRCCS Mondino Foundation	Pavia	
	Anna De Rosa	University of Naples Federico II	Naples	
Japan	Manabu Funayama	Juntendo University	Tokyo	
	Nobutaka Hattori	Juntendo University faculty of medicine	Tokyo	
	Tomotaka Shiraishi	Jikei University School of Medicine	Tokyo	
Kazakhstan	Altynay Karimova	Institute of Neurology and Neurorehabilitation	Almaty	
	Gulnaz Kaishibayeva	Institute of Neurology and Neurorehabilitation	Almaty	
	Aigerim Utegenova	West Kazakhstan Marat Ospanov State Medical Univ	Aktobe	
	Aigul Yemagambetova	West Kazakhstan Marat Ospanov State Medical Univ	Aktobe	
	Vadim Akhmetzhanov	Medline medical center	Astana	
	Seitzhan Aidarov	National Center for Neurosurgery	Astana	
	Tautanova Raushan	Astana Medical University	Astana	

Country	Name	Institution	City	State/Province
	Bagyzhan Syzdykova Syzdykova	City multi-field hospital No. 1	Astana	
	Dinara Alzhanova	Astana Medical University	Astana	
	Zhanybek Myrzayev	International University of Postgraduate Education	Almaty	
	Saltanat Abdraimova	South Kazakhstan Medical Academy	Shymkent	
**Kyrgyzstan**	Cholpon Shambetova	Kyrgyz State Medical Academy	Bishkek	
**Luxembourg**	Rejko Krüger	University of Luxembourg	Esch-sur-Alzette	
	Patrick May	University of Luxembourg	Esch-sur-Alzette	
**Malaysia**	Ai Huey Tan	University of Malaya	Kuala Lumpur	
	Azlina Ahmad-Annuar	University of Malaya	Kuala Lumpur	
	Mohamed Ibrahim Norlinah	Universiti Kebangsaan Malaysia	Selangor	
	Nor Azian Abdul Murad	UKM Medical Molecular Biology Institute	Kuala Lumpur	
	Shahrul Azmin	Universiti Kebangsaan Malaysia Medical Centre	Kuala Lumpur	
	Shen-Yang Lim	University of Malaya	Kuala Lumpur	
	Wael Mohamed	International Islamic University	Kuala Lumpur	
	Yi Wen Tay	University of Malaya	Kuala Lumpur	
	Azalea Tenerife Pajo	University of Malaya	Kuala Lumpur	
**Mexico**	Daniel Martinez-Ramirez	Tecnologico de Monterrey	Monterrey	
	Mayela Rodriguez-Violante	Instituto Nacional de Neurologia y Neurocirugia	Mexico City	
	Paula Reyes-Pérez	Universidad Nacional Autónoma de México	Santiago de	
	Alejandra Medina Rivera	Universidad Nacional Autónoma de México	Santiago de	
	Nancy Monroy Jaramillo	Instituto Nacional de Neurología y Neurocirugía	Mexico City	
**Mongolia**	Bayasgalan Tserensodnom	Mongolian National University of Medical Sciences	Ulaanbaatar	
**Nepal**	Rajeev Ojha	Tribhuvan University	Kirtipur	
**Netherlands**	Wilma Van De Berg	Vanderbilt University Medical Center	Amsterdam	
	Bas Bleom	Radboud University	Nijmegen	
	Bart Van de Warrenburg	Radboud University Medical Center	Nijmegen	
	Lisette Charbonnier	Brain Research and Innovation Center	Amsterdam	
**New Zealand**	Tim J. Anderson	University of Otago	Dunedin	
	Toni L. Pitcher	University of Otago	Dunedin	
**Nigeria**	Arinola Sanyaolu	University of Lagos	Lagos	
	Njideka Okubadejo	University of Lagos	Lagos	
	Oluwadamilola Ojo	College of Medicine of the University of Lagos	Lagos	
	Simon Izuchukwu Ozomma	University of Calabar Teaching Hospital	Calabar	
	Kolawole Wahab	University of Ilorin	Ilorin	
**Norway**	Jan O. Aasly	Norwegian University of Science and Technology	Trondheim	
	Lasse Pihlstrøm	Oslo University Hospital	Oslo	
	Manuela Tan	Oslo University Hospital	Oslo	
	Ingeborg Haugesag Lie	Oslo University Hospital	Oslo	
	Jodi Maple-Grødem	Stavanger University Hospital	Stavanger	
**Pakistan**	Shoaib Ur-Rehman	University of Science and Technology Bannu	Bannu	

Country	Name	Institution	City	State/Province
**Peru**	Mario Cornejo-Olivas	Universidad Cientifica del Sur	Lima	
**Philippines**	Maria Leila Doquenia	Metropolitan Medical Center	Manila	
	Raymond Rosales	Metropolitan Medical Center	Manila	
**Puerto Rico**	Angel Vinuela	University of Puerto Rico	San Juan	
**Russia**	Elena Iakovenko	Research Center of Neurology	Moscow	
	Anna Gareeva	Ufa Federal Research Center	Ufa	
	Gulnara Akhmadeeva	Ufa Scientific Center	Ufa	
	Irina Gilyazova	Russian Academy of Sciences / Bashkir State Medica	Ufa	
**Saudi Arabia**	Bashayer Al Mubarak	King Faisal Specialist Hospital and Research Center	Riyadh	
	Muhammad Umair	King Abdullah International Medical Research	Jeddah	
**Singapore**	Eng-King Tan	National Neuroscience Institute	Singapore	
	Jia Nee Foo	Nanyang Technological University	Singapore	
	Elaine Chew	Nanyang Technological University	Singapore	
**Slovenia**	Vesna van Midden	Ljubljana University Medical Centre	Ljubljana	
**South Africa**	Ferzana Amod	University of KwaZulu-Natal	Durban	
	Jonathan Carr	University of Stellenbosch	Stellenbosch	
	Soraya Bardien	Stellenbosch University	Stellenbosch	
	Nikita Pillay	University of the Western Cape	Bellville	
	Kathryn Step	Stellenbosch University	Cape Town	
**South Korea**	Beomseok Jeon	Seoul National University Hospital	Seoul	
	Yun Joong Kim	Yongin Severance Hospital	Seoul	
	Jung Hwan Shin	Seoul National University	Seoul	
	Joowon Jang	Seoul National University	Seoul	
**Spain**	Esther Cubo	Hospital Universitario Burgos	Burgos	
	Ignacio Alvarez	University Hospital Mutua Terrassa	Barcelona	
	Janet Hoenicka	Institut de Recerca Sant Joan de Deu	Barcelona	
	Katrin Beyer	Research Institute Germans Trias i Pujol	Barcelona	
	Maria Teresa Periñan	Instituto de Biomedicina de Sevilla	Seville	
	Pau Pastor	University Hospital Germans Trias i Pujol	Barcelona	
	Ruben Fernandez-Santiago	Hospital Clínic de Barcelona	Barcelona	
	Pilar Gómez Garre	Instituto de Biomedicina de Sevilla	Seville	
	Pablo Mir	Instituto de Biomedicina de Sevilla	Seville	
**Sudan**	Sarah El-Sadig	Faculty of medicine university of Khartoum	Khartoum	
**Sweden**	Kajsa Brolin	Lund University	Lund	
	Per Svenningsson	Karolinska Institute	Stockholm	
	Maria Swanberg	Lund University	Lund	
**Switzerland**	Christiane Zweier	Inselspital Bern, University of Bern	Bern	
	Gerd Tinkhauser	University Hospital Bern	Bern	
	Paul Krack	Inselspital Bern, University of Bern	Bern	
	Chin-Hsien Lin	National Taiwan University Hospital	Taipei City	

Country	Name	Institution	City	State/Province
Taiwan	Hsiu-Chuan Wu	Chang Gung Memorial Hospital	Taoyuan City	
	Pin-Jui Kung	National Taiwan University	Taipei City	
	Ruey-Meei Wu	National Taiwan University Hospital	Taipei City	
	Yihru Wu	Chang Gung Memorial Hospital	Taoyuan City	
Tajikistan	Ganieva Manizha	Avicenna Tajik State Medical University	Dushanbe	
Tunisia	Rim Amouri	National Institute Mongi Ben Hamida of Neurology	Tunis	
	Samia Ben Sassi	Mongi Ben Hmida National Institute of Neurology	Tunis	
Turkey	A. Nazlı Başak	Koç University	Istanbul	
	Gencer Genc	Şişli Etfal Training and Research Hospital	Istanbul	
	Özgür Öztop Çakmak	Koç University	Istanbul	
	Sibel Ertan	Koç University	Istanbul	
United Kingdom	Alastair Noyce	Queen Mary University of London	London	
	Alejandro Martínez-Carrasco	University College London	London	
	Anette Schrag	University College London	London	
	Anthony Schapira	University College London	London	
	Camille Carroll	University of Plymouth	Plymouth	
	Donald Grosset	University of Glasgow	Glasgow	
	Eleanor J. Stafford	University College London	London	
	Henry Houlden	University College London	London	
	Huw R Morris	University College London	London	
	John Hardy	University College London	London	
	Kin Ying Mok	Univeristy College London	London	
	Mie Rizig	University College London	London	
	Nicholas Wood	University College London	London	
	Nigel Williams	Cardiff University	Cardiff	
	Olaitan Okunoye	University College London	London	
	Rauan Kaiyrzhanov	University College London	London	
	Rimona Weil	University College London	London	
	Seth Love	University of Bristol	Bristol	
	Simona Jasaityte	University College London	London	
	Sumit Dey	Queen Mary University of London	London	
	Vida Obese	University College London	London	
	Spencer Finch	Queen Mary University of London	London	
	Valentina Escott-Price	Cardiff University	Cardiff	
	Hamin Lee	St George's, University of London	London	
	Roger Barker	University of Cambridge	Cambridge	
	Mina Ryten	University College London	London	
	Michele Hu	University of Oxford	Oxford	
	Laura Parkkinen	University of Oxford	Oxford	
	Kailash Bhatia	University College London	London	

Country	Name	Institution	City	State/Province
	Richard Walker	Northumbria Healthcare at NHS Foundation Trust	Newcastle upon Tyne	
	Steve Gentleman	Imperial College London	London	
	Thomas Warner	University College London	London	
	David Burn	Newcastle University	Newcastle upon Tyne	
	Christian Lambert	Imperial College London	London	
	Caroline Williams-Gray	University of Cambridge	Cambridge	
	Chris Morris	Newcastle University	Newcastle upon Tyne	
	Deborah Attuah	YLD	London	
	Raquel Real	University College London	London	
	Yen Tai	Imperial College London	London	
	Alberto Espay	University of Cincinnati	Cincinnati	Ohio
	Alyssa O'Grady	The Michael J. Fox Foundation for Parkinson's	New York	New York
	Andrew B Singleton	National Institute on Aging	Bethesda	Maryland
	Andrew K. Sobering	Augusta University / University of Georgia Medical	Augusta	Georgia
	Bernadette Siddiqi	The Michael J. Fox Foundation for Parkinson's	New York	New York
	Bradford Casey	The Michael J. Fox Foundation for Parkinson's	New York	New York
	Brian Fiske	The Michael J. Fox Foundation for Parkinson's	New York	New York
	Cabell Jonas	Mid-Atlantic Permanente Medical Group	Bethesda	Maryland
	Carlos Cruchaga	Washington University	St. Louis	Missouri
	Caroline B. Pantazis	National Institutes of Health	Bethesda	Maryland
	Charisse Comart	The Michael J. Fox Foundation for Parkinson's	New York	New York
	Claire Wegel	Indiana University	Bloomington	Indiana
	Cornelis Blauwendraat	National Institutes of Health	Bethesda	Maryland
	Dan Vitale	National Institutes of Health	Bethesda	Maryland
	Deborah Hall	Rush University	Chicago	Illinois
	Dena Hernandez	National Institutes of Health	Bethesda	Maryland
	Ejaz Shiamim	Kaiser Permanente	Oakland	California
	Ekemini Riley	Coalition for Aligning Science	Washington	Washington
	Faraz Faghri	National Institutes of Health	Bethesda	Maryland
	Geidy E. Serrano	Banner Sun Health Research Institute	Sun City	Arizona
	Hampton Leonard	National Institute on Aging/National Institutes of	Bethesda	Maryland
	Hirotaka Iwaki	Data Tecnica International	Washington	Washington
	Honglei Chen	Michigan State University	East Lansing	Michigan
	Ignacio F. Mata	Cleveland Clinic	Cleveland	Ohio
	Ignacio Juan Keller Sarmiento	Northwestern University	Evanston	Illinois
	Jared Williamson	Kaiser Permanente	Oakland	California
	Jonggeol Jeff Kim	National Institutes of Health	Bethesda	Maryland
	Joseph Jankovic	Baylor College of Medicine	Houston	Texas
	Joshua Shulman	Baylor College of Medicine / Texas Children's	Houston	Texas
	Justin C. Solle	The Michael J. Fox Foundation for Parkinson's	New York	New York

Country	Name	Institution	City	State/Province
USA	Kaileigh Murphy	The Michael J. Fox Foundation for Parkinson's	New York	New York
	Kamalini Ghosh Galvelis	Parkinson's Foundation	Princeton	New Jersey
	Karen Nuytemans	University of Miami Miller School of Medicine	Miami	Florida
	Karl Kieburtz	Beth Israel Deaconess Medical Center	Boston	Massachusetts
	Kate Andersh	National Institute on Aging	Bethesda	Maryland
	Katerina Markopoulou	North Shore University Health System	Chicago	Illinois
	Kenneth Marek	Institute for Neurodegenerative Disorders	New Haven	Connecticut
	Kristin S. Levine	Data Tecnica International	Washington	Washington
	Lana M. Chahine	University of Pittsburgh	Pittsburgh	Pennsylvania
	Laura Ibanez	Washington University	Saint Louis	Missouri
	Laurel Screven	National Institute on Aging	Bethesda	Maryland
	Lauren Ruffrage	University of Alabama at Birmingham	Birmingham	Alabama
	Lisa Shulman	University of Maryland	Baltimore	Maryland
	Luca Marsili	University of Cincinnati	Cincinnati	Ohio
	Maggie Kuhl	The Michael J. Fox Foundation for Parkinson's	New York	New York
	Marissa Dean	University of Alabama at Birmingham	Birmingham	Alabama
	Mary B Makarious	National Institutes of Health	Bethesda	Maryland
	Matthew Farrer	University of Florida - Neurology	Gainesville	Florida
	Mathew Koretsky	National Institutes of Health	Bethesda	Maryland
	Megan J. Puckelwartz	Northwestern University	Chicago	Illinois
	Miguel Inca-Martinez	Cleveland Clinic	Cleveland	Ohio
	Mike A. Nalls	National Institutes of Health	Bethesda	Maryland
	Naomi Louie	The Michael J. Fox Foundation for Parkinson's	New York	New York
	Niccolò Emanuele Mencacci	Northwestern University	Evanston	Illinois
	Roger Albin	Universit of Michigan	Ann Arbor	Michigan
	Roy Alcalay	Columbia University	New York	New York
	Ruth Walker	James J. Peters Veterans Affairs Medical Center	New York	New York
	Sara Bandres-Ciga	National Institutes of Health	Bethesda	Maryland
	Sohini Chowdhury	The Michael J. Fox Foundation for Parkinson's	New York	New York
	Sonya Dumanis	Aligning Science Across Parkinson's	Washington	Washington
	Steven Lubbe	Northwestern University	Chicago	Illinois
	Tao Xie	University of Chicago	Chicago	Illinois
	Tatiana Foroud	Indiana University School of Medicine	Indianapolis	Indiana
	Thomas Beach	Sun Health Research Institution	Sun City	Arizona
	Todd Sherer	The Michael J Fox Foundation for Parkinson's	New York	New York
	Yeajin Song	National Institutes of Health	Bethesda	Maryland
	Dana Lewis	Aligning Science Across Parkinson's	Baltimore	Maryland
	Shreya Menon	Gladstone Institutes	San Francisco	California
	Melissa Nirenberg	Icahn School of Medicine at Mount Sinai	New York	New York
	Spencer Grant	National Institutes of Health	Bethesda	Maryland

Country	Name	Institution	City	State/Province
	Shannon Ballard	National Institutes of Health	Bethesda	Maryland
	Chad Shaw	Baylor College of Medicine	Houston	Texas
	Sidra Aslam	Banner Health	Phoenix	Arizona
	Geidy Serrano	Banner Sun Health Research Institute	Sun City	Arizona
	Devin Sharp	Aligning Science Across Parkinson's	Vancouver	British Columbia
	Kensuke Daida	National Institute of Aging	Bethesda	Maryland
	Rachel Saunders-Pullman	Icahn School of Medicine at Mount Sinai	New York	New York
	Michiko Kimura Bruno	The Queen's Medical Center	Honolulu	Hawaii
	Matt Farrer	University of Florida College of Medicine	Gainesville	Florida
	Haydeh Payami	The University of Alabama at Birmingham Heersink S	Birmingham	Alabama
	Ryan Pfingst	The Michael J Fox Foundation	New York	New York
	Albin Roger	University of Michigan	Ann Arbor	Michigan
	James B Leverenz	Cleveland Clinic	Cleveland	Ohio
	Elizabeth Disbrow	LSU Health Shreveport	Shreveport	Louisiana
	Debi Brooks	The Michael J Fox Foundation	New York	New York
	Randy Schekman	University of California, Berkeley	Berkeley	California
	Un Kang	NYU Grossman School of Medicine	New York	New York
	Zbigniew K. Wszolek	Mayo Clinic College of Medicine	Rochester	Minnesota
	Cyrus Zabetian	VA Puget Sound Health Care System	Seattle	Washington
	Zach Chaney	The Michael J Fox Foundation	New York	New York
	Mark Cookson	National Institute of Health	Bethesda	Maryland
	Christine Swanson-Fischer	National Institute of Health	Bethesda	Maryland
	Conor Hennessey	The Michael J Fox Foundation	New York	New York
	Cassandra Barrett	The Michael J Fox Foundation	New York	New York
	Beate Ritz	University of California, Los Angeles	Los Angeles	California
	Bradley Boeve	Mayo Clinic	Rochester	Minnesota
	Ashley Rawls	University of Florida College of Medicine	Gainesville	Florida
**Vietnam**	Duan Nguyen	Hue University	Huế	
	Toan Nguyen	Hue University	Huế	
**Zambia**	Masharip Atadzhanov	University of Zambia	Lusaka	

Country	Funders and Disclosures
**Algeria**	Nothing to declare
**Argentina**	Nothing to declare
	Nothing to declare
	Nothing to declare
**Armenia**	Nothing to declare
	Nothing to declare
	Nothing to declare
**Australia**	The Sydney Brain Bank is located at and supported by Neuroscience Research Australia
	Paul Ainsworth Family Foundation
	Nothing to declare
	The Australian Parkinson's Genetics Study is supported by the Shake It Up Australia Foundation and The Michael J. Fox Foundation for Parkinson's Rese
	Nothing to declare
	Nothing to declare
	Nothing to declare
	Nothing to declare
	Nothing to declare
	Nothing to declare
**Austria**	Nothing to declare
**Azerbaijan**	Nothing to declare
**Belgium**	Nothing to declare
**Brazil**	Nothing to declare
	Nothing to declare
	Global Parkinson's Genetics Program
	Nothing to declare
	Nothing to declare
	Nothing to declare
**Canada**	Nothing to declare
	CIHR, Brain Canada, Parkinson Canada
	Nothing to declare
	Nothing to declare
	Nothing to declare
	Nothing to declare
	Nothing to declare
**Chile**	Nothing to declare
	Nothing to declare
	Nothing to declare
	Nothing to declare
	Nothing to declare
	Nothing to declare
	Nothing to declare
	Nothing to declare

Country	Funders and Disclosures
China	Nothing to declare
	Nothing to declare
	Nothing to declare
	Nothing to declare
Colombia	Nothing to declare
	Nothing to declare
	Nothing to declare
Costa Rica	Nothing to declare
Denmark	Nothing to declare
Egypt	The AUC/ ASRT/ DAAD
	Nothing to declare
Ethiopia	Nothing to declare
France	Nothing to declare
	Nothing to declare
	Nothing to declare
Georgia	Nothing to declare
	Nothing to declare
Germany	Nothing to declare
	Nothing to declare
	Nothing to declare
	CK serves as a medical Advisor to Centogene on genetic testing reports in the field of movement disorders, except Parkinson's disease, and is a membe
	Nothing to declare
	Nothing to declare
	Nothing to declare
	Nothing to declare
	Nothing to declare
	Johanna Junker is funded by the MJFF Data Community Innovators Program and received a Family Mobility Grant from the University of Luebeck
	Nothing to declare
	Nothing to declare
	Dr. Sharma is further funded by the Michael J Fox Foundation, USA Genetic Diversity in PD Program: GAP-India Grant ID: 009411.
	Nothing to declare
	Nothing to declare
	Nothing to declare
	Former employee of 23andMe, Inc. Owns stock and/or stock options in 23andMe, Inc.
	Nothing to declare
	Nothing to declare
	Nothing to declare
	Nothing to declare
	Nothing to declare
Ghana	Nothing to declare

Country	Funders and Disclosures
Greece	Nothing to declare
	Nothing to declare
	Nothing to declare
	Nothing to declare
	PPMI2 (funded by MJFF), ALAMEDA (H2020 grant), funding by HFRI
	The non-profit organisation for scientific research in Parkinson’s disease and related disorders
	Nothing to declare
Honduras	Nothing to declare
Hong Kong	Nothing to declare
	Nothing to declare
	Nothing to declare
	Nothing to declare
	Nothing to declare
India	Michael J. Fox Foundation
	Nothing to declare
	Nothing to declare
	Nothing to declare
	Nothing to declare
	Nothing to declare
Iran	Nothing to declare
Israel	Nothing to declare
	Nothing to declare
Italy	Nothing to declare
	Nothing to declare
	Nothing to declare
	Nothing to declare
	Nothing to declare
	Nothing to declare
	Nothing to declare
	Nothing to declare
	Nothing to declare
Japan	Nothing to declare
	Nothing to declare
	Nothing to declare
Kazakhstan	Nothing to declare
	Nothing to declare
	Nothing to declare
	Nothing to declare
	Nothing to declare
	Nothing to declare
	Nothing to declare

Country	Funders and Disclosures
	Nothing to declare
	Nothing to declare
	Nothing to declare
	Nothing to declare
**Kyrgyzstan**	Nothing to declare
**Luxembourg**	Nothing to declare
	Nothing to declare
**Malaysia**	Nothing to declare
	Nothing to declare
	Nothing to declare
	Nothing to declare
	Nothing to declare
	Nothing to declare
	Nothing to declare
	Nothing to declare
	Nothing to declare
**Mexico**	Nothing to declare
	Nothing to declare
	Global Parkinson's Genetics Program
	Nothing to declare
	Nothing to declare
**Mongolia**	Nothing to declare
**Nepal**	Nothing to declare
**Netherlands**	Nothing to declare
	Nothing to declare
	Nothing to declare
	Nothing to declare
**New Zealand**	Heath Research Council of New Zealand; Ministry of Business Innovation and Employment (New Zealand), Neurological Foundation of New Zealand
	Funding - Health Research Council of New Zealand
**Nigeria**	Nothing to declare
	Michael J Fox Foundation; Tertiary Education Trust Fund (TETFUND) National Research Fund
	Nothing to declare
	Nothing to declare
	Nothing to declare
**Norway**	Nothing to declare
	Southeastern Regional Health Authority, Norway and Michael J. Fox Foundation
	Southeastern Norway Regional Health Authority Norway and Michael J. Fox Foundation
	Nothing to declare
	Nothing to declare
**Pakistan**	Nothing to declare

Country	Funders and Disclosures
**Peru**	Michael J. Fox Foundation for Parkinson's Research and Aligning Science Across Parkinson's Initiative
**Philippines**	Nothing to declare
	Nothing to declare
**Puerto Rico**	Nothing to declare
**Russia**	Nothing to declare
	Nothing to declare
	Nothing to declare
	Nothing to declare
**Saudi Arabia**	Nothing to declare
	Nothing to declare
**Singapore**	Singapore National Medical Research Council (MOH-OFLCG-000207)
	Singapore National Medical Research Council (MOH-000559)
	Nothing to declare
**Slovenia**	Nothing to declare
**South Africa**	Nothing to declare
	Nothing to declare
	National Research Foundation of South Africa[Grant Number 129249]
	Nothing to declare
	Nothing to declare
**South Korea**	Nothing to declare
	Nothing to declare
	Nothing to declare
	Nothing to declare
**Spain**	Nothing to declare
	Nothing to declare
	Fondo de Investigación Sanitaria, Instituto Salud Carlos III, Grant PI019/00126
	Nothing to declare
	Nothing to declare
	Nothing to declare
	Nothing to declare
	Nothing to declare
	Nothing to declare
**Sudan**	Nothing to declare
**Sweden**	Nothing to declare
	Nothing to declare
	Nothing to declare
**Switzerland**	Nothing to declare
	Nothing to declare
	Nothing to declare
	Nothing to declare

Country	Funders and Disclosures
Taiwan	Nothing to declare
	Global Parkinson's Genetics Program
	Minister of Science and Technology, Taiwan Government; National Taiwan University; Parkinson foundation (USA); Michael J. Fox Foundation
	Nothing to declare
Tajikistan	Nothing to declare
Tunisia	Nothing to declare
	Nothing to declare
Turkey	Kirac Foundation and Koc Univ.
	Nothing to declare
	Nothing to declare
	Nothing to declare
United Kingdom	Prof. Noyce reports grants from Parkinson's UK, Barts Charity, Cure Parkinson's, NIHR, Innovate UK, Virginia Keiley benefaction, Alchemab, Aligning Sci
	Global Parkinson's Genetics Program
	NIHR UCL/H Biomedical Research Centre
	ASAP
	C Carroll receives salary from University of Plymouth, University Hospitals Plymouth NHS Trust and National Institute of Health Research; she has receiv
	Tracking Parkinson's (J-1101) is funded by Parkinson's UK
	Nothing to declare
	Nothing to declare
	Dr Morris is employed by UCL. In the last 12 months he reports paid consultancy from Roche and Amylyx ; lecture fees/honoraria - BMJ, Kyowa Kirin, Mo
	Nothing to declare
	Nothing to declare
	Nothing to declare
	ASAP- CRN
	Parkinson's UK
	Nothing to declare
	Nothing to declare
	Nothing to declare
	Medical Research Council (UK) MR/T018569/1
	Nothing to declare
	The Michael J. Fox Foundation for Parkinson's Research
	Nothing to declare
	Nothing to declare
	Nothing to declare
	Nothing to declare
	Nothing to declare
	Nothing to declare
	Nothing to declare
	Nothing to declare
	Nothing to declare

Country	Funders and Disclosures
	Nothing to declare
	Nothing to declare
	Nothing to declare
	Nothing to declare
	Nothing to declare
	Nothing to declare
	Newcastle University, Alzheimer's Society, Alzheimer's Research UK, Medical Research Council
	Nothing to declare
	Nothing to declare
	Nothing to declare
	Nothing to declare
	Nothing to declare
	Michael J Fox Foundation for Parkinson's disease Research and Aligning Science Across Parkinson's Initiative
	Nothing to declare
	Nothing to declare
	Nothing to declare
	Nothing to declare
	Nothing to declare
	National Institutes of Health (R01AG044546 (CC), P01AG003991(CC, JCM), RF1AG053303 (CC), RF1AG058501 (CC), U01AG058922 (CC), RF1AG07
	Nothing to declare
	Nothing to declare
	Nothing to declare
	Nothing to declare
	Nothing to declare
	Nothing to declare
	Nothing to declare
	Nothing to declare
	Nothing to declare
	F.F.'s participation in this research was supported in part by the Intramural Research Program of the NIH, National Institute on Aging (NIA), National Instit
	Banner Sun Health Research Institute Brain and Body Donation Program of Sun City, Arizona for the provision of human biological materials. The Brain a
	H.L.L is supported by a competitive contract awarded to Data Tecnica International LLC by the National Institutes of Health to support open science resea
	H.I is supported by a competitive contract awarded to Data Tecnica International LLC by the National Institutes of Health to support open science researc
	NIH/DoD/Parkinson Foundation/MSU Foundation/Gibby vs. Parky Foundation - No COI to disclose
	Funding from MJFF and NIH
	Nothing to declare
	Nothing to declare
	Nothing to declare
	Nothing to declare
	Collection of samples and data at Baylor College of Medicine was supported by Huffington Foundation.
	Nothing to declare

Country	Funders and Disclosures
USA	Nothing to declare
	Nothing to declare
	This work has been supported by the American Parkinson Disease Association and the Margaret Q. Landenberger Research Foundation.
	Nothing to declare
	Nothing to declare
	Nothing to declare
	Consultant for Michael J Fox Foundation, GE Healthcare, Roche, UCB, BIAL, Denali, Takeda, , Cerapsir, UCB, Biohaven, Neuron23, Aprinoia, Astellas, C
	Nothing to declare
	Nothing to declare
	Nothing to declare
	Nothing to declare
	Nothing to declare
	Nothing to declare
	Nothing to declare
	Nothing to declare
	Dr. Dean is an investigator in studies funded by Abbvie, Inc., Hoffmann-La Roche, CHDI Foundation, Inc., Annexon, Inc., Retrophin, Inc, Neurocrine Bios
	Nothing to declare
	Nothing to declare
	Nothing to declare
	Nothing to declare
	Nothing to declare
	M.A.N.'s participation in this project was part of a competitive contract awarded to Data Tecnica International LLC by the National Institutes of Health to su
	Nothing to declare
	Nothing to declare
	P50NS123067
	Dr. Alcalay is funded by the Micharl J. Fox Foundation and the Parkinson's Foundation. He received consultation fees from Avrobio, Caraway, GSK, Merc
	Research funding from the Department of Veterans Affairs (CSRD Merit Award CX002342), consulted for Teladoc, Inc. and received an honorarium from
	Nothing to declare
	Nothing to declare
	Nothing to declare
	Nothing to declare
	Nothing to declare
	The Michael J. Fox Foundation for Parkinson's Research
	Banner Sun Health Research Institute Brain and Body Donation Program of Sun City, Arizona for the provision of human biological materials. The Brain a
	Nothing to declare
	Nothing to declare
	Nothing to declare
	Nothing to declare
	Nothing to declare
	Nothing to declare

Country	Funders and Disclosures
	Nothing to declare
	Nothing to declare
	Nothing to declare
	Nothing to declare
	Nothing to declare
	Nothing to declare
	Nothing to declare
	Nothing to declare
	Nothing to declare
	Nothing to declare
	Nothing to declare
	P50NS123067; Parkinson's Foundation
	Nothing to declare
	Nothing to declare
	Nothing to declare
	Nothing to declare
	Nothing to declare
	Nothing to declare
	Nothing to declare
	Nothing to declare
	Nothing to declare
	Nothing to declare
	Nothing to declare
	Nothing to declare
	Nothing to declare
	Nothing to declare
	Nothing to declare
**Vietnam**	Nothing to declare
	Nothing to declare
**Zambia**	Nothing to declare
